# Identification of Novel Potential Tubulin-Targeting Heterocyclic Compounds via Integrated Virtual Screening and In Vitro Studies

**DOI:** 10.3390/ph19101637

**Published:** 2026-10-16

**Authors:** Yaroslava Deyak, Dmytro Khylyuk, Rostyslav Dudchak, Nataliya Finiuk, Iryna Ivasechko, Yuliia Kozak, Nazar Solodiak, Halyna Derkach, Marta Pietruszyńska, Magdalena Podolak, Agnieszka Gornowicz, Krzysztof Bielawski, Anna Bielawska, Serhii Holota, Roman Lesyk

**Affiliations:** 1 Department of Pharmaceutical, Organic and Bioorganic Chemistry, Danylo Halytsky Lviv National Medical University, Pekarska 69, 79010 Lviv, Ukraine; deyak.yaroslava@gmail.com (Y.D.); solodyacknazar@gmail.com (N.S.); dr_r_lesyk@org.lviv.net (R.L.); 2 Department of Pharmaceutical Disciplines, Uzhhorod National University, Narodna Square 3, 88000 Uzhhorod, Ukraine; 3 Chair and Department of Organic Chemistry, Medical University of Lublin, Chodzki 4a, 20-093 Lublin, Poland; 4 Department of Synthesis and Technology of Drugs, Medical University of Bialystok, Kilinskiego 1, 15-089 Bialystok, Poland; rostyslav.dudchak@sd.umb.edu.pl (R.D.); krzysztof.bielawski@umb.edu.pl (K.B.); 5 Department of Regulation of Cell Proliferation and Apoptosis, Institute of Cell Biology of National Academy of Sciences of Ukraine, Drahomanov14/16, 79005 Lviv, Ukraine; NataliyaFiniuk@gmail.com (N.F.); irynagrytsyna@gmail.com (I.I.); juliana.kozzak@gmail.com (Y.K.); 6 Molecular Design Center, Danylo Halytsky Lviv National Medical University, Pekarska 69, 79010 Lviv, Ukraine; 7 Department of Chemistry, Pharmaceutical Analysis and Postgraduate Education, Ivano-Frankivsk National Medical University Halytska St., 124K, 76008 Ivano-Frankivsk, Ukraine; derkachhalya@gmail.com; 8 Department of Clinical Pharmacology, Medical University of Bialystok, Kilinskiego 1, 15-089 Bialystok, Poland; marta.pietruszynska@umb.edu.pl; 9 Department of Biotechnology, Medical University of Bialystok, Kilinskiego 1, 15-089 Bialystok, Poland; magdalena.podolak@sd.umb.edu.pl (M.P.); agnieszka.gornowicz@umb.edu.pl (A.G.); anna.bielawska@umb.edu.pl (A.B.); 10 Department of Biotechnology and Cell Biology, University of Information Technology and Management in Rzeszow, St. Sucharskiego 2, 35-225 Rzeszow, Poland

**Keywords:** tubulin inhibitors, molecular docking, molecular dynamics, diarylpyrazoline, heterocyclic compounds, anticancer agents

## Abstract

**Background**: The development of novel tubulin-targeting agents remains a promising strategy in anticancer drug design. In the present study, novel heterocyclic tubulin-targeting compounds were identified using a complex in silico/in vitro workflow. **Methods**: An in-house library of approximately 8000 structurally diverse heterocyclic compounds was screened for antiproliferative activity, yielding 260 experimentally validated hits. These compounds were docked against the colchicine, vinca, and taxane binding sites of tubulin. Selected compounds were also tested for their effects on cellular β-tubulin protein levels in Jurkat cells. **Results**: Docking analysis indicated that 42 compounds matched or exceeded colchicine’s binding energy, 3 compounds were comparable to vinblastine, and 18 compounds surpassed paclitaxel, suggesting the colchicine and taxane sites as preferred targets. Five top-scoring compounds (Les-6370, Les-4272, Les-4309, Les-4403, and Les-1419) were further analyzed via molecular dynamics simulations, confirming stable binding—particularly at the colchicine site—and favorable hydrogen-bonding and lipophilic interactions. At 10 µM, Les-1419 and Les-4309 reduced total cellular β-tubulin protein levels in Jurkat cells by 57.73% and 54.31%, respectively, relative to the effect observed for the reference compound Combretastatin A-4. **Conclusions**: This study resulted in the identification of novel starting scaffolds with favorable tubulin interaction activity and predicted drug-like properties, representing structurally novel parent structures and serving as promising starting points for further structural optimization and analogue development.

## 1. Introduction

Microtubules are dynamic cytoskeletal structures assembled from α- and β-tubulin heterodimers, capable of polymerization and disassembly. Their properties stem from interactions with a wide range of regulatory proteins that modulate microtubule dynamics and organization, thereby influencing essential cellular processes such as division, motility, polarity, and intracellular trafficking. Due to these critical roles, tubulin is considered an attractive target for anticancer drug development [1]. Tubulin contains multiple binding sites, most of which are located on the β-tubulin subunit and are involved in the regulation of microtubule dynamics [2,3]. Natural products such as colchicine, vinca alkaloids, and paclitaxel bind to these sites, inhibiting microtubule polymerization and leading to cell cycle arrest and apoptosis [4,5,6]. Considerable research efforts have therefore focused on designing novel anticancer agents that mimic these compounds and target the same tubulin binding sites [7,8,9,10]. Many of these molecules have demonstrated promising preclinical activity, and some are currently undergoing clinical evaluation [1].

Despite the therapeutic success of existing tubulin-targeting agents, their clinical application faces several limitations. One major challenge is the development of drug resistance, which reduces long-term efficacy in cancer treatment [11]. The clinical application of established tubulin-targeting agents is limited by dose-dependent toxicity, particularly neurotoxicity, insufficient tumor selectivity, and development of drug resistance. The main resistance mechanisms include increased drug efflux by ATP-binding cassette transporters, alterations in β-tubulin isotype expression, tubulin mutations, and changes in microtubule dynamics [12]. These limitations support the continued search for novel tubulin-targeting compounds with improved selectivity and ability to overcome resistance.

Another concern is systemic toxicity, as many of these compounds affect not only cancer cells but also healthy tissues. Insufficient specificity of current inhibitors often leads to adverse side effects, including neurotoxicity and myelosuppression. These issues underscore the difficulty of achieving an optimal balance between potency and safety. Consequently, there is a clear need for novel tubulin inhibitors with improved selectivity and reduced toxicity [1,13]. In addition, the chemical diversity of currently available compounds is limited, which constrains the exploration of alternative binding modes and mechanisms. Discovering new chemical scaffolds could provide opportunities to overcome resistance and enhance pharmacological profiles [14]. Structurally innovative molecules may also allow for better interaction with less-explored binding sites on tubulin [15]. Overall, the search for new tubulin-targeting agents remains a critical goal in anticancer drug discovery [16].

Recent studies on tubulin-targeting agents increasingly employ integrated in silico and in vitro approaches to identify and characterize novel chemotypes with potential antitubulin activity [12,17,18]. Computational chemistry tools such as molecular docking and virtual screening enable rapid prioritization of compounds with potential affinity toward key tubulin binding sites. These methods significantly reduce time and costs associated with early-stage drug discovery while improving hit identification efficiency [19]. Nevertheless, computational predictions require experimental validation to confirm biological relevance. In vitro evaluation using cell-based antiproliferative assays provides essential confirmation of cytotoxic effects in cancer cell lines. Tubulin polymerization assays allow direct assessment of the impact of selected compounds on microtubule dynamics. The integration of computational screening with in vitro validation minimizes false-positive hits and supports rational lead selection. Overall, this combined strategy represents an efficient model for the discovery of new tubulin-targeting agents [20].

**Figure 1:**
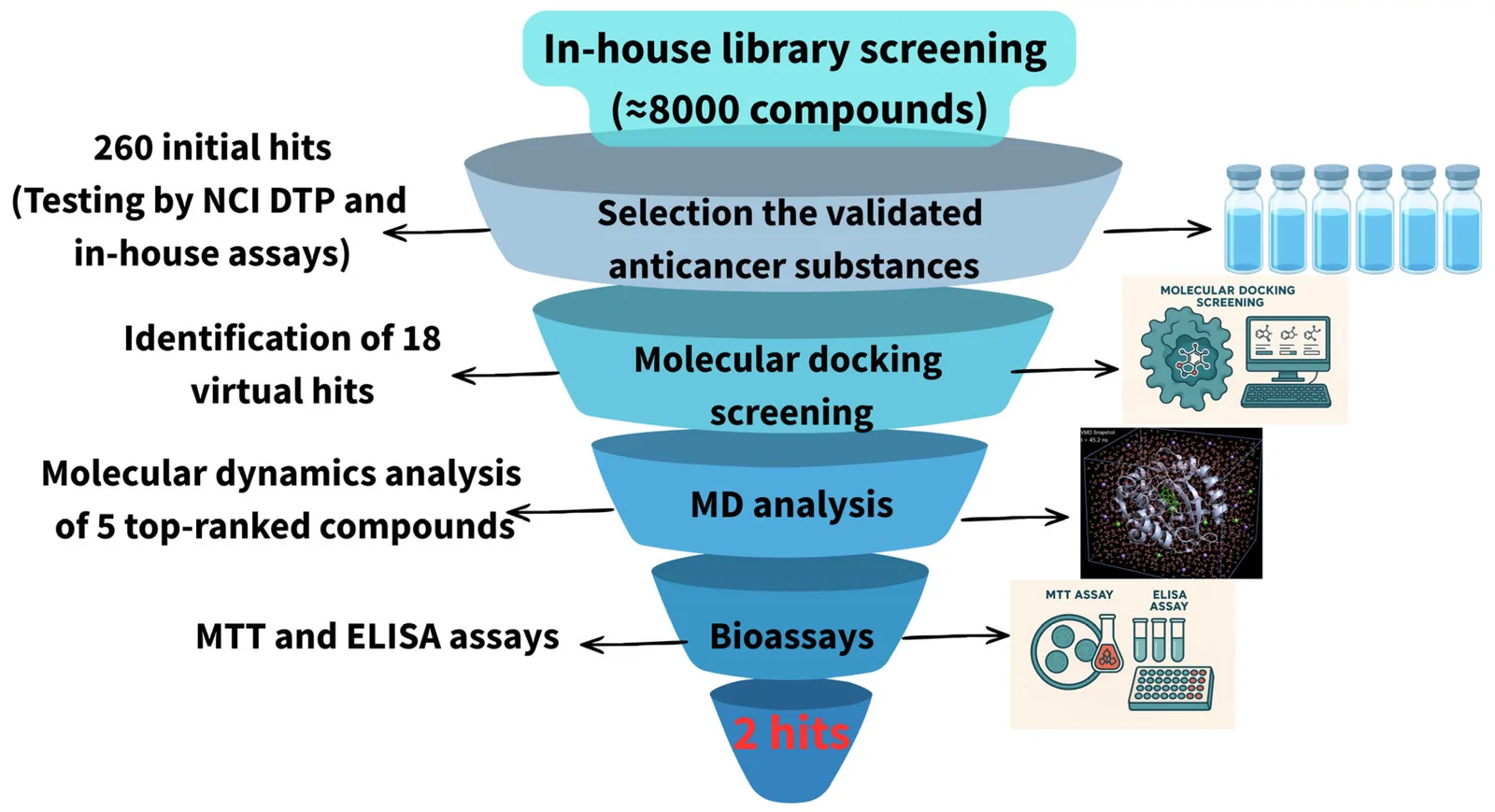
Applied workflow of the rational identification of novel tubulin-targeting compounds, combining computational and experimental approaches. The figure was created using Canva (Available online: https://www.canva.com/, accessed on 29 June 2026).

In the paper, we present studies based on an integrated in silico/in vitro approach and demonstrate a rational and efficient workflow for identifying novel tubulin-targeting agents, combining computational prioritization with experimental validation. The study design is depicted in Figure 1.

An in-house library of approximately 8 000 structure-diversified heterocyclic molecules, submitted for evaluation by the National Cancer Institute (NCI, USA), and in our biological laboratory for anticancer screening (MTT assay), served as the starting point for hit identification. A subset of compounds selected in the previous stage resulted in the identification of 260 in vitro validated anticancer hits. These hits were subsequently subjected to in silico molecular docking, from which 18 top-ranking compounds were selected. The five highest-ranking candidates were further investigated using molecular dynamics simulations and in vitro cytotoxicity assays. Based on the obtained biological results, the two most promising compounds were subsequently evaluated for their effects on β-tubulin levels in Jurkat cells using ELISA. In addition, the selected compounds were assessed in silico for drug-like properties and scaffold novelty, confirming their favorable pharmacokinetic profiles and originality as potential tubulin-targeting agents. Overall, the study provides new chemical scaffolds and a practical model for the discovery of anticancer agents targeting microtubule dynamics.

## 2. Results and Discussion

The identification of potential tubulin inhibitors was carried out through a structured, multi-stage screening workflow (Figure 1). Overall, from the library of approximately 8000 original synthetic heterocyclic structure-diversified compounds, a subset was screened in vitro, yielding 260 active compounds, which were then selected for subsequent in silico studies to evaluate their potential effects on tubulin dynamics. In the first stage, a part of the active compounds’ pool was formed based on the results of the in vitro screening performed by the National Cancer Institute (NCI, USA) against the standard NCI-60 human tumor cell line panel (the screening data for Les-4272, Les-4309 and Les-4403 are provided in Figures S24–S29), following the established Drug Evaluation Branch protocol. Compounds were classified as active based on their performance in the NCI-60 five-dose assay. A compound was considered active if it met one or more of the following criteria in at least one cell line with MGD-MID values of:

- GI_50_ ≤ 10 µM, indicating significant inhibition of cell proliferation;

- TGI ≤ 50 µM, indicating complete growth inhibition;

- LC_50_ ≤ 100 µM, indicating cytotoxicity resulting in 50% cell death.

Compounds that did not meet any of these criteria in any cell line were classified as inactive.

To ensure a comprehensive evaluation of the library and maximize structural diversity, a complementary in-house screening strategy was applied. Specifically, compounds that were not included in the NCI evaluation pool were tested using the in-house MTT cytotoxicity assay. Compounds exhibiting an IC_50_ during MTT test below 10 µM were classified as active and selected for further studies. These criteria allowed the identification of compounds with significant antiproliferative or cytotoxic effects for subsequent mechanistic and optimization analyses. To establish a rational connection between the observed biological effects and potential molecular mechanisms, the entire subset of 260 validated hits was subsequently subjected to an in silico screening workflow.

### 2.1. Molecular Docking Studies

**Figure 2:**
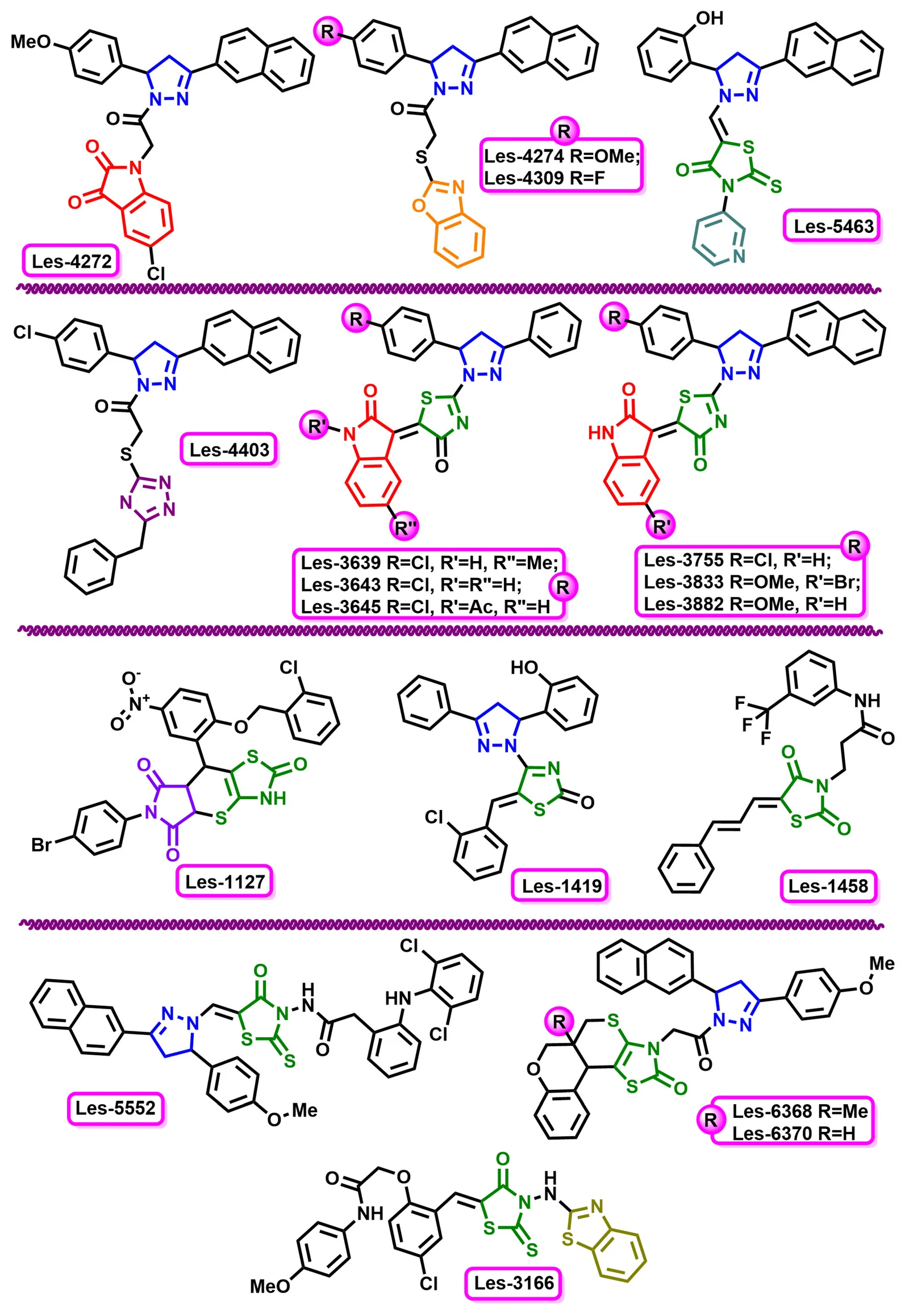
Molecular structures of virtual hits with potential tubulin-binding affinity.

Out of the 260 tested compounds, 42 demonstrated binding energies with tubulin that were equal to or lower than those of colchicine under the same docking parameters. The range of binding energies for ligands for the colchicine binding site was from −2.4 to −12.3 kcal/mol. In contrast, only 3 compounds have shown binding energies comparable to or slightly better than vinblastine at the vinca binding site (range: −5.4 to −11.7 kcal/mol). Additionally, 18 compounds demonstrated higher docking scores than paclitaxel at the taxane binding site (range: −5.1 to −11.1 kcal/mol). These observations suggest that the colchicine and taxane binding sites are the most preferable targets for the screened heterocyclic entities. The molecular structures of the 18 virtual hits toward the tubulin binding sites are presented in Figure 2.

The binding energies of the reference ligands and virtual hits with tubulin within their binding sites are summarized in Table 1.

To rigorously validate the docking screening results, we selected the five most promising compounds with the most favorable docking results across all three investigated binding sites. We also manually inspected the predicted binding poses and prioritized compounds with interaction patterns consistent with those observed for the corresponding native ligands. Based on the docking analysis, we prioritized five compounds (Les-6370, Les-4272, Les-4309, Les-4403, and Les-1419) for further investigation. Their favorable AutoDock Vina docking scores across the three binding sites suggest that these compounds may represent promising candidates for the development of potent tubulin-targeting agents, capable of forming multiple interactions with tubulin. Such a multi-site binding profile could potentially reduce the risk of treatment resistance, as mutational changes affecting a single binding site may not completely abolish the biological activity of these compounds.

**Figure 3:**
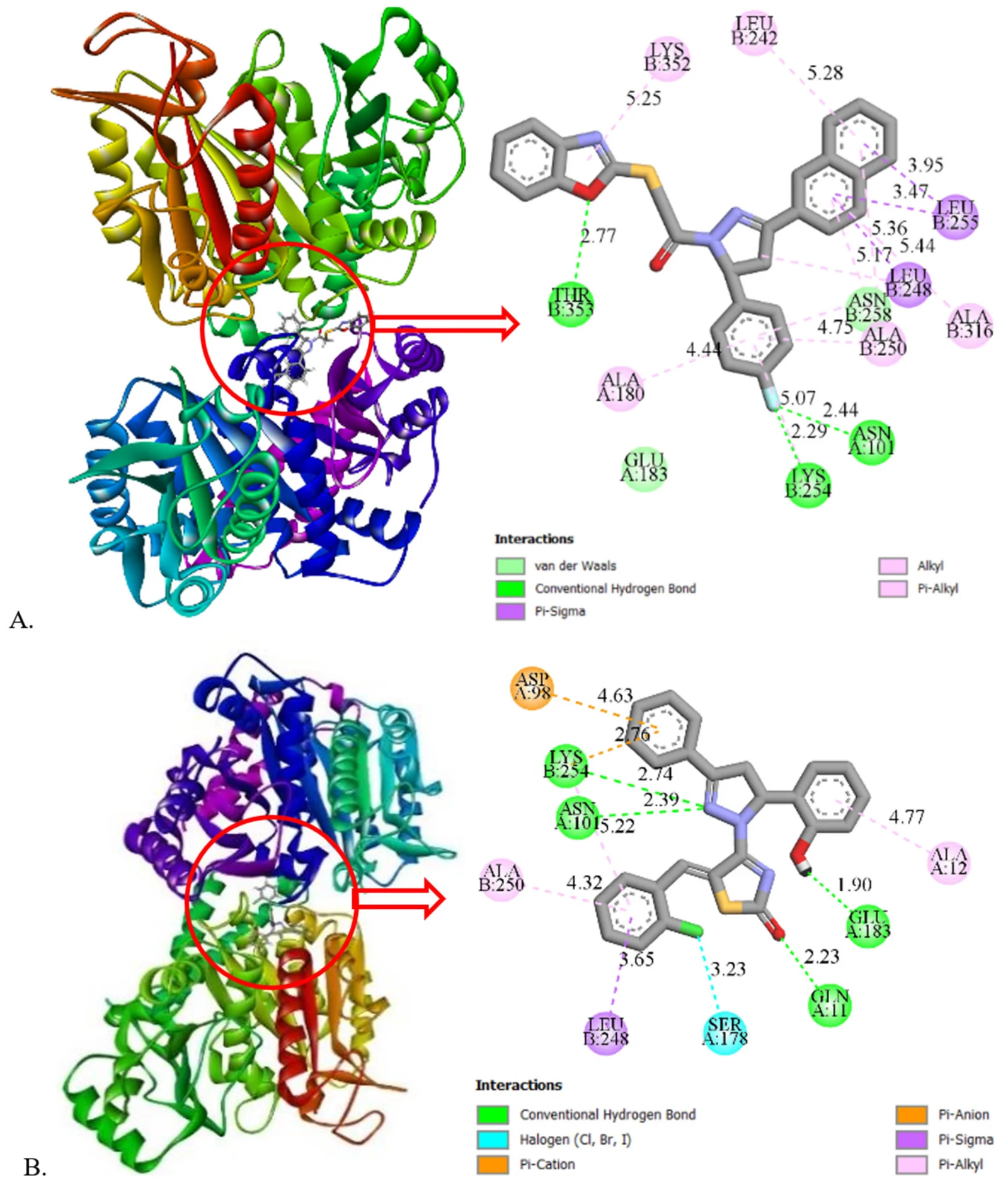
3D & 2D schemes of the (**A**) Les-4309, (**B**) Les-1419 interactions with tubulin in the colchicine binding site (PDB 4O2B).

The docking poses of molecules Les-6370, Les-4272, Les-4309, Les-4403, and Les-1419 were visualized, and interaction schemes are depicted in Figure 3 and Figure S1.

The docking poses showed that Les-4309 and Les-1419 occupy the colchicine-binding pocket at the interface of α- and β-tubulin. Their stabilization inside was mainly associated with hydrophobic contacts and several hydrogen bonds with the surrounding amino acid residues (Table 2). Despite some differences in ligand orientation, the compounds demonstrated binding patterns generally comparable to colchicine’s position inside the binding site.

Les-6370 forms two hydrogen bonds: one with Ala12 (2.39 Å) and Ser140 (2.13 Å) in a resonance-assisted manner, and another with Asn249 (2.81 Å). The 2-naphthyl substituent forms π-σ and π-alkyl lipophilic interactions with a cavity formed by five aliphatic amino acids. Additionally, two carbon–hydrogen bonds and two π-donor hydrogen bonds contribute to the overall binding energy of the Les-6370-tubulin complex (Figure S1).

The naphthyl moiety of compounds Les-4403, Les-4309, and Les-4272 is extensively integrated within the hydrophobic cavity constituted by aliphatic amino acid residues, located in the H7 loop of β-tubulin. This interaction underscores the significance of the lipophilic interactions facilitated by the naphthyl core within this protein domain. Conversely, Les-1419 exhibits a distinct interaction profile: although it lacks the pronounced hydrophobic interactions characteristic of the aforementioned compounds, this disparity is mitigated by the formation of three hydrogen bonds and a weak halogen bond. These interactions collectively compensate for the absence of a lipophilic moiety, highlighting a different mechanism of molecular engagement compared to the other structures discussed (Table 2).

**Figure 4:**
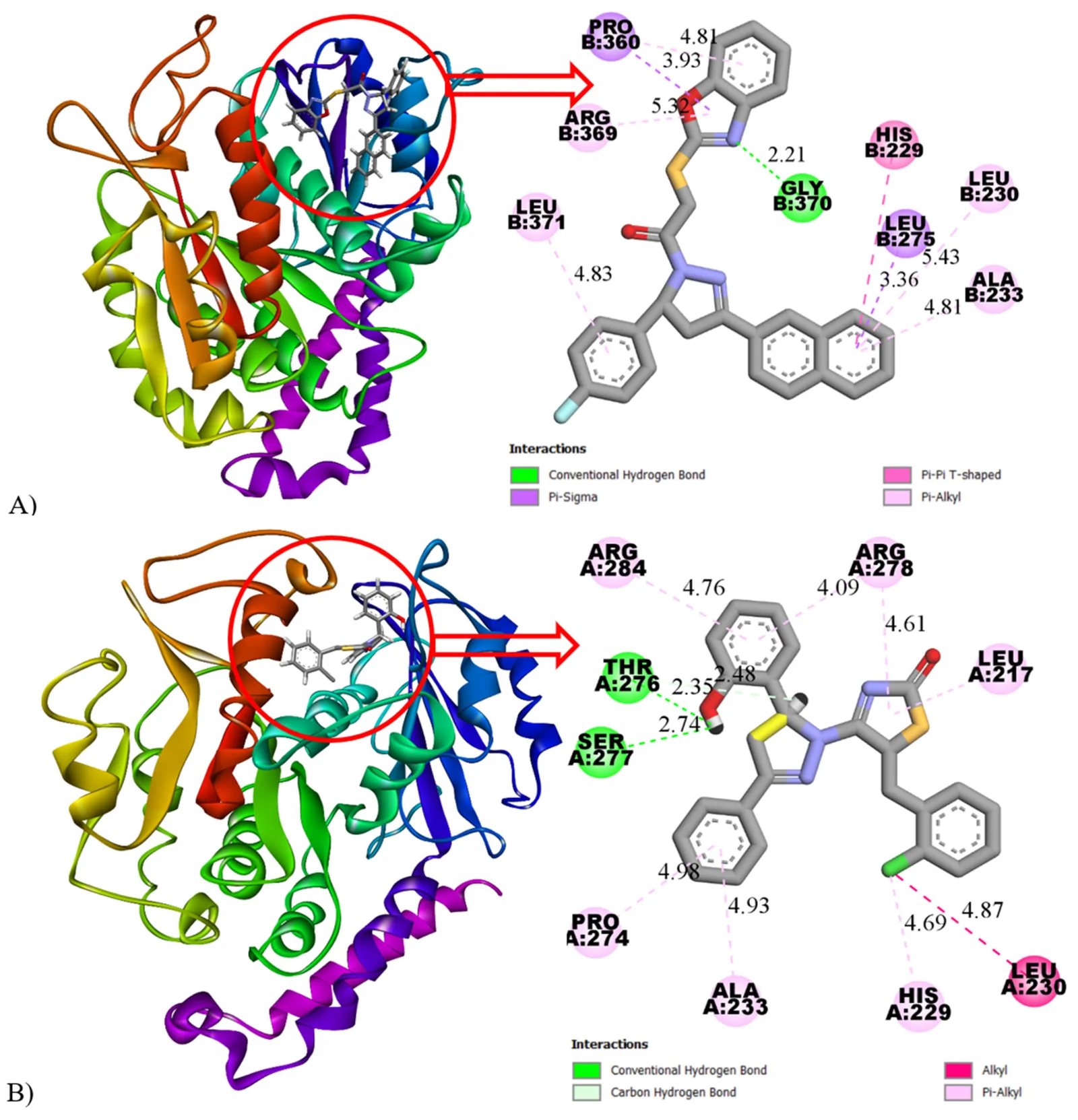
3D & 2D schemes of the (**A**) Les-4309, (**B**) Les-1419 interactions with tubulin in the taxane binding site (PDB 1JFF).

The taxane binding site is also an attractive target for the tested compounds. Within the taxane site, Les-6370 forms only one hydrogen bond with His229 (2.65 Å). Three aromatic cores (2-naphthyl, 4-methoxyphenyl, and benzodihydropyran moieties) interact with several aliphatic amino acids through π-alkyl, π-anion, and π-σ interactions (Figure 4 and Figure S3).

The interactions between other ligands and the taxane site are predominantly lipophilic, involving various hydrophobic interactions such as alkyl, π-alkyl, π-σ, π-π T-shaped, etc. Les-4403 and Les-4272 do not make any hydrogen bonds in their best-docked positions, while Les-4309 forms a single hydrogen bond with Gly370, whereas Les-1419 forms two hydrogen bonds with Thr276 and Ser277. The dominance of lipophilic interactions is similar to that of paclitaxel, which only forms one hydrogen bond with Thr276, with the remaining interactions being primarily lipophilic (Table 3).

**Figure 5:**
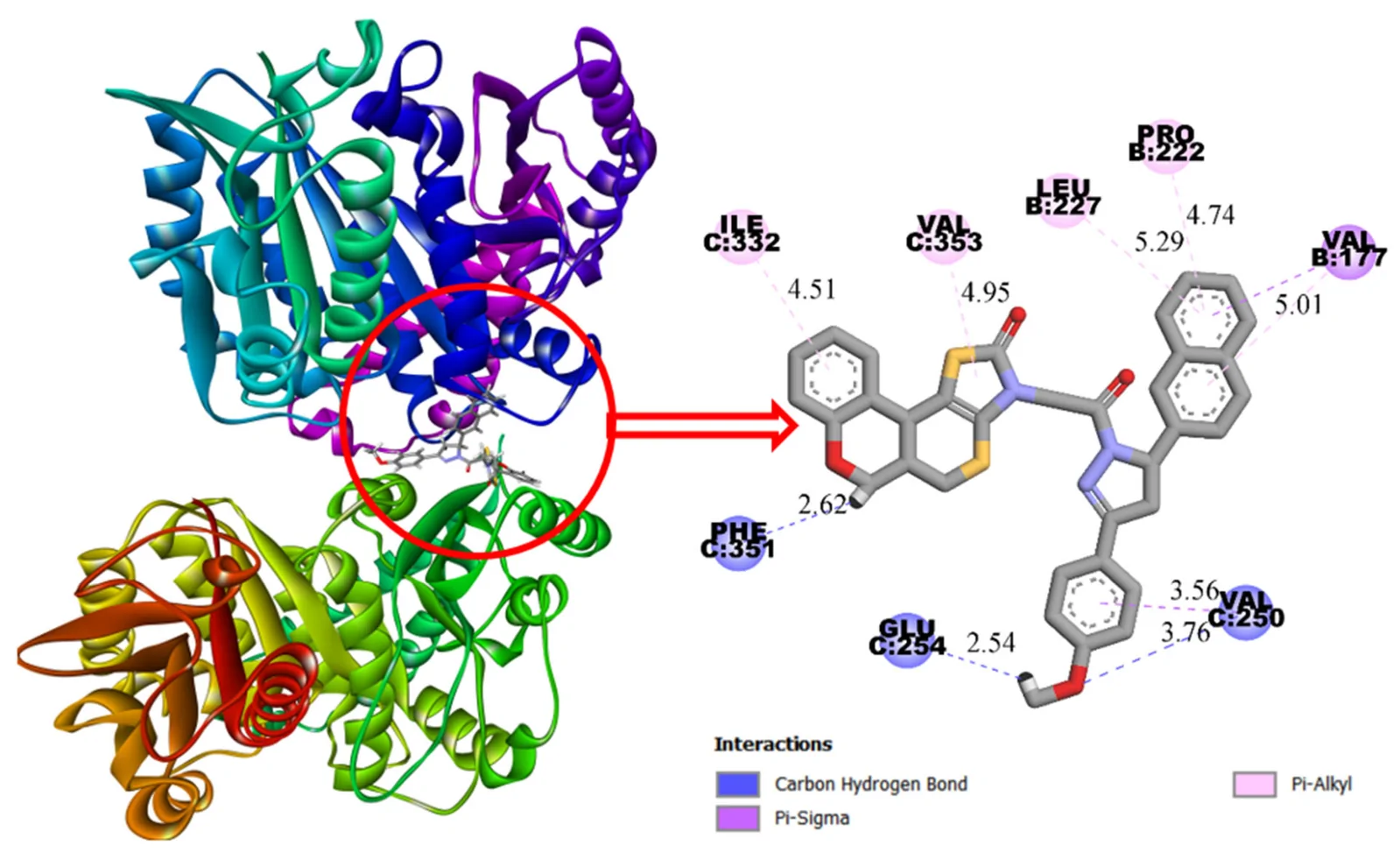
3D & 2D schemes of the Les-6370 interaction with tubulin in the vinca binding site (PDB 5J2T).

Additionally, Les-6370 demonstrated the highest binding energy within the vinca binding site. Nevertheless, the compound does not form any hydrogen bonds with the protein; its shape fits well into the lipophilic cavities formed by aliphatic amino acids. The molecule also forms three weak carbon–hydrogen bonds. This pattern is quite similar to that of vinblastine, which also primarily binds through different types of hydrophobic interactions and carbon–hydrogen bonds, and additionally forms only one conventional hydrogen bond with Asn329 (Figure 5).

### 2.2. Molecular Dynamics Analysis of Ligand–Tubulin Complexes at the Colchicine and Taxane Binding Sites

To account for the limited representation of protein flexibility in static docking, we further evaluated complexes of the five top-scoring compounds identified for the colchicine and taxane binding sites using molecular dynamics simulations in GROMACS Analysis of the MD trajectories included root mean square deviation (RMSD), root mean square fluctuation (RMSF), number of hydrogen bonds, and radius of gyration for each complex. RMSD profiles were monitored over a 100 ns simulation period, with average deviations remaining close to 0.3 nm, indicating generally stable complexes.

**Figure 6:**
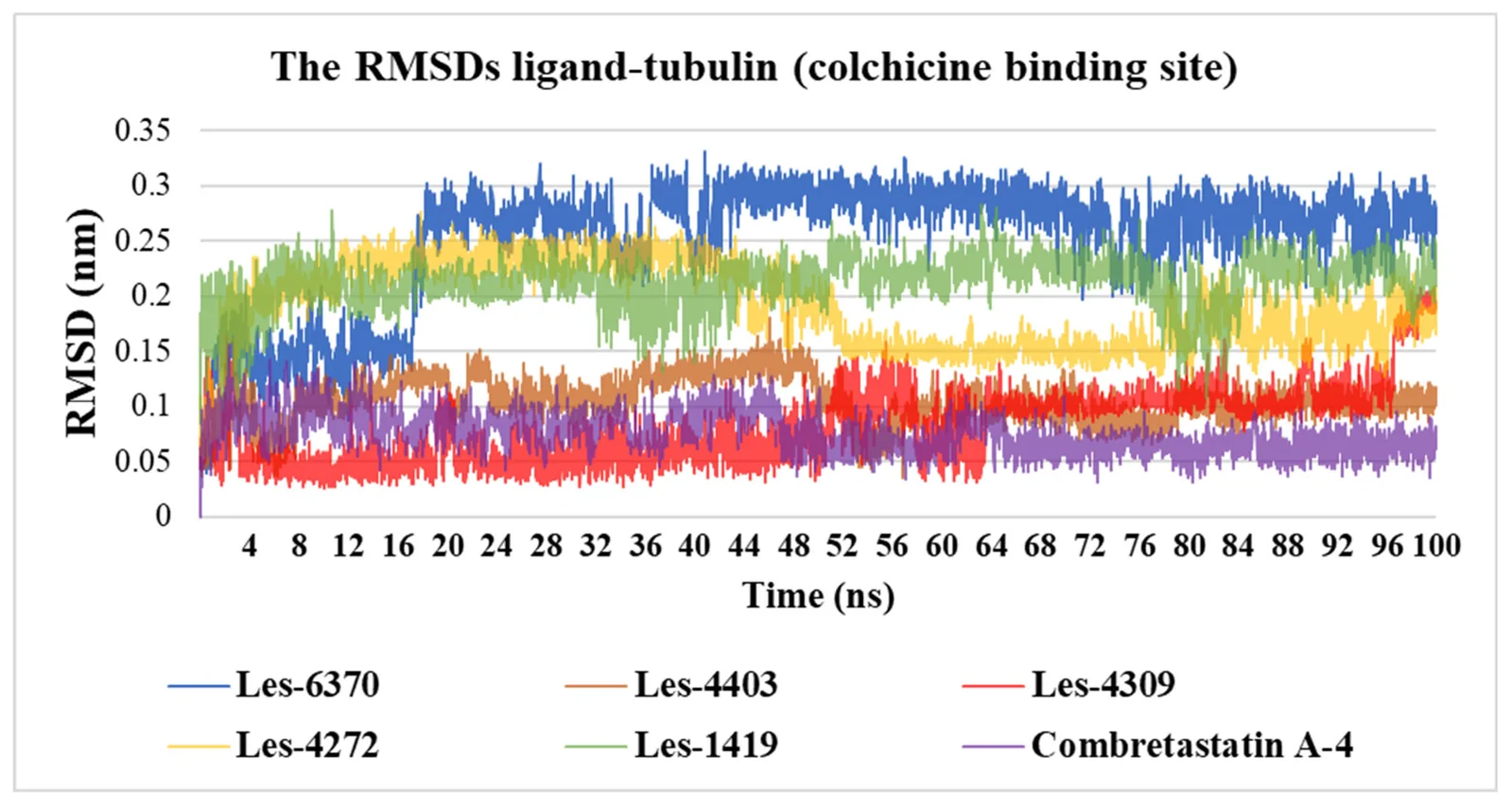
The RMSD profiles of ligand–tubulin complexes at the colchicine-binding site during 100 ns MD simulations. Les-6370—blue, Les-4403—brown, Les-4309—red, Les-4272—yellow, Les-1419—green, Combretastatin A-4—purple.

To assess the stability of ligand–tubulin interactions at the colchicine binding site, RMSD values were calculated over a 100 ns molecular dynamics trajectory for six compounds: Les-6370, Les-4403, Les-4309, Les-4272, Les-1419, and the reference ligand, combretastatin A-4. All compounds exhibited varying RMSD profiles during the simulation, with none exceeding 0.3 nm, indicating no pronounced structural fluctuations during the analyzed trajectory (Figure 6). Notably, combretastatin A-4 exhibited comparatively low RMSD values, consistently remaining within the range of ~0.05–0.10 nm, indicating limited structural deviation from its initial configuration during the analyzed trajectory, consistent with its well-established biological effect on tubulin polymerization. Among the test compounds, Les-4403 (brown) demonstrated a comparable binding mode. Its RMSD values decreased steadily over time and dropped to below 0.1 nm by the end of the 100 ns simulation—approaching the limited structural deviations, which were seen with combretastatin A-4. Furthermore, Les-4403 maintained the highest number of hydrogen bonds throughout the simulation (Figure S5), supporting the formation of a more stable ligand–tubulin complex compared to the other ligands.

The RMSF (Root Mean Square Fluctuation) values for all simulated tubulin–ligand complexes demonstrate a similar pattern of stability, with no significant differences in the fluctuation of amino acid residues, including those within the binding site (Figure S2).

The radius of gyration (Rg) analysis demonstrated no significant change in protein structural stability during binding at the colchicine binding site for all five compounds. A lower Rg value generally indicates greater structural compactness of the system. As evident from Figure S6, the data indicate stabilization and minimal conformational changes in the structure of all complexes.

**Figure 7:**
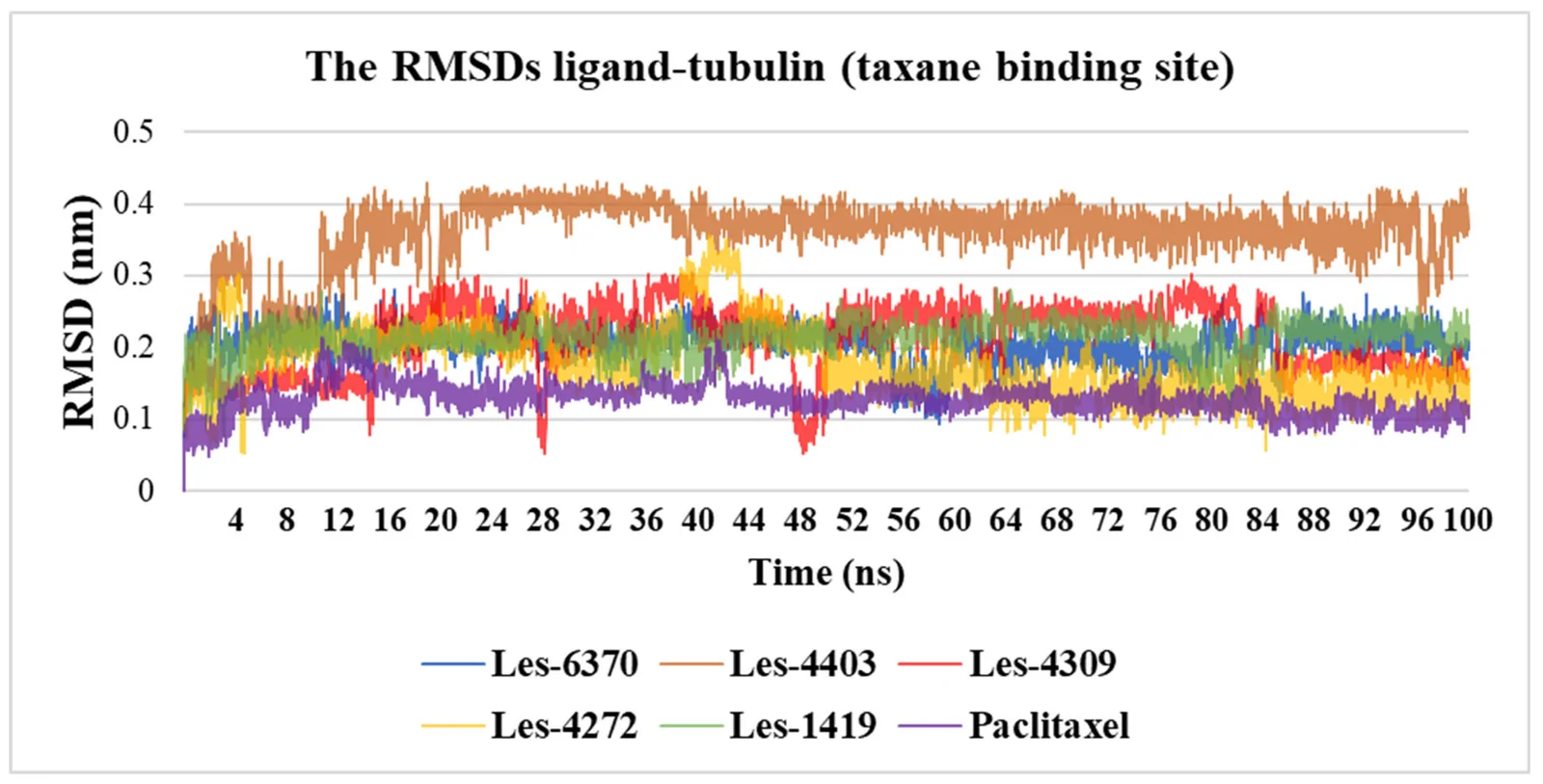
The RMSD profiles of ligand–tubulin complexes at the taxane-binding site during 100 ns MD simulations. Les-6370—blue, Les-4403—brown, Les-4309—red, Les-4272—yellow, Les-1419—green, Paclitaxel—purple.

Examination of the tubulin–ligand complexes at the taxane binding site (Figure 7) revealed that Les-4403 exhibited the largest structural deviations, with RMSD values exceeding 0.3 nm. Les-4309 showed a high level of fluctuation; nevertheless, its RMSD did not exceed 0.3 nm. The most stable complexes were formed by Les-1419 and Les-6370 with tubulin, exhibiting the lowest RMSD values without significant fluctuations. However, Les-6370 does not form persistent hydrogen bond interactions during the simulation, with periods in which no hydrogen bonds are observed. In contrast, Les-1419 maintains a stable number of hydrogen bonds throughout the simulation, with the number increasing up to four.

RMSF analysis showed that most residues of the tubulin complexes remained relatively stable, while higher fluctuations were mainly observed in flexible loop and terminal regions. The increased mobility near the taxane-binding pocket can be associated with the structural flexibility of the β-tubulin M-loop, which includes residues directly involved in paclitaxel binding. Among the tested ligands, Les-1419 produced an RMSF profile most similar to the native ligand, indicating limited disturbance of the local tubulin structure. Overall, these results support a stable accommodation of Les-1419 within the taxane-binding site during the simulation (Figure S4).

The average number of hydrogen bonds formed between the ligands Les-6370, Les-4403, Les-4309, Les-4272, Les-1419, and tubulin (PDB ID: 1JFF—taxane binding site) during the 100 ns molecular dynamics simulation is shown in Figure S7. The results indicate that Les-1419 forms the highest number of hydrogen bonds, maintaining up to four throughout the simulation, which may suggest a more stable and persistent interaction with the binding site. Les-4403 and Les-4272 also exhibit moderate hydrogen bonding activity, with values ranging from one to two bonds and occasional peaks up to 3. In contrast, Les-6370 and Les-4309 form fewer hydrogen bonds, typically fluctuating around one or less, indicating weaker or more transient interactions. These observations confirm that hydrogen bonding contributes significantly to the binding stability of certain ligands, particularly Les-1419, which may enhance its overall affinity toward tubulin in the taxane site.

The Rg values for the Les-6370, Les-4403, Les-4309, Les-4272, and Les-1419 complexes in this MD simulation remained within a narrow range of approximately 2.12 to 2.18 nm. Minor fluctuations were observed, particularly for Les-4403 and Les-4272, but these remained within acceptable limits and did not indicate any notable loss of structural compactness.

Molecular dynamics simulations of both selected binding sites reveal that the studied compounds form significantly more stable complexes within the colchicine binding site, characterized by a lower RMSD value and an increased number of hydrogen bonds. Conversely, the complexes within the taxane binding site generally exhibit lower stability, except for the Les-1419-tubulin complex. This finding suggests a strong interaction between the tested compound and tubulin complex within the taxane binding site.

### 2.3. In Silico ADMET Profile and Drug-likeness Evaluation of the Selected Virtual Hits Les-1419, Les-4272, Les-4309, Les-4403, and Les-6370

ADMET and drug-like properties of the selected virtual hits Les-1419, Les-4272, Les-4309, Les-4403, and Les-6370 were calculated to predict the pharmacokinetic and toxicity potential of the selected virtual hits. Standard criteria, including Lipinski’s “Rule of Five” and Veber’s rules, were applied during screening of the top docking hits. Drug-likeness predictions were carried out using the SwissADME (http://www.swissadme.ch/ accessed on 20 May 2026). and ProTox 3.0 (https://tox.charite.de/protox3/ accessed on 22 May 2026) web servers, with the results summarized in Table 4.

According to SwissADME, Les-6370 and Les-4403 showed possible limitations regarding compliance with Lipinski’s Rule of Five, mainly due to their high molecular weight and/or lipophilicity. These limitations should be considered and addressed during future optimization of new agents that may affect tubulin dynamics, based on their scaffolds. The predicted LD_50_ values ranged from 500 to 1700 mg/kg, with all compounds assigned to toxicity class 4. However, experimental studies are required to confirm their pharmacokinetic and toxicity profiles.

Based on the generated results, compounds Les-6370 and Les-4403 exhibit issues with lipophilicity and molecular weight, resulting in violations of the modified Lipinski rules. Additionally, these two compounds demonstrate lower solubility compared to the other three. Notably, Les-6370 exhibits the most adverse toxicity profile. On the other hand, Les-4309, Les-4272, and Les-1419, which scored highest in docking, demonstrate a satisfactory level of drug-likeness and do not exhibit significant violations in their ADMET profiles.

### 2.4. Applied Synthetic Approaches and Characterization of the Selected Virtual Hits Les-1419, Les-4272, Les-4309, Les-4403, and Les-6370

**Scheme 1:**
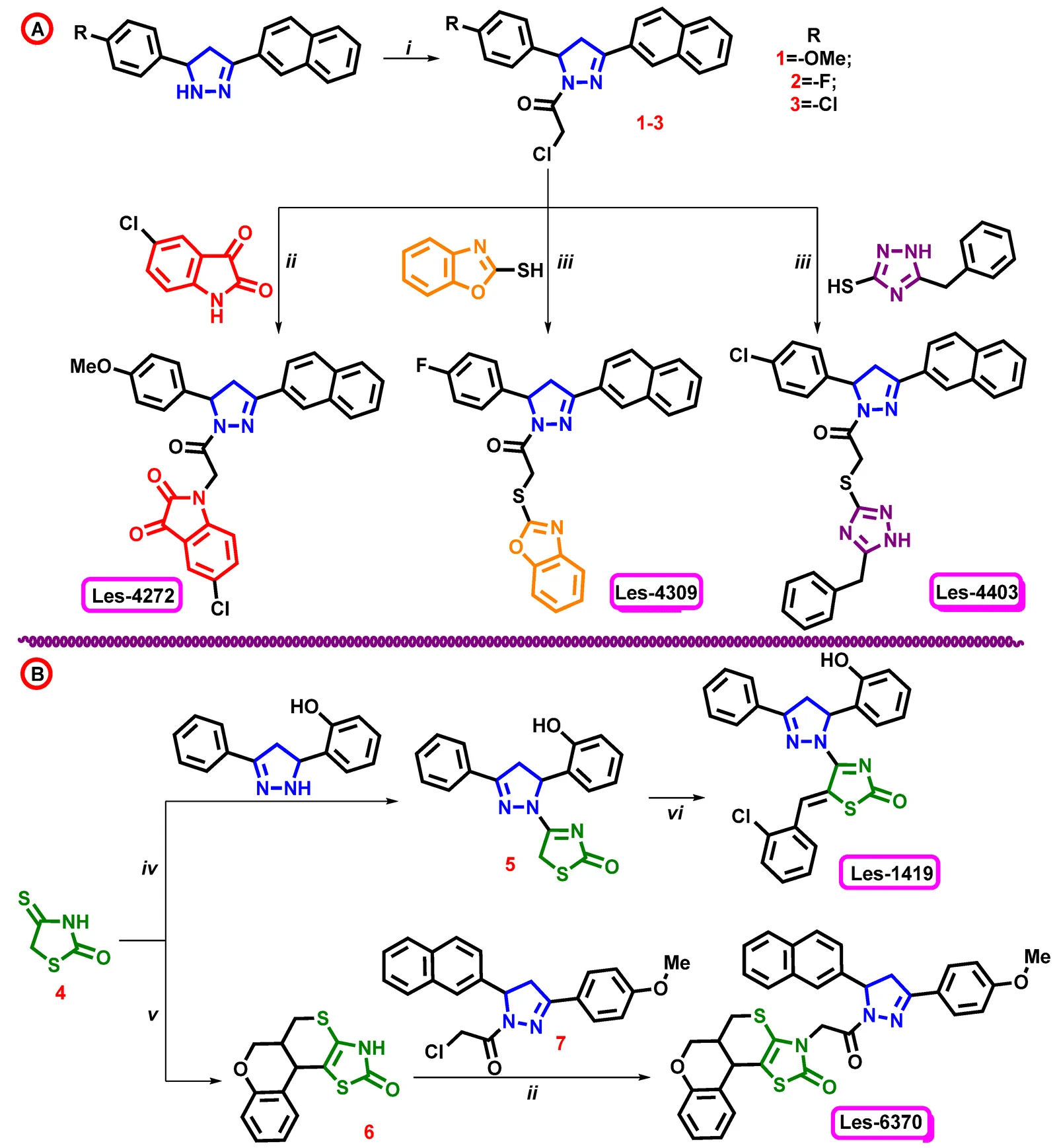
Synthetic routes to the pyrazoline-bearing hybrids (**A**) Les-4272, Les-4309, Les-4403, (**B**) Les-1419, and Les-6370 as potential tubulin-targeting agents. Reagents and conditions: (i) appropriate 3,5-disubstituted-4,5-dihydro-1H-pyrazoles (10 mmol), chloroacethyl chloride (10 mmol), triethylamine (10 mmol), dioxane (10 mL), stirring, r.t. 3 h; (ii) 1 (10 mmol) with 5-chloroindoline-2,3-dione (10 mmol) or 7 (10 mmol) with 6 (10 mmol), K_2_CO_3_ anhydrous (10 mmol), DMF (10 mL), stirring, r.t. 3 h; (iii) 2 or 3 (10 mmol), benzo[d]oxazole-2-thiol or 5-benzyl-4H-1,2,4-triazole-3-thiol (10 mmol), triethylamine (10 mmol), ethanol (10 mL), reflux, 3 h; (iv) 4 (10 mmol), 2-(3-phenyl-4,5-dihydro-1H-pyrazol-5-yl)phenol (10 mmol), ethanol (10 mL), reflux, 1 h; (v) 4 (10 mmol), 2-(allyloxy)benzaldehyde (10 mmol), triethylamine (10 mmol), glacial acetic acid (10 mL), stirring, r.t. 12 h; (vi) 5 (10 mmol), 2-chlorobenzaldehyde (10 mmol), CH_3_COONa anhydrous (10 mmol), glacial acetic acid (10 mL), reflux, 2 h.

Among the identified hits, derivatives Les-4272, Les-4309, Les-4403, and Les-6370 have not been described earlier. Retrosynthetic analysis of these molecules revealed the presence of a pyrazoline moiety. This structural feature served as the basis for the synthetic strategy, which used pyrazoline-containing building blocks to prepare the target compounds (Scheme 1). Corresponding pyrazoline-based chloroacetamides 1–3 were first obtained from appropriate 5-aryl-3-phenyl-2-pyrazolines for the synthesis of Les-4272, Les-4309, and Les-4403 (Scheme 1A). In the subsequent step, these intermediates were employed as alkylating agents in reactions with 5-chloroisatin, 2-mercaptobenzoxazole, and 5-benzyl-4H-1,2,4-triazole-3-thiol to afford the target products.

The synthesis of Les-1419 was previously reported in the literature [20] and the synthetic pathway starting from isorhodanine 4 is outlined in Scheme 1B. Intermediate 5 was obtained from isorhodanine 4 according to the reported protocol [21] and subsequently used for the synthesis of the thiopyrano [2,3-d]thiazole derivative Les-6370 via alkylation with chloroacetamide 1.

The structures of derivatives Les-4272, Les-4309, Les-4403, and Les-6370 were confirmed using ^1^H and ^13^C NMR and LC-MS spectra (copies of spectra are presented in the Figures S8–S19). LC-MS spectra displayed molecular ion peaks at the corresponding *m*/*z* values in the positive ionization mode, confirming the formation of the reported compounds. In the ^1^H NMR spectra of derivatives Les-4272, Les-4309, Les-4403, and Les-6370, diastereotopic pyrazoline nucleus protons form an ABX spin system, appearing as a set of signals at 5.00–5.60, 3.95–4.00 ppm, and 3.20–3.35 ppm with characteristic spin-spin coupling constants. Protons of the thiopyrano [2,3-d]thiazole scaffold of compound Les-6370 resonate with a characteristic pattern as reported in Matiychuk et al., 2008 [21]. Also, ^1^H NMR spectra of the molecules Les-4272, Les-4309, Les-4403, and Les-6370 exhibit signals corresponding to both aliphatic and aromatic protons in the expected spectral regions, with coupling patterns and spin–spin coupling constants consistent with the proposed molecular structures.

### 2.5. In Vitro Cytotoxicity Evaluation of Les-1419, Les-4272, Les-4309, Les-4403, and Les-6370

Among the synthesized derivatives, the highest cytotoxic activity on a wide range of cell lines was demonstrated by the compound Les-1419, which exhibits low IC_50_ values (4.3–18.3 μM) on breast cancer cells (MCF-7, MDA-MB-231), colorectal cancer (HT-29, DLD-1), gastric (AGS), and leukemia (Jurkat, RPMI-8866, K562) after 48–72 h of incubation (Table 5). Jurkat cells demonstrated the highest sensitivity, consistent with their well-documented high responsiveness to classical microtubule-destabilizing agents, such as vincristine [22]. However, Les-1419 also showed cytotoxicity towards non-cancerous cells (HaCaT, BALB 3T3, HEK-293) (Table 6).

Les-4403 showed selective toxicity towards RPMI-8866 cells (IC_50_ 5.4 µM, 72 h), which makes it a promising candidate for further study in the context of acute leukemias (Table 5). In contrast, Les-1419 and Les-4309 were active on Jurkat cells after 48 h of treatment (IC_50_ 4.9 and 3.6 μM, respectively), making them the most promising compounds among those analyzed. Les-6370 was inactive (IC_50_ > 20 μM) on most cell lines. Doxorubicin was used as a positive control, and it demonstrated higher cytotoxicity compared to the tested compounds. Graphical representations of the MTT assay results after 24, 48 and 72 h of treatment are provided in Figures S20–S23.

The selectivity index (SI) was calculated to assess the compound’s ability to target cancer cells while sparing normal cells preferentially. These values exceed the commonly used threshold of SI > 3 for selective cytotoxicity [23,24] although they represent minimum estimates. The differences observed for Les-1419 (SI = 1.8 for MCF-7 vs. 3.1 for MDA-MB-231) arise mainly from the different sensitivities of these two breast cancer lines, which differ in receptor status, p53 status, and phenotype [25]. Compound Les-1419 with SI ≥ 3 was considered moderately selective for MDA-MB-231 cells. Compounds Les-4309 and Les-4272, with SI values of 8.7 and 10.0, were considered selective for Jurkat cells. Compound Les-4403 possessed an SI value of 9.3, indicating its selectivity for RPMI-8866 cells (Table 7). The higher sensitivity of Jurkat cells to Les-4309 may reflect intrinsic features of this line. Jurkat cells carry a PTEN mutation that leads to constitutive phosphorylation and activation of PKB/Akt [26] and lymphoid leukemia lines have been reported to be more sensitive to pro-apoptotic agents than myeloid leukemia lines in at least one comparative study [27]. These lineage-specific features, together with possible differences in compound uptake and structure–activity relationships within the series of studied compounds, may explain the preferential activity of Les-4309 toward Jurkat cells; direct target-engagement studies are needed to confirm this.

The selectivity profiles reported here should be seen as an early filter for compound prioritization, not as evidence of a therapeutic window. In vitro SI does not account for pharmacokinetics, tissue distribution, metabolism, or toxicity in tissues not represented by the normal cell line used. Compounds with SI > 3 were considered selective. The most encouraging profile, that of Les-4309 and Les-4272 in Jurkat cells (SI values of 8.7 and 10.0), supports further evaluation but requires confirmation in a wider panel of non-malignant cells.

Based on the results of the MTT cytotoxicity assays, two compounds, Les-1419 and Les-4309 were selected for further studies of their antiproliferative activity. Les-1419 demonstrated the highest overall cytotoxicity and Les-4309 showed marked sensitivity in Jurkat cells. These compounds were further evaluated for their ability to inhibit tubulin in leukemia models.

### 2.6. β-Tubulin Concentration Assessment Using ELISA

**Figure 8:**
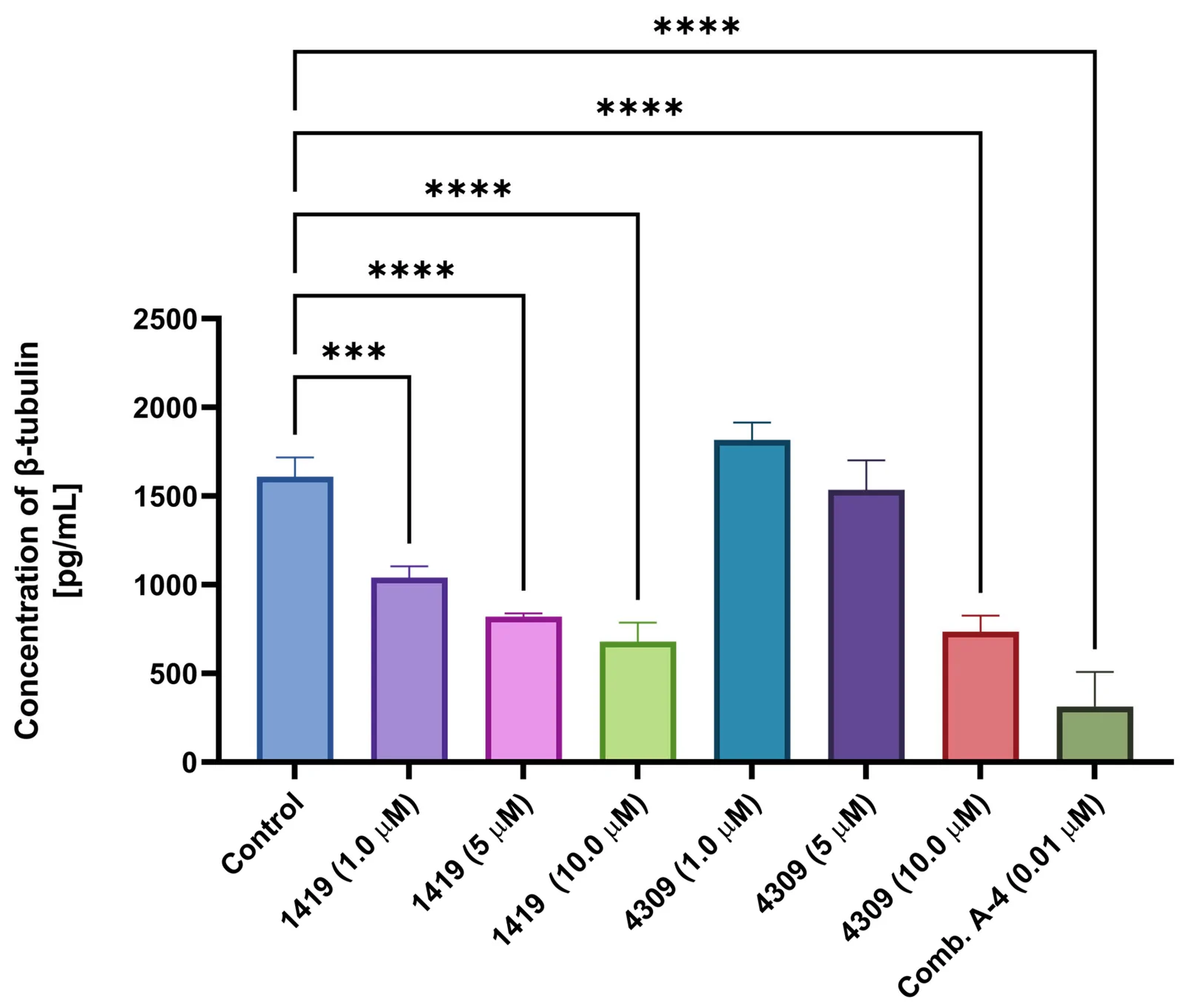
Graphical representation of β-tubulin concentration assessment using ELISA for compounds Les-1419, Les-4309, and Combretastatin A-4 as a positive control in Jurkat cells after 48 h of treatment. One-way ANOVA analysis was applied with Dunnett’s test to adjust for multiple comparisons and showcase statistical significance. *N* = 3; *** *p* ≤ 0.001; **** *p* ≤ 0.0001.

To analyze the tested compounds’ capabilities to inhibit β-tubulin, an efficient and selective method for quantitative measurement—human β-tubulin ELISA kit was applied. For this analysis, we tested structures with the most outstanding results in previous analysis, namely Les-1419 and Les-4309. Compounds were tested in concentrations equal to 1 µM, 5 µM, and 10 µM after 48 h of treatment on Jurkat cells. This cell line was chosen taking into consideration the previously obtained MTT results, which showcased those novel structures had the most significant cytotoxic activity on this particular cell line. Chosen concentrations were utilized due to the difference in IC_50_ results between the compounds and to showcase dose-dependent effects of novel structures on β-tubulin inhibition. Combretastatin A-4 was chosen as a positive control, since it is a well-known and potent β-tubulin inhibitor. Tubulin proteins play a crucial part in the process of microtubule assembly and mitosis of the cells, which are often deregulated in cancer cells [28]. As such, to assess the compounds’ effects on β-tubulin inhibition, it is essential to observe their impact after the cells have gone through at least one complete doubling time. In the case of Jurkat cells, the doubling time is 20.7 ± 2.2 h [29]. Consequently, it was decided that 48 h of treatment would be the optimal time for all cells to go through at least one mitotic event, with the addition of a sufficient amount of time for the effects to become observable. The obtained results clearly displayed that both compounds have a potent inhibitory effect on β-tubulin protein after 48 h of treatment on Jurkat cells (Figure 8). However, Les-1419 showcased a more notable dose–response inhibitory effect with statistically significant results for each tested concentration. For instance, at concentration of 10 µM Les-1419 reduced the concentration of β-tubulin by 57.73% (679.7 ± 105.6 pg/mL) in comparison to the control (1608.0 ± 109.9 pg/mL). In addition to that, Les-1419 also displayed a statistically significant inhibition of the tested protein at lower concentrations: at 5 µM reduced concentration of β-tubulin was 48.94% (821.0 ± 17.06 pg/mL), while concentration equal to 1 µM reduced it by 35.32% (1040.0 ± 64.13 pg/mL) in comparison to the control. In contrast, Les-4309, despite having a lower IC_50_ value, showcased a statistically significant effect only at its highest tested concentration equal to 10 µM with a 54.31% (734.7 ± 91.78 pg/mL) reduction in comparison to the control (Table S3). Such results may suggest that Les-4309, a complex structure with numerous heteroatomic fragments, can have another mechanism of anticancer activity in addition to β-tubulin inhibition. Each of the structural fragments, present in Les-4309, is known for a wide range on potential mechanisms of anticancer activity, unrelated to the tubulin inhibition. For instance, compounds with pyrazoline core previously showcased their anticancer activity via inhibition of various kinases (EGFR, VEGFR-2, HER2, BRAF^V600E^), inhibition of topoisomerase II, carbonic anhydrases IX and XII, telomerase, heat shock protein 90 or aromatase [30]. Compounds with a benzoxazole structural fragment also possess a diverse range of potential mechanisms of anticancer activity, including inhibition of topoisomerase I and II [31], carbonic anhydrases IX and XII [32], VEGFR-2 [33] and COX-2 [34]. Taking into consideration the selectivity of Les-4309 towards Jurkat cells among other tested cell lines, some of the mentioned mechanisms of anticancer activity are less likely to be related to the anticancer activity of Les-4309. Inhibition of carbonic anhydrases IX and XII is often associated with solid hypoxic tumors rather than suspension cell lines like Jurkat. This cell line also has no or little expression of EGFR, HER2, and VEGFR-2 kinases and is thus less likely to be susceptible to the activity of the compound targeting those molecular targets. A detailed assessment of the anticancer activity of Les-4309 at lower concentrations against Jurkat cells is required before conclusions can be drawn about additional mechanisms of its anticancer activity.

Notably, among the two derivatives evaluated in the tubulin-related assay, the more potent compound exhibited improved stabilization within the predicted colchicine-binding site during molecular dynamics simulations. This observation further supports the proposed mechanism involving interference with tubulin dynamics. However, ELISA assessment of β-tubulin concentration cannot directly confirm the compound’s ability to inhibit β-tubulin polymerization in Jurkat cells. More complex methods (tubulin polymerization assay, fluorescence microscopy of microtubule network, and cryo-EM or X-ray crystallography of the compound-tubulin complex) will have to be applied in future research to analyze the full extent of Les-1419 and Les-4309’s ability to inhibit tubulin polymerization. Another limitation of the presented results is the lack of in vivo assessment of the compounds’ anticancer activity and safety. The utilized in vitro biological models cannot fully encompass the complexity of the whole organism, as they do not possess the pharmacokinetic and pharmacodynamic properties of such models. Herein, the promising data obtained regarding the compounds’ activity against β-tubulin cannot be confidently translated to therapeutic efficacy in vivo without performing experiments with appropriate in vivo models.

### 2.7. Assessment of Structural Novelty and Preliminary Structure–Activity Relationship Analysis (SAR)

Structural novelty of the most promising compounds Les-1419 and Les-4309 was examined using Reaxys and the SwissTargetPrediction platform based on their SMILES strings. No literature reports linking these structures to tubulin inhibition were identified in Reaxys. SwissTargetPrediction, which estimates potential protein targets through 2D and 3D similarity comparisons with bioactive molecules annotated in ChEMBL, did not indicate significant similarity of Les-1419 and Les-4309 to known tubulin ligands. The predicted probability values for alternative targets were low. However, it is important to consider a well-known “blind spot” of such ligand-similarity tools: their algorithms are inherently biased toward well-explored areas of chemical space. For truly novel or underrepresented chemotypes, a lack of high-scoring target predictions often reflects the program’s reliance on established reference sets rather than an actual absence of biological affinity. Therefore, these negative similarity results should be interpreted with caution. They do not serve as absolute proof of structural novelty, but rather indicate that the studied derivatives possess distinct features that diverge from classical, well-documented tubulin-targeting chemotypes. The detailed prediction results are summarized in Table S1.

**Figure 9:**
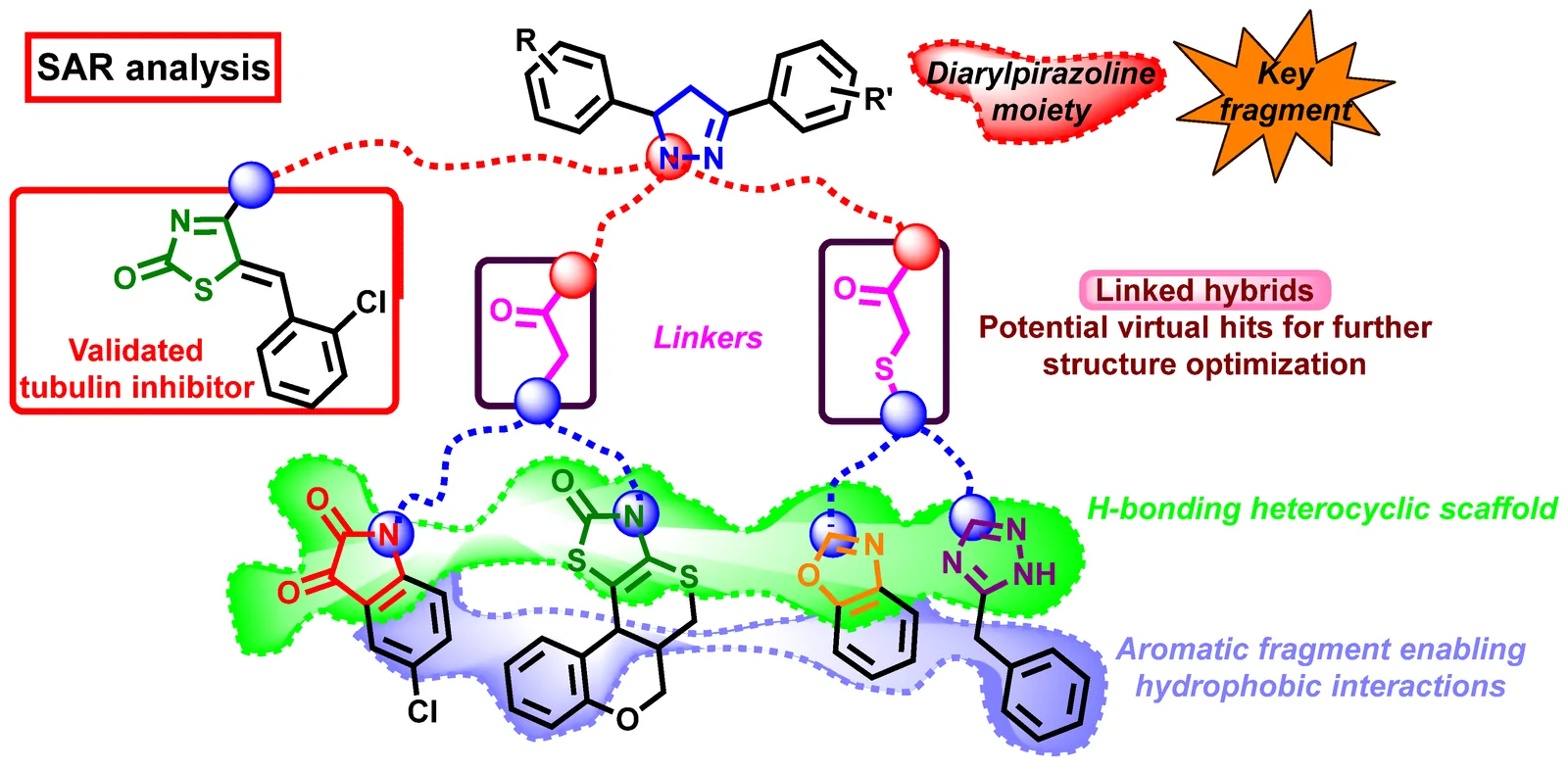
Proposed structure–activity relationship hypotheses for the identified hit compounds as potential tubulin-targeting agents.

Overall, although the structural diversity of the current series precludes the establishment of a definitive classical SAR, the obtained data allow the formulation of several working hypotheses that could be treated as an initial proposed model (Figure 9). The diarylpyrazoline scaffold emerged as a recurring structural motif with strictly 13 out of the 18 virtual hits belonging to this chemotype, suggesting a possible relationship between the diarylpyrazoline framework and the calculated affinity values. Five of these derivatives exhibited varying levels of anticancer activity, and subsequent in vitro evaluation led to the identification of one diarylpyrazoline-containing molecule as a potential hit compound. However, such findings highlight the need for more extensive screening to validate these trends fully.

While multifunctional diarylpyrazolines are established tubulin-targeting scaffolds [35,36,37,38,39] the present series represents a novel chemotype, featuring unique structural characteristics that warrant further SAR studies and hit optimization. Validated hit Les-1419 represents a novel linked hybrid where the diarylpyrazoline core is directly attached to the C4 position of the thiazolidine ring. This structural feature distinguishes it from previously reported bioisosteres [36], where the diarylpyrazoline scaffold was linked to the C2 position of the thiazolidone core. The remaining hits within this series are molecular hybrids where the diarylpyrazoline moieties are linked with various hydrogen-bonding nitrogen-, sulfur-, and oxygen-containing heterocycles via specific spacers. In Les-4272 and Les-6370, a methylenecarbonyl linker is utilized, while in Les-4403 and Les-4309, a thiomethylenecarbonyl bridge is used, in contrast to the reported hybrids in [37,38,40], where a shorter and structurally simpler carbonyl linker was used. Furthermore, while the strategies in [38,39] focus on the hybridization of diarylpyrazolines with the classical 3,4,5-trimethoxyphenyl tubulin pharmacophore, the current series explores alternative chemical space. In particular, the unique linked-hybrid Les-6370, which incorporates a condensed chromeno [4′,3′:4,5]thiopyrano [2,3-d]thiazole framework, can be considered a distant bioisosteric analog of the coumarin-bearing hybrids described in [38]. These positional modifications, elongated linkers, and complex heterocyclic combinations could serve as a rational basis for the design of novel tubulin-targeted agents and guide further hit optimization.

## 3. Materials and Methods

### 3.1. DTP NCI-60 Cell Lines Anticancer Screening In Vitro

The anticancer assay was performed on a panel of approximately sixty human tumor cell lines derived from nine neoplastic diseases, in accordance with the protocol of the Drug Evaluation Branch, National Cancer Institute (Bethesda, MD, USA)and in accordance with a previously described protocol [40,41,42,43].

### 3.2. Molecular Docking

**Figure 10:**
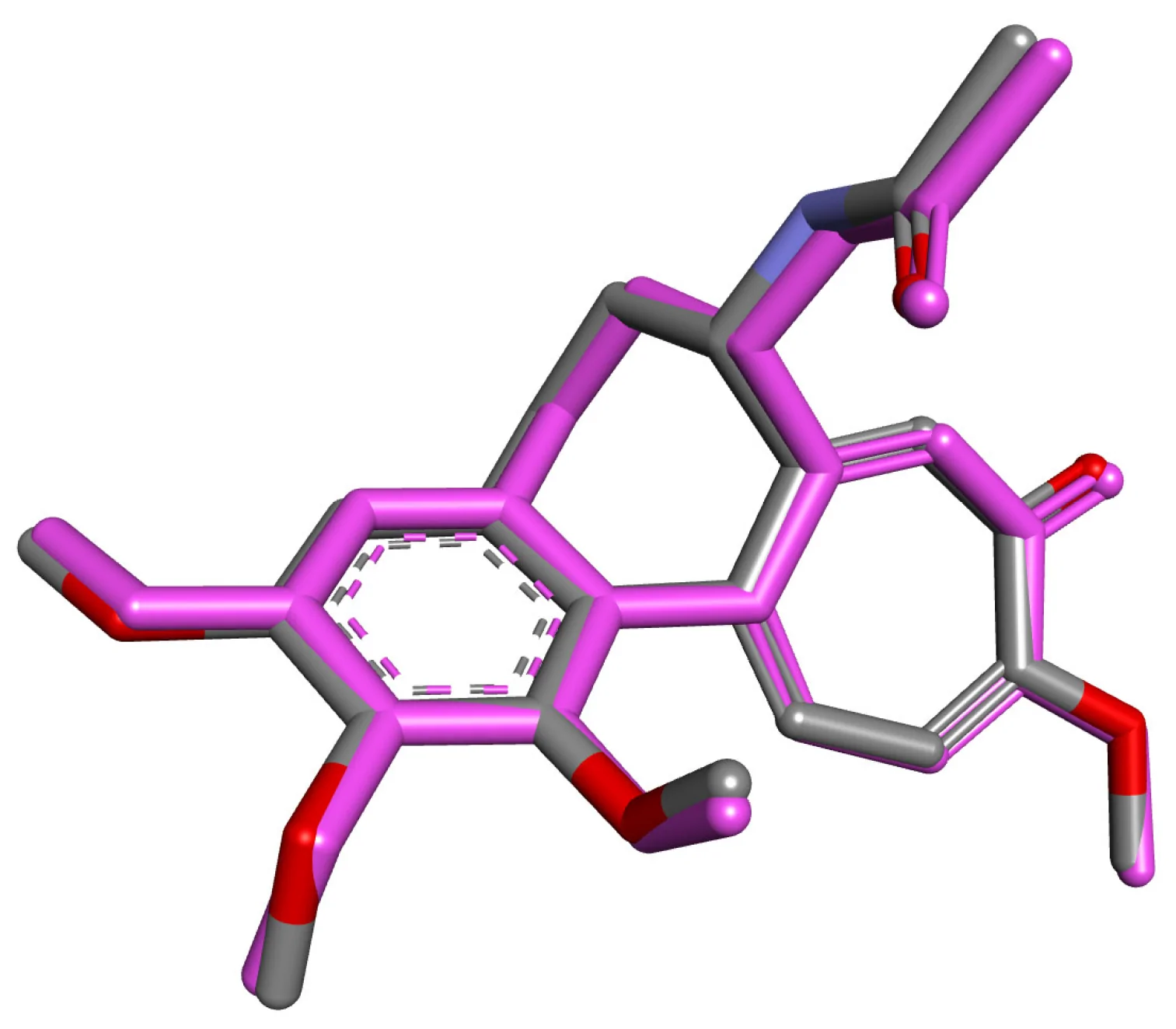
Superposition of the experimentally determined (grey) and predicted docked (pink) poses of colchicine within the colchicine-binding site of tubulin. The RMSD between the experimental and re-docked poses was 0.645 Å.

The 260 most potent compounds from the in-house library with in vitro experimentally confirmed anticancer activity were selected and evaluated in silico for their ability to inhibit tubulin polymerization. Three tubulin binding sites frequently targeted by anticancer agents and novel compounds were selected for investigation: the colchicine, vinca, and taxane sites [44,45]. Docking simulations were conducted using AutoDock Vina 1.1.2, due to its proven accuracy in reproducing native inhibitor binding poses and quick computation [46]. Corresponding protein structures from the PDB database (https://www.rcsb.org/, 11 September 2026) were employed: PDB 4O2B for the colchicine binding site (the complete α/β heterodimer was retained for docking) [47], PDB 5J2T for the vinca site [48], and PDB 1JFF for the taxane site [49]. For the colchicine-binding site in 4O2B, the grid was centered at x = 16.245, y = 64.892, and z = 42.059 Å. For the taxane-binding site in 1JFF, the grid center was defined as x = 0.000, y = −16.102, and z = 15.475 Å. For the vinca-binding site in 5J2T, the grid was centered at x = 0.000, y = 43.067, and z = 17.155 Å. Before the simulation, the structures of all tested compounds were protonated at pH 7.4 and energy-minimized in Avogadro using the MMFF94 force field for 10,000 cycles [50,51]. All obtained structures were verified manually after the optimization procedures. Simulation parameters were optimized to improve reliability: exhaustiveness was increased from 8 to 32, while num_modes and energy_range were set to 10 and 4, respectively [52]. Proteins were prepared in AutoDock Tools 1.5.6 by removing water molecules and ligands, adding polar hydrogens, and assigning Kollman charges [53]. A cubic grid box, centered on each binding site, was defined with dimensions of 22.5 × 22.5 × 22.5 Å. Validation of the docking protocol was achieved by re-docking native ligands and comparing predicted poses with experimental structures. The resulting RMSD values were 0.645 Å for the native ligand at the colchicine-binding site, 1.498 Å for paclitaxel at the taxane-binding site, and 0.973 Å for vinblastine at the vinca-binding site (Figure 10). Visual inspection confirmed that the re-docked poses reproduced the key interactions of the native ligands observed in the crystallographic complexes.

The RMSD values from the re-docking procedure were below 2 Å, indicating that the generated docking models were valid. Binding energies were assessed relative to the energies of the corresponding native ligands: Colchicine and Combretastatin A-4 for the colchicine site, and Vinblastine and Paclitaxel for the vinca and taxane sites, respectively.

### 3.3. Molecular Dynamics

To evaluate the stability of the most promising compounds in complex with tubulin, molecular dynamics (MD) simulations were performed using GROMACS (https://www.gromacs.org/, 13 September 2026) [54] via the SiBioLead server (https://sibiolead.com/, 10 September 2026). Each tubulin–ligand complex was placed in a triclinic simulation box with average dimensions of 8.563 × 11.642 × 11.015 nm after pressure equilibration, solvated with water, and neutralized with Na^+^ and Cl^−^ ions at a final NaCl concentration of 0.15 M. The protein was parameterized using the OPLS-AA force field. Ligand topologies and force-field parameters were generated using the automated workflow implemented in the SiBioLead server. The ligands were protonated and parameterized within the General AMBER Force Field framework (GAFF/GAFF2) [55]. Partial atomic charges were assigned using the AM1-BCC method implemented in the Antechamber suite, while topology generation and conversion were managed by ACPYPE (https://bio2byte.be/acpype/, 9 September 2026) [56]. Before production runs, the system was equilibrated using NVT and NPT ensembles for 1000 ps. A single MD simulation was then carried out for 100 ns employing the leap-frog integrator for each protein–ligand complex. Trajectory frames were recorded every 20 ps, yielding a total of 5000 frames. The resulting data were analyzed using the built-in trajectory analysis tools. The analysis was performed based on four main parameters: ligand RMSD relative to the protein after protein backbone fitting, root-mean-square fluctuation (RMSF), the number of protein–ligand hydrogen bonds, and the radius of gyration (Rg). RMSD and Rg values are presented as time-dependent profiles. Mean ± SD values calculated across trajectory frames were used only to describe within-trajectory fluctuations and should not be interpreted as estimates of variability between independent simulations.

### 3.4. ADMET Profile

The absorption, distribution, metabolism, excretion, and toxicity (ADMET) properties of the selected compounds were evaluated to assess their drug-likeness. Predictions were performed for the top docking hits using the web-based tools SwissADME [57] and ProTox 3.0 [58].

To substantiate the structural novelty claims, an in silico investigation was conducted in May 2026 using the Reaxys database (https://www.reaxys.com, 13 May 2026) and the SwissTargetPrediction platform (https://www.swisstargetprediction.ch/, 12 May 2026). The calculations and database queries were performed based on the canonical SMILES representations of the target virtual hits (Table S2). In Reaxys, exact structure and substructure searches were executed to screen for any prior literature reporting these specific scaffolds or linking them to tubulin polymerization inhibition. In SwissTargetPrediction, potential protein targets were estimated through 2D and 3D similarity comparisons with bioactive molecules from the ChEMBL database (https://www.ebi.ac.uk/chembl/, 5 September 2026). The default platform settings were utilized, applying a Tanimoto-based similarity threshold where a probability score of ≥0.11 was required to report any alternative protein target affinity. A probability score of 0 (reported as “none” in Table S1) was observed for tubulin across all evaluated compounds, confirming their structural divergence from characterized, well-documented tubulin-targeting chemotypes.

### 3.5. Chemistry

Melting points were measured in open capillary tubes on a BÜCHI B-545 melting point apparatus (BÜCHI Labortechnik AG, Flawil, Switzerland) and were uncorrected. The elemental analyses (C, H, N) were performed using the Perkin-Elmer 2400 CHN analyzer (PerkinElmer, Waltham, MA, USA) and were within ± 0.4% of the theoretical values. The 500 MHz-^1^H and 126 MHz-^13^C spectra were recorded on a Bruker AVANCE-500 spectrometer (Bruker, Bremen, Germany). All spectra were recorded at room temperature, except where indicated otherwise, and were referenced internally to solvent reference frequencies. Chemical shifts (δ) are quoted in ppm, and coupling constants (J) are reported in Hz. LC–MS spectra were obtained on a Finnigan MAT INCOS-50 (Thermo Finnigan LLC, San Jose, CA, USA). The commercially available solvents and reagents were used without further purification. Synthesis and characterization of compound Les-1419 were reported in [20].

5-Chloro-1-(2-(5-(4-methoxyphenyl)-3-(naphthalen-2-yl)-4,5-dihydro-1H-pyrazol-1-yl)-2-oxoethyl)indoline-2,3-dione (Les-4272)

Yellow powder, yield 84%, mp >250 °C (DMF-EtOH). ^1^H NMR (500 MHz, DMSO-d_6_, δ): 8.28 (s, 1H, arom.), 8.12 (d, J = 9.1 Hz, 1H, arom.), 8.05–7.95 (m, 3H, arom.), 7.69 (s, 1H, arom.), 7.67–7.57 (m, 3H, arom.), 7.19 (t, J = 8.5 Hz, 3H, arom.), 6.88 (d, J = 7.9 Hz, 2H), 5.59 (d, J = 11.4 Hz, 1H, CH), 5.18–5.12 (m, 1H, CH_2_), 5.01 (d, J = 16.4 Hz, 1H, CH_2_), 4.03–3.98 (m, 1H, CH_2_), 3.72 (s, 3H, CH_3_), 3.35 (d, J = 16.6 Hz, 1H, CH_2_). ^13^C NMR (126 MHz, DMSO-d_6_, δ): 194.2 (C=O), 182.4 (C=O), 163.6 (C=O), 159.0, 158.6, 156.6, 137.8, 134.1, 133.1, 128.8, 128.2, 128.1, 128.0, 127.4, 126.5, 124.5, 123.7, 122.0, 114.5, 113.7, 110.6, 105.4, 60.3 (CH), 58.1 (CH_2_), 55.6 (CH_3_), 34.9 (CH_2_). LCMS (ESI+) *m*/*z* 524 (99.0%, [M+H]^+^). Anal. calc. for C_30_H_22_ClN_3_O_4_: C, 68.77%; H, 4.23%; N, 8.02%. Found: C, 68.90%; H, 4.40%; N, 8.20%.

2-(Benzo[d]oxazol-2-ylthio)-1-(5-(4-fluorophenyl)-3-(naphthalen-2-yl)-4,5-dihydro-1H-pyrazol-1-yl)ethan-1-one (Les-4309)

White powder, yield 68%, mp >250 °C (DMF-EtOH). ^1^H NMR (500 MHz, DMSO-d_6_, δ): 8.25 (br.s, 1H, arom.), 8.07 (s, 1H, arom.), 8.03–7.94 (m, 3H, arom.), 7.69–7.55 (m, 4H, arom.), 7.39–7.30 (m, 4H, arom.), 7.12 (d, J = 7.6 Hz, 2H, arom.), 5.69 (dd, J = 11.4, 5.3 Hz, 1H, CH), 4.92 (dd, J = 16.1, 4.3 Hz, 1H, CH_2_), 4.72 (dd, J = 16.4, 4.9 Hz, 1H, CH_2_), 4.06 (dd, J = 18.7, 12.1 Hz, 1H, CH_2_), 3.37 (dd, J = 18.7, 5.3 Hz, 1H, CH_2_). ^13^C NMR (126 MHz, DMSO-d_6_, δ): 163.8 (C=O), 161.3 (d, J = 244.4 Hz), 155.6, 151.3, 141.2, 137.8, 133.7, 132.6, 128.4, 128.3, 128.2, 127.9, 127.7, 127.6, 127.6, 127.4, 126.8, 124.6, 124.2, 123.1, 118.2, 115.3 (d, J = 21.4 Hz), 110.1, 59.5 (CH), 42.1 (CH_2_), 35.4 (CH_2_). LCMS (ESI+) *m*/*z* 482 (100.0%, [M+H]^+^). Anal. calc. for C_28_H_20_FN_3_O_2_S: C, 69.84%; H, 4.19%; N, 8.73%. Found: C, 70.10%; H, 4.40%; N, 8.90%.

2-((5-Benzyl-1H-1,2,4-triazol-3-yl)thio)-1-(5-(4-chlorophenyl)-3-(naphthalen-2-yl)-4,5-dihydro-1H-pyrazol-1-yl)ethan-1-one (Les-4403)

White powder, yield 71%, mp > 250 °C (DMF-EtOH). ^1^H NMR (500 MHz, DMSO-d_6_, δ): 13.75 (s, 1H, triazol), 8.19 (s, 1H, arom.), 8.03–7.95 (m, 5H, arom.), 7.63–7.55 (m, 2H), 7.40–7.35 (m, 3H), 7.32–7.26 (m, 8H), 5.62 (d, J = 11.5 Hz, 1H, CH), 4.56 (d, J = 15.2 Hz, 1H, CH_2_), 4.37 (d, J = 15.1 Hz, 1H, CH_2_), 4.06–3.98 (m, 2H, CH_2_), 3.47 (br.s, 1H, CH_2_), 3.35–3.26 (m, 1H, CH_2_). ^13^C NMR (126 MHz, DMSO-d_6_, δ): 174.3 (C=O), 168.8, 165.3, 158.4, 140.7, 136.5, 133.6, 132.6, 128.9, 128.5, 128.4, 128.3, 128.1, 127.7, 127.4, 126.8, 126.6, 126.4, 123.0, 59.3 (CH), 41.9 (CH_2_), 34.2 (CH_2_), 31.8 (CH_2_). LCMS (ESI+) *m*/*z* 538 (100.0%, [M+H]^+^). Anal. calc. for C_30_H_24_ClN_5_OS: C, 66.97%; H, 4.50%; N, 13.02%. Found: C, 67.10%; H, 4.70%; N, 13.20%.

3-(2-(3-(4-Methoxyphenyl)-5-(naphthalen-2-yl)-4,5-dihydro-1H-pyrazol-1-yl)-2-oxoethyl)-3,5a,6,11b-tetrahydro-2H,5H-chromeno [4′,3′:4,5]thiopyrano [2,3-d]thiazol-2-one (Les-6370)

Yellow powder, yield 73%, mp >250 °C (DMF-EtOH). ^1^H NMR (500 MHz, DMSO-d_6_, δ): 8.09 (t, J = 8.8 Hz, 2H, arom.), 7.97 (br.s, 5H, arom.), 7.58 (br.s, 3H, arom.), 6.90 (br.s, 5H, arom.), 4.92 (br.s, 1H, CH), 4.40 (br.s, 1H, CH_2_), 4.26 (br.s, 1H, CH_2_), 3.98 (br.s, 3H, CH+ CH_2_), 3.72 (br.s, 3H, CH_3_), 3.34 (s, 1H, CH), 3.19 (t, J = 15.1 Hz, 1H, CH_2_), 2.99 (s, 1H, CH_2_), 2.73 (s, 1H, CH_2_), 2.18 (s, 1H, CH_2_). ^13^C NMR (126 MHz, DMSO-d_6_, δ): 172.3 (C=O), 168.3 (C=O), 161.6, 156.1, 139.2, 138.2, 136.2, 133.7, 133.5, 132.8, 132.6, 128.4, 128.3, 127.9, 127.7, 127.4, 126.9, 124.7, 123.8, 123.2, 122.4, 120.6, 117.1, 114.0, 94.6, 68.2 (CH_2_), 59.7 (CH), 56.1 (CH_2_), 55.1 (CH_3_), 41.9 (CH_2_), 33.2 (CH), 27.9 (CH), 18.2 (CH_2_). LCMS (ESI+) *m*/*z* 620 (96.0%, [M+H]^+^). Anal. calc. for C_35_H_29_N_3_O_4_S_2_: C, 67.83%; H, 4.72%; N, 6.78%. Found: C, 68.10%; H, 4.90%; N, 6.90%.

### 3.6. Cell Lines

Human acute T-lymphoblastic leukemia cell line Jurkat, spontaneously immortalized human epidermal keratinocytes HaCaT cell line, human breast adenocarcinoma cell line MCF-7, and human breast adenocarcinoma cell line MDA-MB-231 (triple-negative) were kindly provided by the Cell Collection at the Institute of Molecular Biology and Genetics, National Academy of Sciences of Ukraine (Kyiv, Ukraine). Human colorectal adenocarcinoma cell line HT-29, human gastric adenocarcinoma cell line AGS, human B-lymphoblastoid cell line RPMI-8866 (derived from chronic myelogenous leukemia), and human colorectal adenocarcinoma cell line DLD-1 were kindly provided by Prof. Anna Bielawska (Department of Biotechnology, Faculty of Pharmacy, Medical University of Bialystok, Poland). Murine embryonic fibroblast cell line BALB 3T3 and human embryonic kidney cell line HEK-293 were obtained from the Cell Collection of the R.E. Kavetsky Institute of Experimental Pathology, Oncology and Radiobiology, National Academy of Sciences of Ukraine (Kyiv, Ukraine). BALB 3T3, MCF-7, MDA-MB-231, AGS, HaCaT, DLD-1, K562, and HEK-293 cells were cultivated in DMEM medium (Sigma-Aldrich, USA) containing 2 mM L-glutamine, 4500 mg/L glucose, and 1 mM sodium pyruvate; Jurkat and RPMI-8866 cells were cultivated in RPMI-1640 medium (Sigma-Aldrich, St. Louis, MO, USA). HT-29 cells were cultured in McCoy’s 5A Medium (Sigma-Aldrich, MO, USA). All media were supplemented with 10% fetal bovine serum (Sigma-Aldrich, St. Louis, MO, USA). All studied cell lines were cultured at 37 °C in a humidified atmosphere containing 5% CO_2_.

### 3.7. MTT Assay

Cell viability of tumor and non-cancerous cell lines after incubation with the tested compounds was assessed using the 3-(4,5-dimethylthiazol-2-yl)-2,5-diphenyltetrazolium bromide (MTT reagent) (Sigma-Aldrich, St. Louis, MO, USA). 6000 adherent cells or 15,000 suspension cells per well were seeded into 96-well plates in 100 µL of medium, according to ATCC recommendations, and incubated for 24–96 h at 37 °C in a CO_2_ incubator with varying concentrations of the investigated compounds. After the incubation period, the treatment medium was removed, MTT reagent was added to the cells according to the manufacturer’s instructions (final concentration 5 mg/mL), and the plates were incubated for an additional 4 h. The formazan crystals were dissolved in DMSO, and the absorbance was measured using a BioTek ELx800 Absorbance Reader (BioTek Instruments, Inc., Winooski, VT, USA) at a wavelength of 490 nm. The half-maximal inhibitory concentration (IC_50_) values were calculated using GraphPad Prism 8 software (San Diego, CA, USA) from three independent experiments and expressed as mean (M) ± SEM [59]. The selectivity index (SI) is a quantitative measure used to evaluate the therapeutic window of an anticancer compound and its ability to preferentially inhibit tumor cell growth over non-malignant cells. SI values were determined as the ratio of the mean IC_50_ for human non-malignant cell lines (HaCaT, HEK-293) to the IC_50_ for the studied human tumor cell lines [60]. Where IC_50_ in non-cancerous or tumor cells exceeded the highest tested concentration (>50 µM or >20 µM), the SI was calculated using that concentrations and is a lower-bound estimate, indicated by “≥”. Compounds with SI > 3 were considered selective, in line with the criterion commonly used in cytotoxicity screening [23]. Experiments were conducted in triplicate.

### 3.8. ELISA β-Tubulin Concentration Assessment

Jurkat cells were seeded in 6-well plates, and the tested compounds, Les-1419 and Les-4309, were added in concentrations equal to 1 µM, 5 µM and 10 µM for 48 h of treatment. Afterwards, the cells were collected, centrifuged, and washed with ice-cold phosphate-buffered saline (PBS). Prior to lysis the cells were counted using a Scepter 3.0 handheld automated cell counter (Merck Millipore, Burlington, MA, USA) to ensure equal numbers of cells utilized for the preparation of cell lysates in the lysis buffer. The obtained supernatants were frozen at −80 °C immediately after centrifugation. To perform the Human β-tubulin SimpleStep ELISA Kit (ab245722, Abcam, Cambridge, UK), the standards samples and antibody cocktail were added to the microtiter plate wells for 1 h of incubation at RT. The contents of the microplate wells were extracted, and the wells were washed thrice. Afterwards, a TMB development solution was added to each well. The enzyme–substrate reaction was terminated with a stop solution. The color change was assessed by UV-VIS Helios Gamma (Unicam/ThermoFisher Scientific Inc., Cleveland, OH, USA) at 450 ± 2 nm wavelength. The concentrations of the tested probes were calculated as the interpolated values based on the standard curve of the 8 standard concentrations: 0, 187.50, 375.00, 750.00, 1500.00, 3000.00, 6000.00, 12,000.00 pg/mL in accordance with the protocol. Obtained values in three repetitions of the experiment were utilized for the one-way ANOVA analysis with Dunnett’s test to adjust for multiple comparisons and showcase statistical significance via GraphPad prism Version 6.0 software (San Diego, CA, USA). Statistically significant difference was displayed as *** *p* ≤ 0.001; **** *p* ≤ 0.0001.

## 4. Conclusions

Applying an integrated in silico and in vitro strategy enabled the efficient identification of heterocyclic hit compounds with tubulin inhibitory activity and favorable predicted drug-like properties from a library of approximately 8000 molecules. Within the screened library, the colchicine and taxane binding sites emerged as the most relevant targets, as confirmed by molecular docking and dynamics simulations. Identified hit compounds Les-1419 and Les-4309 exhibited potent β-tubulin inhibition in Jurkat cells, supporting their biological relevance and anticancer potential. The structural novelty of these scaffolds expands the chemical space of tubulin-targeting compounds and offers unique opportunities for rational hit optimization. Collectively, these findings validate the applied approach as a robust tool for hit identification and provide a strong foundation for the design of analogues with enhanced potency, selectivity, and pharmacokinetic profiles. Overall, the study emphasizes both the identified hit compounds and the broader strategy for accelerating the discovery and development of novel anticancer agents targeting microtubule dynamics.

## Figures and Tables

**Table 1:** Docking scores of virtual hits and reference compounds across three distinct binding sites.

Reference Ligands	Colchicine Binding Site (PDB 4O2B) kcal/mol	Taxane Binding Site (PDB 1JFF) kcal/mol	Vinca Site (PDB 5J2T) kcal/mol
Colchicine	—9.9	-	-
Combretastatin A-4	—8.7	-	-
Paclitaxel	-	—9.9	-
Vinblastine	—	-	—11.6
Les-4272	—10.7	—10.7	—10.4
Les-4274	—11.4	—10.5	—9.5
Les-4309	—11.7	—10.0	—11.4
Les-5463	—10.9	—10.2	—11.4
Les-3639	—8.1	—10.0	—10.0
Les-3645	—8.1	—10.3	—10.0
Les-4403	—12.3	—11.1	—9.5
Les-5552	—10.4	—10.3	—9.7
Les-3166	—10.9	—10.2	—8.9
Les-3643	—8.2	—10.5	—10.5
Les-3755	—10.4	—9.9	—11.0
Les-3833	—9.8	—10.2	—9.9
Les-3882	—9.6	—10.9	—10.9
Les-1127	—5.8	—10.1	—10.1
Les-1419	—11.5	—10.6	—10.6
Les-1458	—9.9	—10.0	—10.0
Les-6370	—11.4	—10.1	—11.7
Les-6368	—8.8	—10.4	—10.7

**Table 2:** Intermolecular interactions of tubulin–ligand complexes identified with molecular docking (colchicine binding site).

Compound	Hydrogen Bond (Distance) Å	Lipophilic Interactions (Distance) Å
Les-6370	Ala12 (2.39 Å), Ser140 (2.13 Å), Asn249 (2.81 Å)	Lys254 (3.06 Å), Ala316 (4.85 Å), Ala250 (5.40 Å) Leu248 (4.73 Å), Leu255 (5.22 Å), Lys352 (5.09 Å, 5.46 Å)
Les-4403	Ser178 (2.49 Å)	Cys373 (3.73 Å), Asp329 (3.49 Å), Leu255 (3.68 Å)
Les-4309	Thr353 (2.77 Å), Lys254 (2.29 Å), Asn101 (2.44 Å)	Leu255 (3.95 Å, 3.47 Å), Leu248 (5.17 Å)
Les-4272	Asn249 (2.22 Å), Gln11 (2.21 Å)	Leu255 (3.56 Å, 3.47 Å), Leu248 (3.84 Å)
Les-1419	Lys254 (2.74 Å), Asn101 (2.39 Å), Gln11 (2.23 Å), Glu183 (1.90 Å)	Leu248 (3.65 Å), Asp98 (4.63 Å), Lys254 (2.76 Å)

**Table 3:** Intermolecular interactions of tubulin–ligand complexes identified with molecular docking (taxane binding site).

Compound	Hydrogen Bond (Distance) Å	Lipophilic Interactions (Distance) Å
Les-6370	His229 (2.65 Å)	Arg278 (4.87 Å), Leu371 (5.00 Å, 5.05 Å), His229 (4.87 Å), Leu371 (5.39 Å, 5.22 Å), Val23 (4.47 Å, 3.34 Å, 5.37 Å), Ala233 (4.98 Å), Pro360 (4.75 Å), Phe83 (5.29 Å)
Les-4403	--	Arg284 (3.98 Å), Leu371 (3.69 Å, 3.83 Å), His229 (4.43 Å, 5.39 Å), Leu217 (5.39 Å, 5.22 Å), Phe272 (5.76 Å), Ala233 (4.17 Å, 4.61 Å), Pro360 (4.98 Å, 4.61 Å) Arg369 (5.29 Å)
Les-4309	Gly370 (2.21 Å)	Leu371 (4.83 Å), Arg369 (5.32 Å), Pro360 (3.93 Å, 4.81 Å), His229 (3.57 Å), Leu275 (3.36 Å), Leu239 (5.43 Å), Ala233 (4.81 Å)
Les-4272	--	Leu371 (5.29 Å), Arg278 (4.41 Å, 4.89 Å), Pro360 (5.05 Å, 4.79 Å), Val23 (5.50 Å), His229 (2.76 Å, 4.41 Å, 5.35 Å), Leu217 (3.97 Å), Ala233 (4.47 Å, 4.48 Å), Phe272 (4.83 Å)
Les-1419	Thr276 (2.35 Å), Ser277 (2.74 Å)	Arg284 (4.76 Å), Arg278 (4.09 Å, 4.61 Å), Pro274 (4.98 Å), Leu217 (3.75 Å), Ala233 (4.93 Å), His229 (4.69 Å), Leu230 (4.87 Å)

**Table 4:** ADMET and drug-likeness properties of the best docking score compounds.

ADMET Properties	Les-6370	Les-4403	Les-4309	Les-4272	Les-1419
MW	619.18	538.06	481.54	523.97	459.95
Rotatable bonds	6	8	6	6	4
H-bond acceptors	5	4	5	5	4
H-bond donors	0	1	0	0	1
TPSA, Å^2^	126.67	99.54	84.00	79.28	90.56
MlogP	4.33	5.00	4.69	3.29	4.23
LogS	−7.77	−7.37	−7.03	−6.43	−6.44
Solubility class	Poorly soluble	Poorly soluble	Poorly soluble	Poorly soluble	Poorly soluble
GI absorption	Low	High	High	High	High
BBB permeable	No	No	No	No	No
CYP1A2 inhibitor	No	No	Yes	No	No
CYP2C19 inhibitor	Yes	Yes	Yes	Yes	Yes
CYP2C9 inhibitor	Yes	Yes	Yes	Yes	Yes
CYP2D6 inhibitor	Yes	No	No	No	No
CYP3A4 inhibitor	Yes	Yes	Yes	Yes	Yes
Lipinski rules	No	No	Yes	Yes	Yes
Veber rules	Yes	Yes	Yes	Yes	Yes
Predicted LD_50_, mg/kg	500	1000	1700	1000	800
Predicted Toxicity Class	4	4	4	4	4

**Table 5:** Results of MTT assay of cytotoxicity of compounds Les-1419, Les-4272, Les-4309, Les-4403, and Les-6370 and Doxorubicin (Dox) on tumor cell lines (IC_50_, μM, M ± SEM, *n* = 3).

		Les-1419	Les-4272	Les-4309	Les-4403	Les-6370	Dox
**Breast cancer cell lines**							
**MCF-7**	24 h	17.7 ± 0.9	>20	>20	>20	>20	3.7 ± 0.2
	48 h	9.3 ± 0.5	>20	>20	>20	>20	1.7 ± 0.1
	72 h	7.4 ± 0.5	>20	>20	>20	>20	3.2 ± 0.3
**MDA-** **MB-231**	24 h	10.8 ± 0.7	>20	>20	>20	>20	>20
	48 h	8.0 ± 0.2	>20	>20	>20	>20	>20
	72 h	4.3 ± 0.3	>20	>20	>20	>20	1.8 ± 0.1
**Colorectal cancer cell lines**							
**HT-29**	24 h	15.6 ± 0.4	>20	>20	>20	>20	>20
	48 h	6.3 ± 0.3	>20	>20	>20	>20	>20
	72 h	5.9 ± 0.4	>20	>20	>20	>20	>20
**DLD-1**	24 h	9.3 ± 0.5	>20	>20	>20	>20	>20
	48 h	6.7 ± 0.4	>20	>20	>20	>20	>20
	72 h	8.5 ± 0.3	>20	>20	>20	>20	4.1 ± 0.2
**Gastric cancer cell line**							
**AGS**	24 h	6.4 ± 0.5	18.4 ± 1.7	>20	>20	>20	3.1 ± 0.2
	48 h	5.6 ± 0.3	5.6 ± 0.4	>20	>20	>20	0.1 ± 0.01
	72 h	6.9 ± 0.6	>20	>20	>20	>20	0.5 ± 0.02
**Leukemia cell lines**							
**RPMI-** **8866**	24 h	17.1 ± 0.9	>20	>20	7.1 ± 0.6	>20	>20
	48 h	9.6 ± 0.7	>20	>20	7.9 ± 0.4	>20	>20
	72 h	18.3 ± 1.3	8.7 ± 0.4	>20	5.4 ± 0.3	>20	4.1 ± 0.2
**Jurkat**	24 h	>20	-	6.0 ± 1.7	>20	>20	0.8 ± 0.01
	48 h	4.9 ± 0.6	-	3.6 ± 0.2	>20	>20	0.4 ± 0.04
	72 h	8.0 ± 0.5	5.0 ± 0.4	3.5 ± 0.3	>20	>20	0.1 ± 0.03
	96 h	6.7 ± 0.6	-	-	>20	-	-
**K562**	72 h	>20	>20	>20	>20	>20	>20
	96 h	7.8 ± 0.6	-	-	>20	-	-

**Table 6:** Results of the MTT cytotoxicity assay of compounds Les-1419, Les-4272, Les-4309, Les-4403, and Doxorubicin on non-cancerous cell lines (IC_50_, μM, M ± SEM, *n* = 3).

72 h	Les-1419	Les-4272	Les-4309	Les-4403	Dox
**Non-cancerous cell lines**					
**HaCaT**	5.3 ± 0.3	>50.0	10.7 ± 0.5	>50.0	1.3 ± 0.1
**HEK-293**	21.0 ± 1.1	49.0 ± 1.8	>50.0	>50.0	0.9 ± 0.3
**Balb 3T3**	6.9 ± 0.4	5.51 ± 0.3	>20.0	15.0 ± 1.2	0.8 ± 0.1

**Table 7:** The selectivity index (SI) of compounds Les-1419, Les-4272, Les-4309, Les-4403, and Doxorubicin.

		Les-1419	Les-4272	Les-4309	Les-4403	Dox
**Breast cancer cell lines**						
**MCF-7**	72 h	1.8 ± 0.1	≥2.5	≥1.5	≥2.5	0.3 ± 0.03
**MDA-** **MB-231**	72 h	3.1 ± 0.4	≥2.5	≥1.5	≥2.5	0.6 ± 0.06
**Colorectal cancer cell lines**						
**HT-29**	72 h	2.2 ± 0.1	≥2.5	≥1.5	≥2.5	0.06 ± 0.01
**DLD-1**	72 h	1.6 ± 0.12	≥2.5	≥1.5	≥2.5	0.3 ± 0.03
**Gastric cancer cell line**						
**AGS**	72 h	1.9 ± 0.2	≥2.5	≥1.5	≥2.5	2.2 ± 0.2
**Leukemia cell lines**						
**RPMI-** **8866**	72 h	0.7 ± 0.1	5.8 ± 0.50	>1.52	9.26 ± 0.65	0.27 ± 0.03
**Jurkat**	72 h	1.6 ± 0.1	10.0 ± 0.9	8.7 ± 0.7	>2.5	7.3 ± 0.2
**K562**	72 h	0.7 ± 0.04	≥2.5	>1.5	>2.5	0.06 ± 0.003

SI values were determined as the ratio of IC_50_ for human non-malignant cell lines (HaCaT, HEK-293) to the IC_50_ for the studied human tumor cell lines. Where IC_50_ in non-cancerous or tumor cells exceeded the highest tested concentration (> 50 µM or >20 µM), the SI was calculated using that concentration and is a lower-bound estimate, indicated by “≥”.

## Data Availability

The original contributions presented in this study are included in the article/Supplementary Materials. Further inquiries can be directed to the corresponding authors.

## Supplementary Materials

The following supporting information can be downloaded at https://www.mdpi.com/article/10.3390/ph19101637/s1: Figure S1: 3D & 2D Schemes of the (a) Les-6370, (b) Les-4403, (c) and Les-4272 interactions with tubulin in the colchicine binding site (PDB 4O2B); Figure S2 Root-mean-square fluctuation (RMSF) profiles of tubulin residues calculated from the 100 ns molecular dynamics trajectories of ligand–tubulin complexes at the colchicine-binding site (PDB ID: 4O2B): (a) Les-6370, (b) Les-4403, (c) Les-4309, (d) Les-4272, (e) Les-1419, and (f) combretastatin A4. Figure S3: 3D & 2D Schemes of the (a) Les-6370, (b) Les-4403, and (c) and Les-4272 interactions with tubulin in the taxane binding site (PDB 1JFF; Figure S4: Root-mean-square fluctuation (RMSF) profiles of tubulin residues calculated from the 100 ns molecular dynamics trajectories of ligand–tubulin complexes at the taxane-binding site (PDB ID: 1JFF): (a) Les-6370, (b) Les-4403, (c) Les-4309, (d) Les-4272, (e) Les-1419, and (f) paclitaxel.; Figure S5: Number of hydrogen bonds formed between tubulin and (a) Les-6370, (b) Les-4403, (c) Les-4309, (d) Les-4272, (e) Les-1419, and (f) combretastatin A-4 during the 100 ns molecular dynamics simulations at the colchicine-binding site (PDB ID: 4O2B). The plots show the time-dependent persistence and variation of ligand–protein hydrogen-bond interactions. Simulation time is expressed in ns.; Figure S6:-Radius of gyration (Rg) profiles of tubulin in complex with (a) Les-6370, (b) Les-4403, (c) Les-4309, (d) Les-4272, (e) Les-1419, and (f) combretastatin A-4 during the 100 ns molecular dynamics simulations at the colchicine-binding site (PDB ID: 4O2B).; Figure S7: Number of hydrogen bonds formed between tubulin and (a) Les-6370, (b) Les-4403, (c) Les-4309, (d) Les-4272, (e) Les-1419, and (f) paclitaxel during the 100 ns molecular dynamics simulations at the taxane-binding site (PDB ID: 1JFF).; Figures S8–S19: Copies of ^1^H, ^13^C NMR and LC-MS spectra of compounds Les 4272, Les 4309, Les 4403 and Les 6370; Figures S20–S23: Graphical representation of MTT assay results of Les-1419, Les-4272, Les-4309, Les-4403, Les-6370 and Dox after treatment; Figures S24–S29: -Anticancer activity screening results (NCI 60 lines screening) and influence of compounds Les-4272, Les-4309, and Les-4403 on the growth of tumor panels; Table S1: SwissTarget similarity prediction scores for the tubulin inhibitors; Table S2: SMILES of the virtual hit compounds; Table S3. Raw data of the obtained values from ELISA assessment of β-tubulin concentration in pg/mL.

## Abbreviations

The following abbreviations are used in this manuscript:

ADMET	Absorption, distribution, metabolism, excretion, and toxicity
ATCC	American Type Culture Collection
BBB	Blood–brain barrier
CHN	Carbon, hydrogen, and nitrogen
CYP	Cytochrome P450
DMEM	Dulbecco’s Modified Eagle Medium
DMF	N,N-Dimethylformamide
DMSO	Dimethyl sulfoxide
Dox	Doxorubicin
ELISA	Enzyme-linked immunosorbent assay
ESI	Electrospray ionization
GI	Gastrointestinal
GI_50_	Concentration causing 50% growth inhibition
GROMACS	GROningen MAchine for Chemical Simulations
IC_50_	Half-maximal inhibitory concentration
LC_50_	Concentration causing 50% cell death
LC–MS	Liquid chromatography–mass spectrometry
LD_50_	Median lethal dose
MD	Molecular dynamics
MMFF94	Merck Molecular Force Field 94
MTT	3-(4,5-Dimethylthiazol-2-yl)-2,5-diphenyltetrazolium bromide
MW	Molecular weight
NCI	National Cancer Institute
NMR	Nuclear magnetic resonance
NPT	Constant number of particles, pressure, and temperature ensemble
NVT	Constant number of particles, volume, and temperature ensemble
OPLS-AA	Optimized Potentials for Liquid Simulations–All Atom
PBS	Phosphate-buffered saline
PDB	Protein Data Bank
Rg	Radius of gyration
RMSD	Root mean square deviation
RMSF	Root mean square fluctuation
RPMI	Roswell Park Memorial Institute
RT	Room temperature
SAR	Structure–activity relationship
SI	Selectivity index
TGI	Total growth inhibition
TMB	3,3′,5,5′-Tetramethylbenzidine
TPSA	Topological polar surface area
UV–Vis	Ultraviolet–visible spectroscopy
ACPYPE	AnteChamber PYthon Parser interfacE
AM1-BCC	Austin Model 1 with Bond Charge Corrections
ANOVA	Analysis of Variance
COX-2	Cyclooxygenase-2
DTP	Developmental Therapeutics Program
EGFR	Epidermal Growth Factor Receptor
GAFF	General AMBER Force Field
GAFF2	General AMBER Force Field 2
HER2	Human Epidermal Growth Factor Receptor 2
NCI-60	National Cancer Institute 60 Human Tumor Cell Line Panel
PKB/Akt	Protein Kinase B/Akt
PTEN	Phosphatase and Tensin Homolog
SD	Standard Deviation
SEM	Standard Error of the Mean
SMILES	Simplified Molecular Input Line Entry System
VEGFR-2	Vascular Endothelial Growth Factor Receptor 2
LogS	Logarithm of aqueous solubility (mol/L)
MlogP	Moriguchi octanol–water partition coefficient (lipophilicity)
