# Supplementary material for: Identification of Novel Potential Tubulin-Targeting Heterocyclic Compounds via Integrated Virtual Screening and In Vitro Studies
Source: Pharmaceuticals (Basel). 2026 Oct 16;19(10):1637. doi: 10.3390/ph19101637 (PMC13708617; doi:10.3390/ph19101637)
Supplement: Supplementary file 1 [file pharmaceuticals-19-01637-s001.zip › pharmaceuticals-4595271-supplementary.pdf]

Supporting Information

Identification of Novel Potential Tubulin-Targeting Heterocyclic Compounds via Integrated Virtual Screening and In Vitro Studies

Yaroslava Deyak[1,2], Dmytro Khylyuk[3*], Rostyslav Dudchak[4], Nataliya Finiuk[5,6], Iryna Ivasechko[5,6], Yuliia Kozak[5,6], Nazar Solodiak[1,6], Halyna Derkach[7], Marta Pietruszyńska[8], Magdalena Podolak[9], Agnieszka Gornowicz[9], Krzysztof Bielawski[4], Anna Bielawska[9], Serhii Holota[1,6,*], Roman Lesyk[1,6,10.]

Table of Contents

- 3D & 2D Schemes of the a) Les-6370, b) Les-4403, c) and Les-4272 interactions with tubulin in the colchicine binding site (PDB 4O2B) ……………………….................Figure S1
- Root-mean-square fluctuation (RMSF) profiles of tubulin residues calculated from the 100 ns molecular dynamics trajectories of ligand–tubulin complexes at the colchicine-binding site (PDB ID: 4O2B): (a) Les-6370, (b) Les-4403, (c) Les-4309, (d) Les-4272, (e) Les-1419, and (f) combretastatin A4..…..Figure S2
- 3D & 2D Schemes of the a) Les-6370, b) Les-4403, and c) and Les-4272 interactions with tubulin in the taxane binding site (PDB 1JFF)…………………………………......Figure S3
- Root-mean-square fluctuation (RMSF) profiles of tubulin residues calculated from the 100 ns molecular dynamics trajectories of ligand–tubulin complexes at the taxane-binding site (PDB ID: 1JFF): (a) Les-6370, (b) Les-4403, (c) Les-4309, (d) Les-4272, (e) Les-1419, and (f) paclitaxel. ……….Figure S4
- The number of H-bonds formed between a) Les-6370, b) Les-4403, c) Les-4309, d) Les-4272, e) Les1419, and f) combretastatin A-4 (PDB 4O2B – colchicine binding site) during 100 ns simulation………………………………………………………………………Figure S5
- Radius of gyration (Rg) profiles of tubulin in complex with (a) Les-6370, (b) Les-4403, (c) Les-4309, (d) Les-4272, (e) Les-1419, and (f) combretastatin A4 during the 100 ns molecular dynamics simulations at the colchicine-binding site (PDB ID: 4O2B). ……………………………………………………………………………Figure S6
- Number of hydrogen bonds formed between tubulin and (a) Les-6370, (b) Les-4403, (c) Les-4309, (d) Les-4272, (e) Les-1419, and (f) paclitaxel during the 100 ns molecular dynamics simulations at the taxane-binding site (PDB ID: 1JFF). ……………………………………………………………………...Figure S7
- Copies of ^{1}H, ^{13}C NMR and LC-MS spectra of compounds Les 4272, Les 4309, Les 4403 and Les 6370…………..…………………………………………………....Figure S8–S19
- Graphical representation of MTT assay results of Les-1419, Les-4272, Les-4309, Les-4403, Les-6370 and Dox after treatment……………………………..…………Figure S20–S23
- Anticancer activity screening results (NCI 60 lines screening) and influence of compounds Les-4272, Les-4309, and Les-4403 on the growth of tumor panels..................Figure S24–S29
- SwissTarget similarity prediction scores for the tubulin inhibitors……………....…Table S1
- SMILES of the virtual hit compounds…………………………………………………Table S2
- Raw data of the obtained values from ELISA assessment of β-tubulin concentration in pg/mL……………………………………………………………………………...Table S3
-

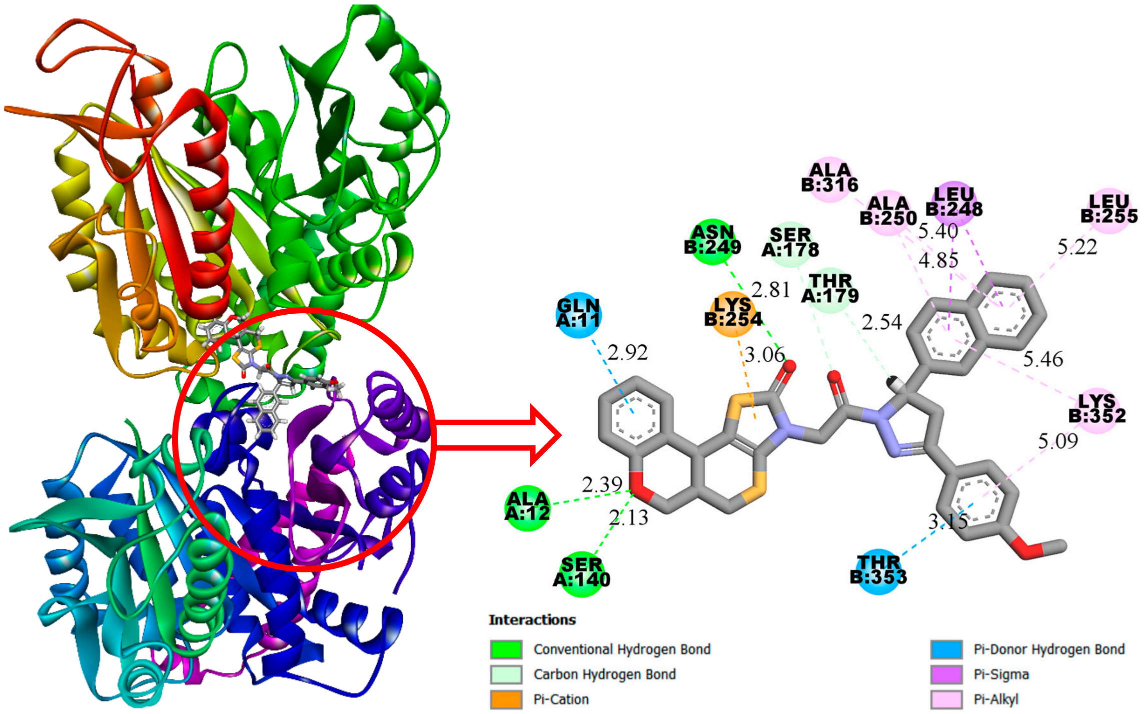
GLN A:11
ASN B:249
SER A:178
THR A:179
LYS B:254
ALA B:316
ALA B:250
LEU B:248
LEU B:255
LYS B:352
ALA A:12
SER A:140
THR B:353
2.92
2.81
3.06
2.54
5.40
4.85
5.22
5.46
5.09
2.39
2.13
3.15
Interactions
Conventional Hydrogen Bond
Carbon Hydrogen Bond
Pi-Cation
Pi-Donor Hydrogen Bond
Pi-Sigma
Pi-Alkyl

a)

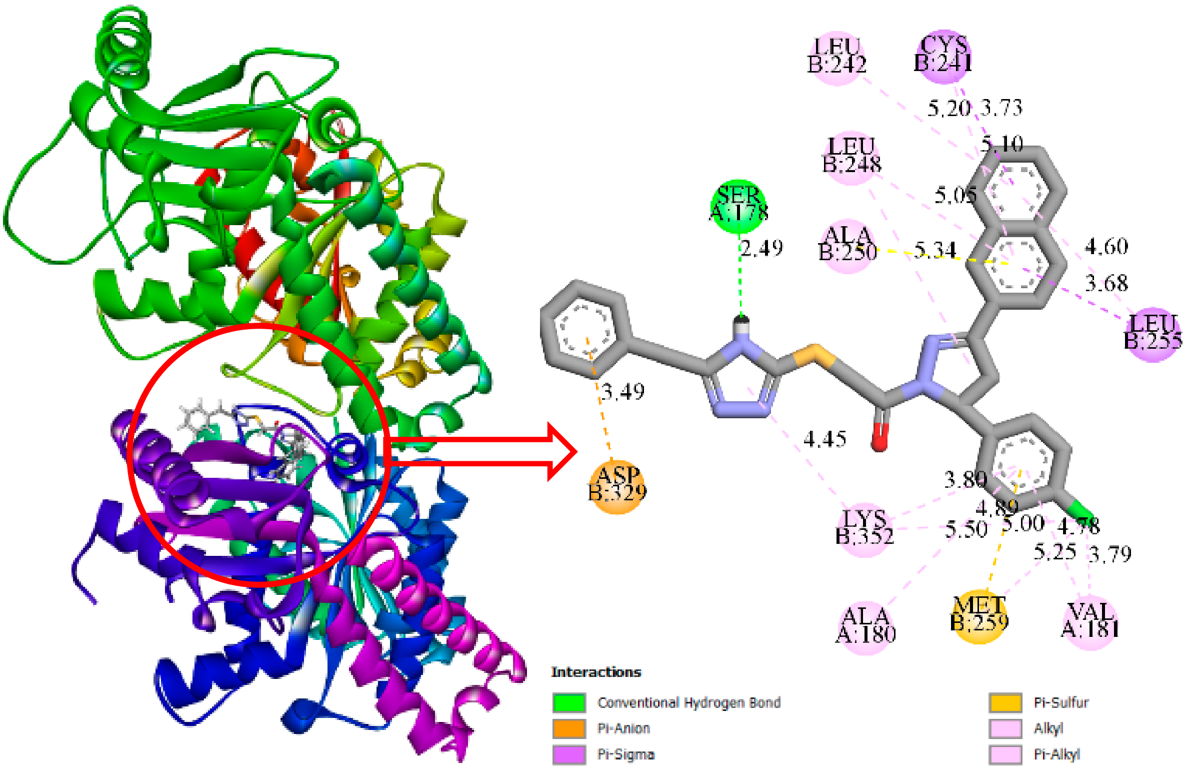
LEU B:242
CYS B:241
LEU B:248
SER A:178
ALA B:250
LEU B:255
ASP B:329
LYS B:352
ALA A:180
MET B:259
VAL A:181
5.20
3.73
5.10
5.05
2.49
5.34
4.60
3.68
3.49
4.45
3.80
4.89
5.50
5.00
4.78
5.25
3.79
Interactions
Conventional Hydrogen Bond
Pi-Anion
Pi-Sigma
Pi-Sulfur
Alkyl
Pi-Alkyl

b)

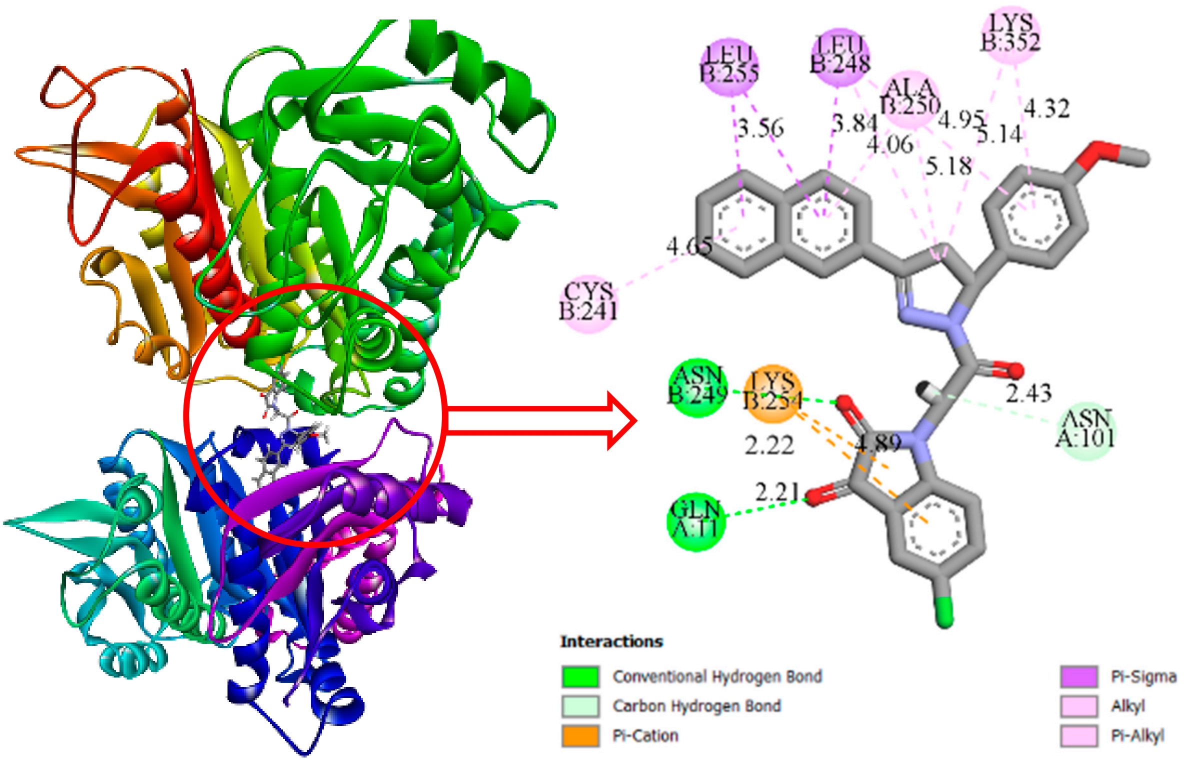

c)

Figure S1. 3D & 2D Schemes of the a) Les-6370, b) Les-4403, c) and Les-4272 interactions with tubulin in the colchicine binding site (PDB 4O2B).

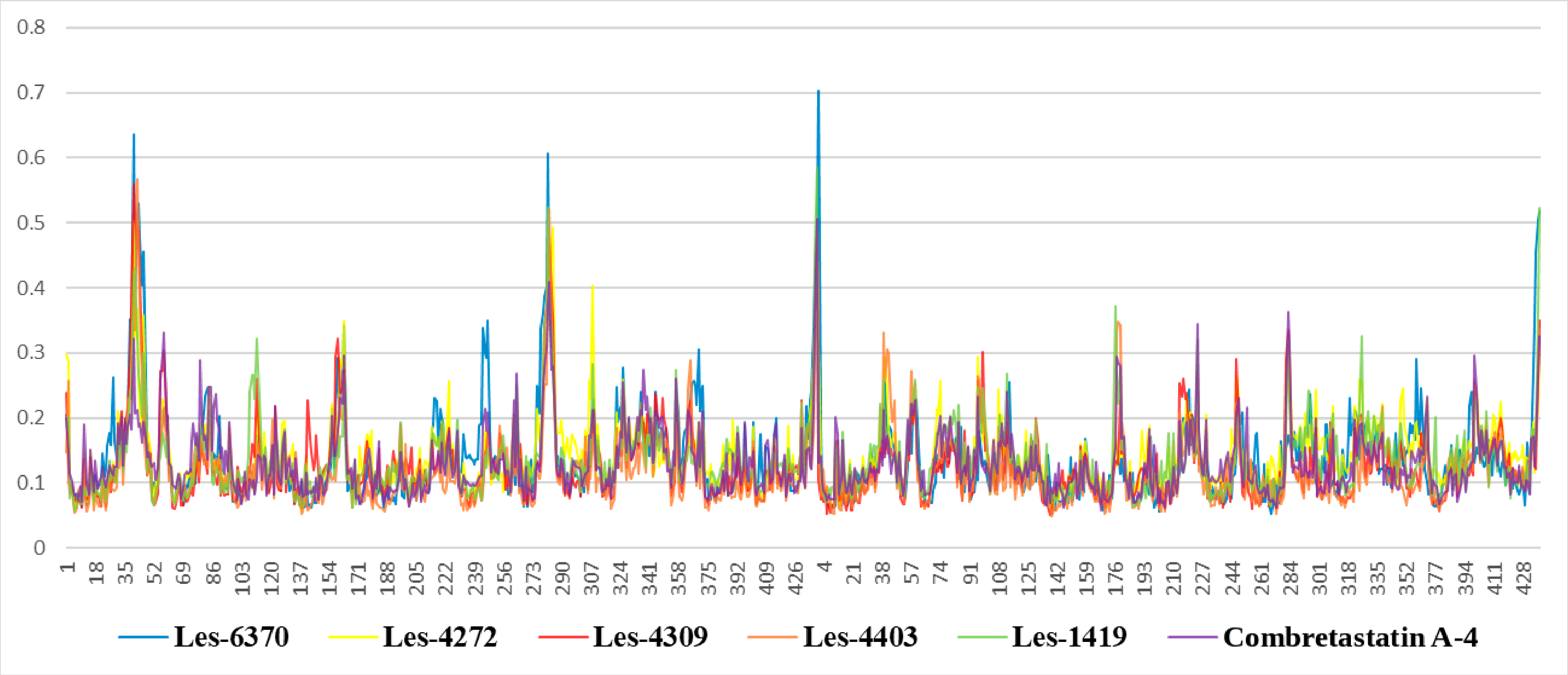

0.8
0.7
0.6
0.5
0.4
0.3
0.2
0.1
0
1 18 35 52 69 86 103 120 137 154 171 188 205 222 239 256 273 290 307 324 341 358 375 392 409 426 4 21 38 57 74 91 108 125 142 159 176 193 210 227 244 261 284 301 318 335 352 377 394 411 428
Les-6370
Les-4272
Les-4309
Les-4403
Les-1419
Combretastatin A-4

Figure S2. Root-mean-square fluctuation (RMSF) profiles of tubulin residues calculated from the 100 ns molecular dynamics trajectories of ligand–tubulin complexes at the colchicine-binding site (PDB ID: 4O2B): (a) Les-6370, (b) Les-4403, (c) Les-4309, (d) Les-4272, (e) Les-1419, and (f) combretastatin A-4. RMSF values, expressed in nm, describe the magnitude of positional fluctuations of individual protein residues during the simulation.

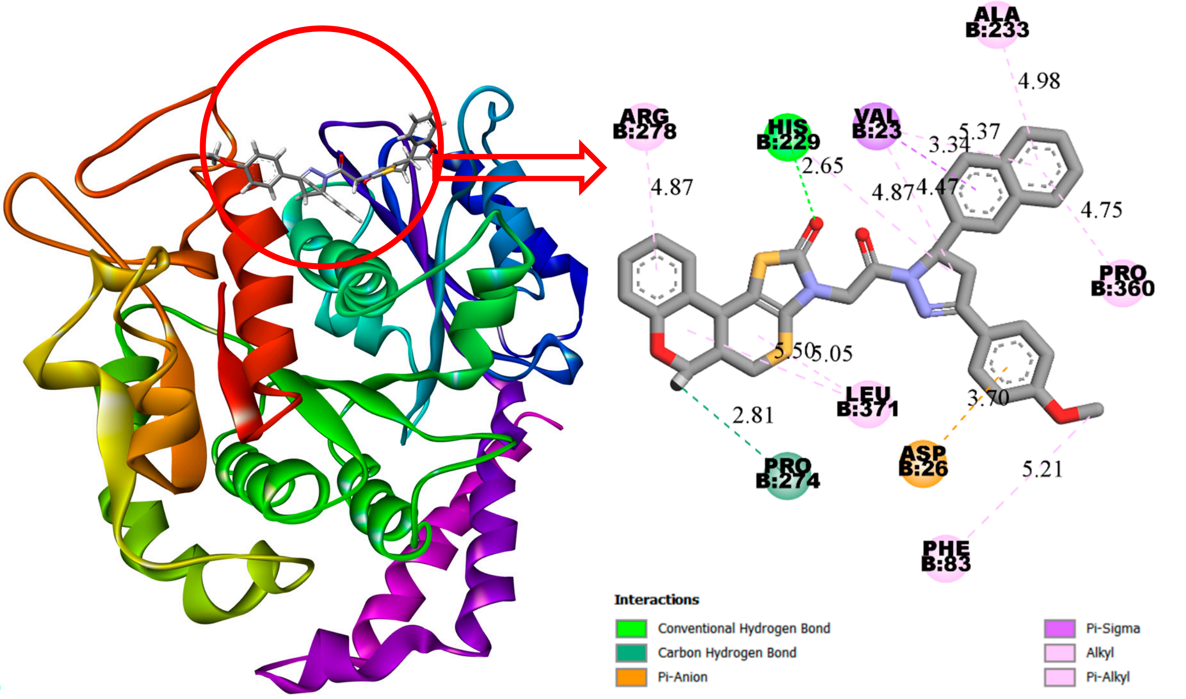
ALA B:233
4.98
ARG B:278
HIS B:229
VAL B:23
5.37
3.34
2.65
4.87
4.87
4.47
4.75
PRO B:360
5.50
5.05
LEU B:371
2.81
3.70
PRO B:274
ASP B:26
5.21
PHE B:83
Interactions
Conventional Hydrogen Bond
Carbon Hydrogen Bond
Pi-Anion
Pi-Sigma
Alkyl
Pi-Alkyl

a)

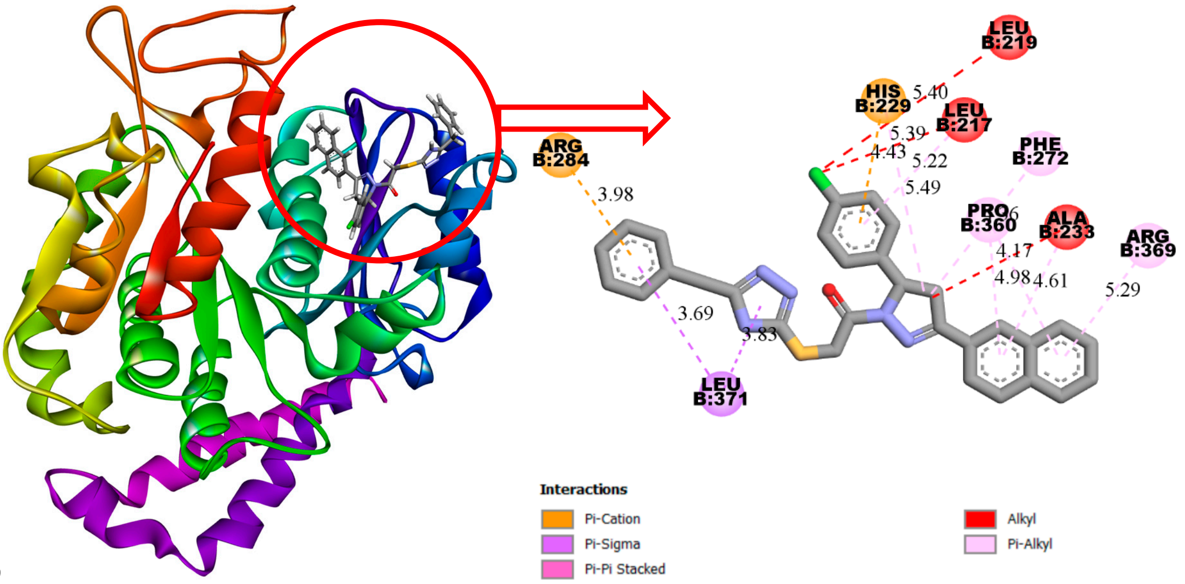
LEU B:219
HIS B:229
5.40
LEU B:217
5.39
4.43
5.22
PHE B:272
ARG B:284
5.49
3.98
PRO B:360
ALA B:233
ARG B:369
4.17
4.98
4.61
5.29
3.69
3.83
LEU B:371
Interactions
Pi-Cation
Pi-Sigma
Pi-Pi Stacked
Alkyl
Pi-Alkyl

b)

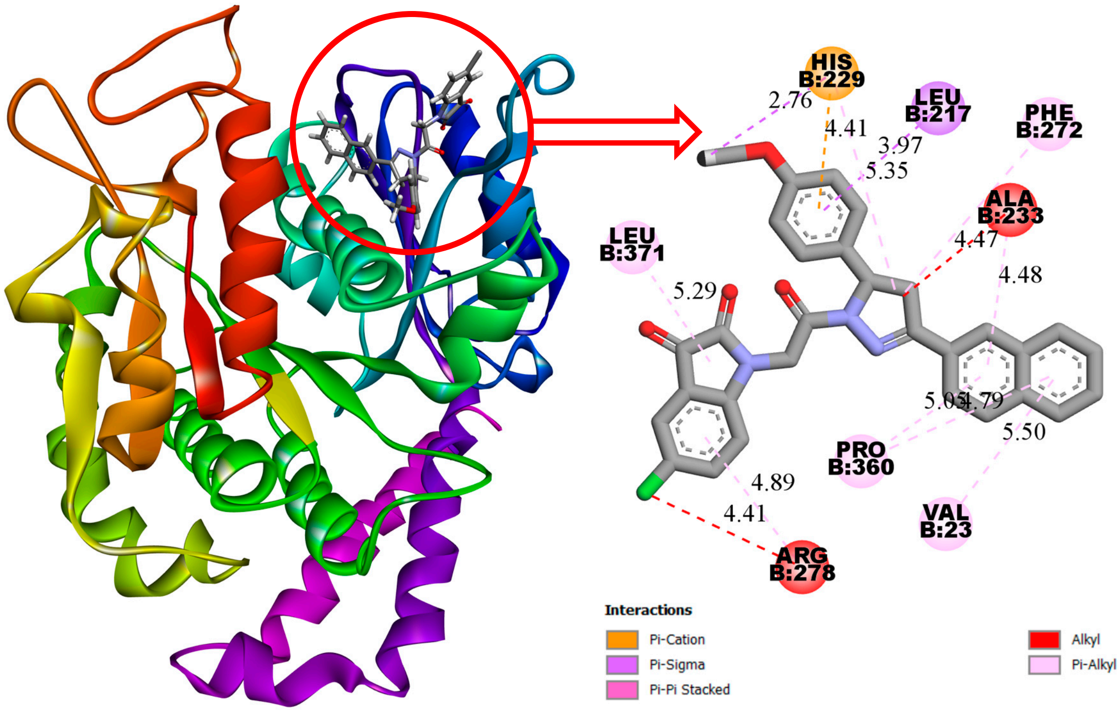

c)

Figure S3. 3D & 2D schemes of the a) Les-6370, b) Les-4403, and c) Les-4272 interaction with tubulin in the taxane binding site (PDB 1JFF)

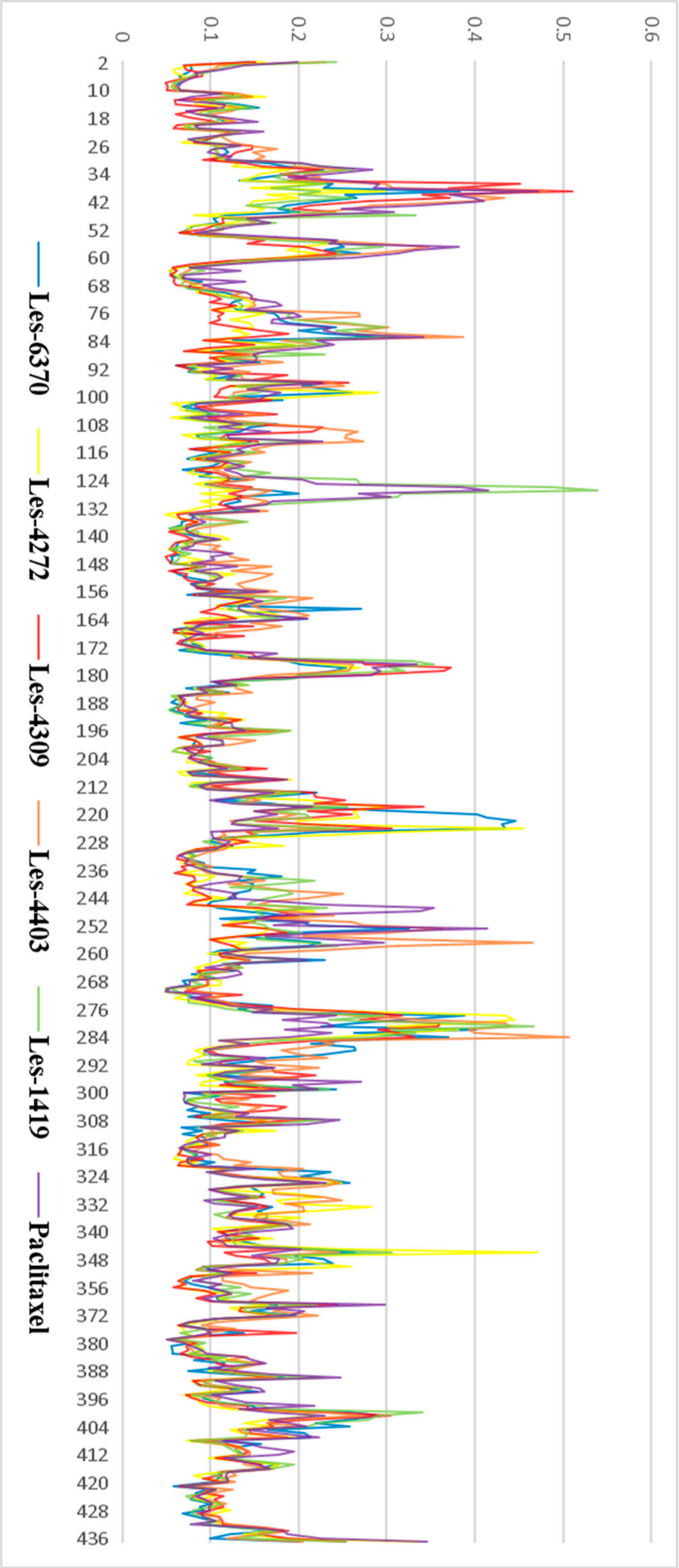

Figure S4. Root-mean-square fluctuation (RMSF) profiles of tubulin residues calculated from the 100 ns molecular dynamics trajectories of ligand–tubulin complexes at the taxane-binding site (PDB ID: 1JFF): (a) Les-6370, (b) Les-4403, (c) Les-4309, (d) Les-4272, (e) Les-1419, and (f) paclitaxel. RMSF values are expressed in nm and represent the positional fluctuations of individual protein residues during the simulation.

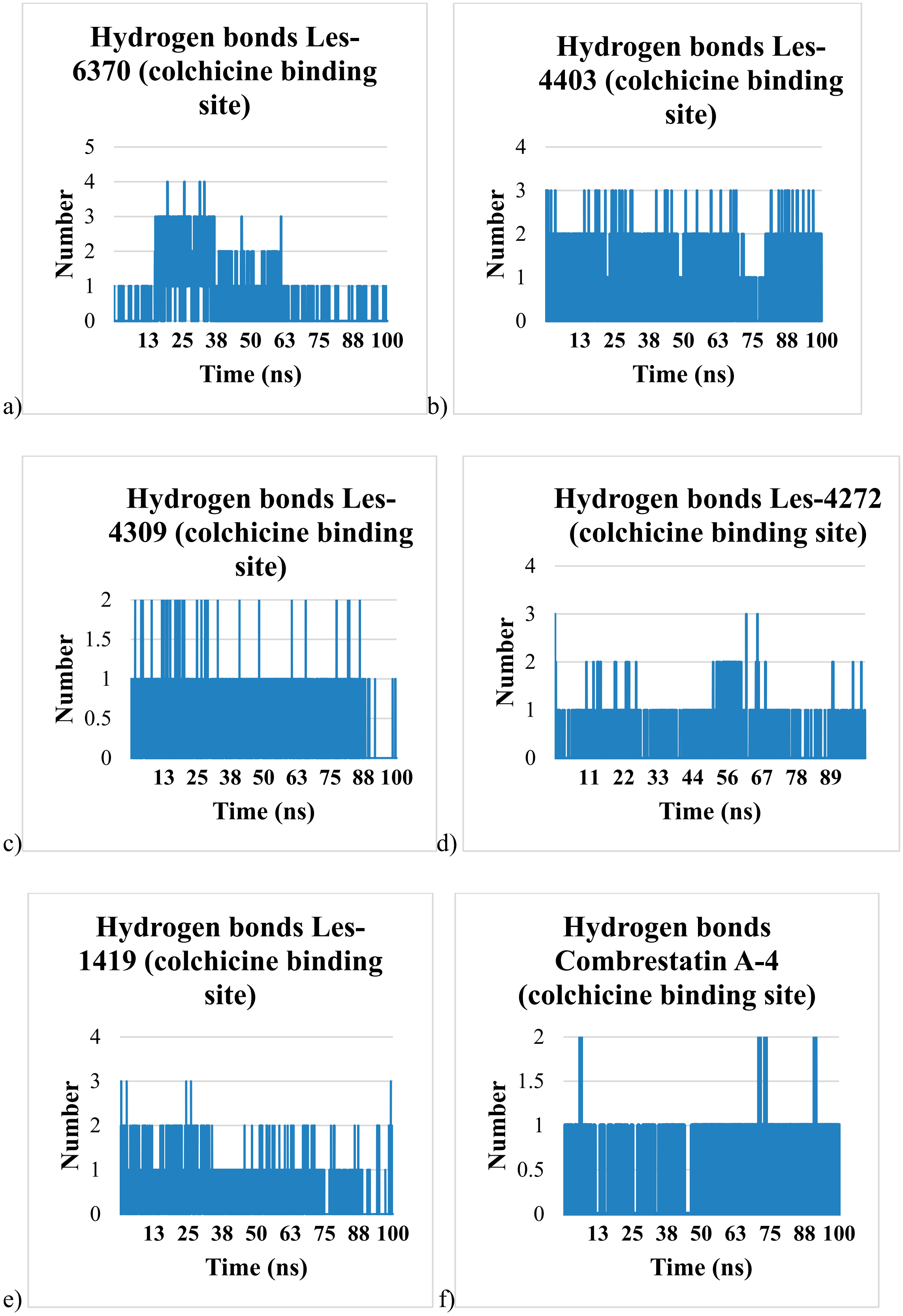

Figure S5. Number of hydrogen bonds formed between tubulin and (a) Les-6370, (b) Les-4403, (c) Les-4309, (d) Les-4272, (e) Les-1419, and (f) combretastatin A-4 during the 100 ns molecular dynamics simulations at the colchicine-binding site (PDB ID: 4O2B). The plots show the time-dependent persistence and variation of ligand–protein hydrogen-bond interactions. Simulation time is expressed in ns.

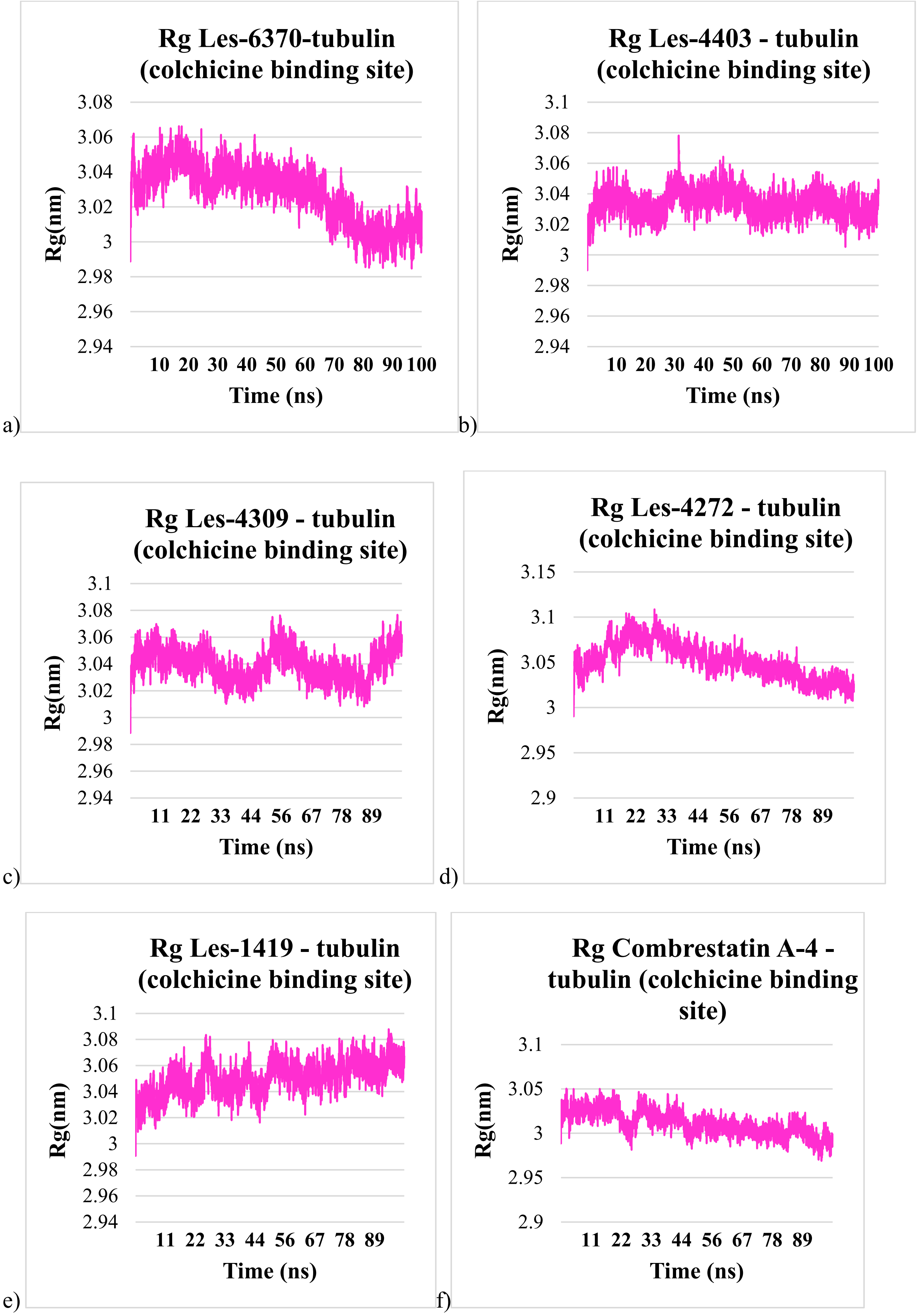

Figure S6. Radius of gyration (Rg) profiles of tubulin in complex with (a) Les-6370, (b) Les-4403, (c) Les-4309, (d) Les-4272, (e) Les-1419, and (f) combretastatin A-4 during the 100 ns molecular dynamics simulations at the colchicine-binding site (PDB ID: 4O2B). Rg values are expressed in nm and describe changes in the overall compactness of the protein structure during the simulation.

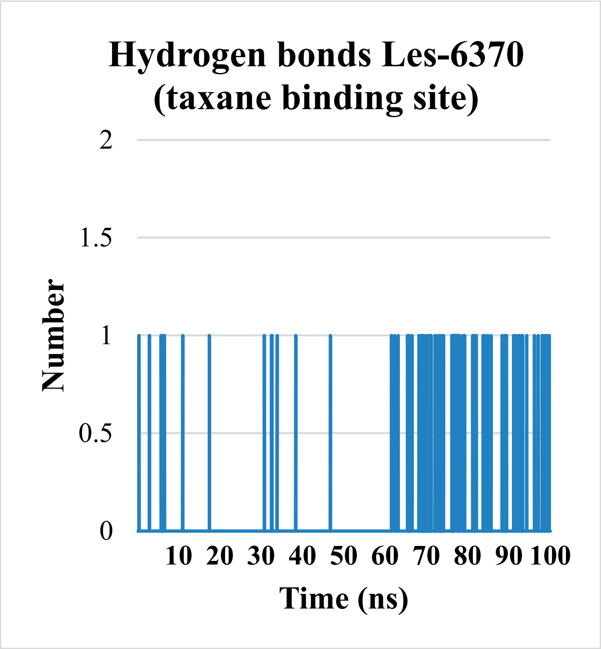
Hydrogen bonds Les-6370
(taxane binding site)
Number
Time (ns)
0
0.5
1
1.5
2
10 20 30 40 50 60 70 80 90 100

a)

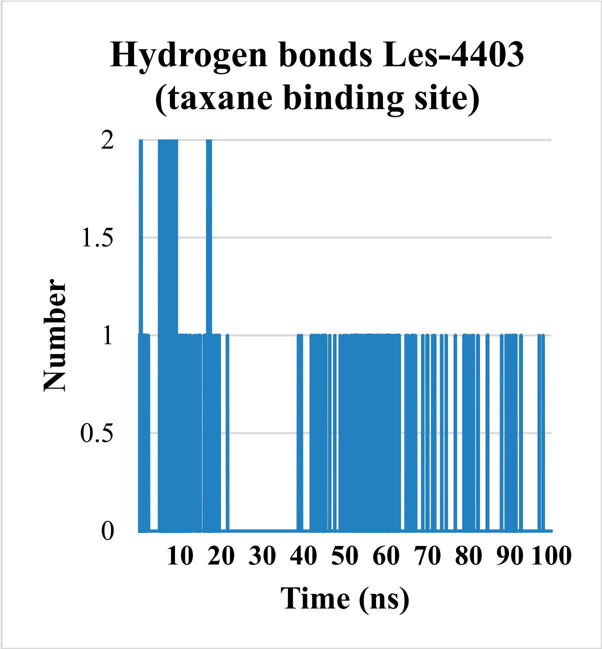
Hydrogen bonds Les-4403
(taxane binding site)
Number
Time (ns)
0
0.5
1
1.5
2
10 20 30 40 50 60 70 80 90 100

b)

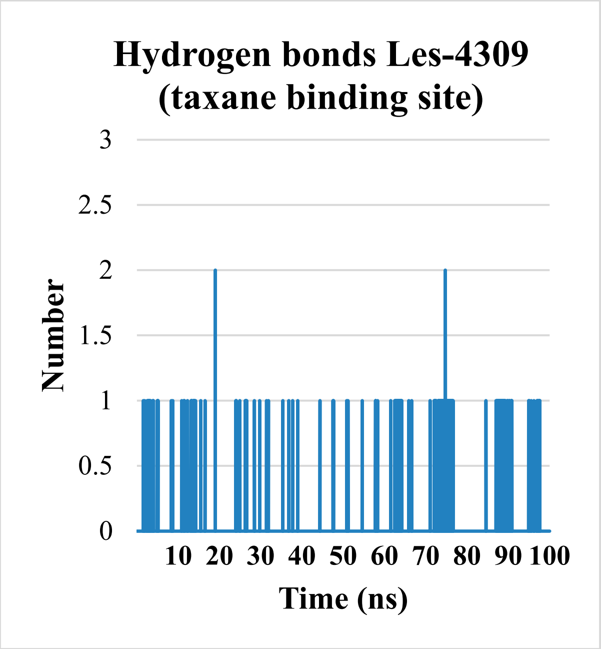
Hydrogen bonds Les-4309
(taxane binding site)
Number
Time (ns)
0
0.5
1
1.5
2
2.5
3
10 20 30 40 50 60 70 80 90 100

c)

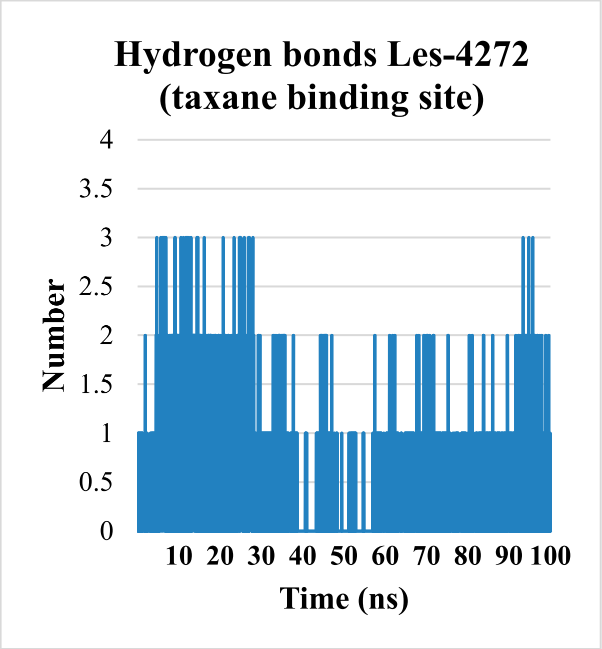
Hydrogen bonds Les-4272
(taxane binding site)
Number
Time (ns)
0
0.5
1
1.5
2
2.5
3
3.5
4
10 20 30 40 50 60 70 80 90 100

d)

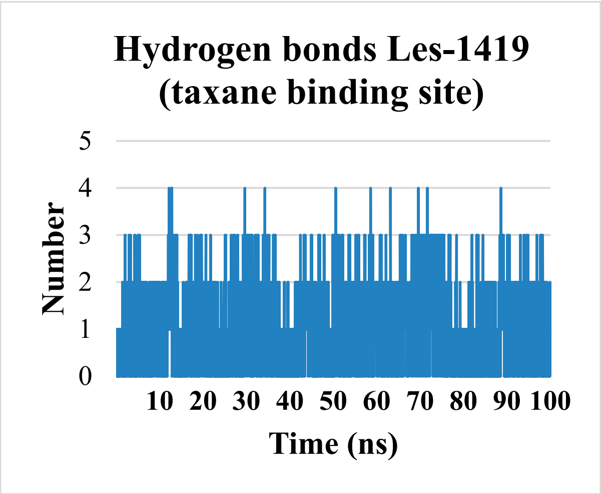
Hydrogen bonds Les-1419
(taxane binding site)
Number
Time (ns)
0
1
2
3
4
5
10 20 30 40 50 60 70 80 90 100

e)

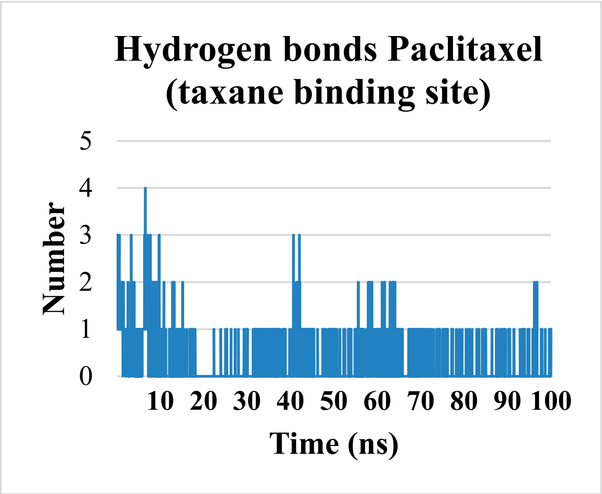
Hydrogen bonds Paclitaxel
(taxane binding site)
Number
Time (ns)
0
1
2
3
4
5
10 20 30 40 50 60 70 80 90 100

f)

Figure S7. Number of hydrogen bonds formed between tubulin and (a) Les-6370, (b) Les-4403, (c) Les-4309, (d) Les-4272, (e) Les-1419, and (f) paclitaxel during the 100 ns molecular dynamics simulations at the taxane-binding site (PDB ID: 1JFF). The plots show the time-dependent persistence and variation of ligand–protein hydrogen-bond interactions. Simulation time is expressed in ns.

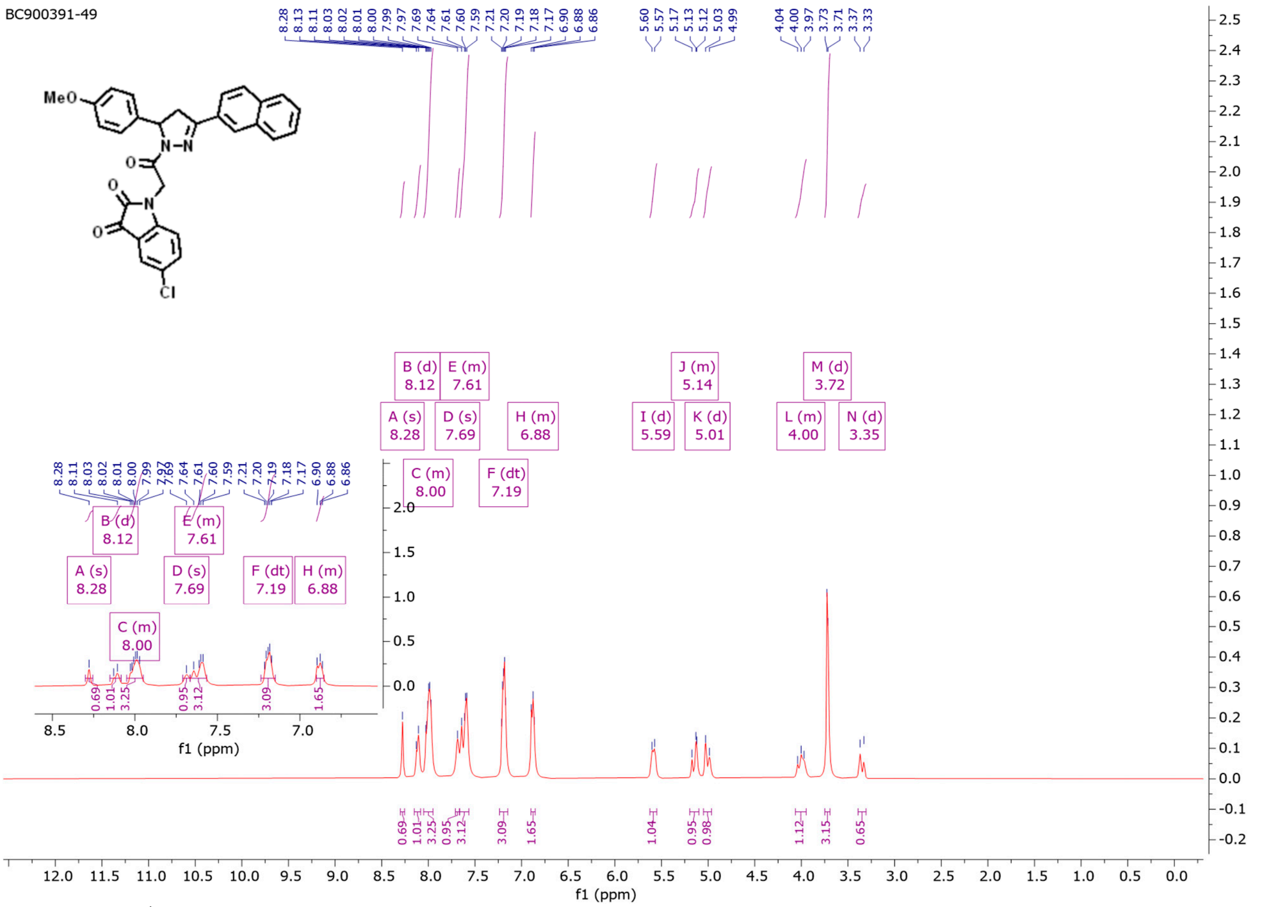

Figure S8. ^{1}H NMR spectrum of Les 4272

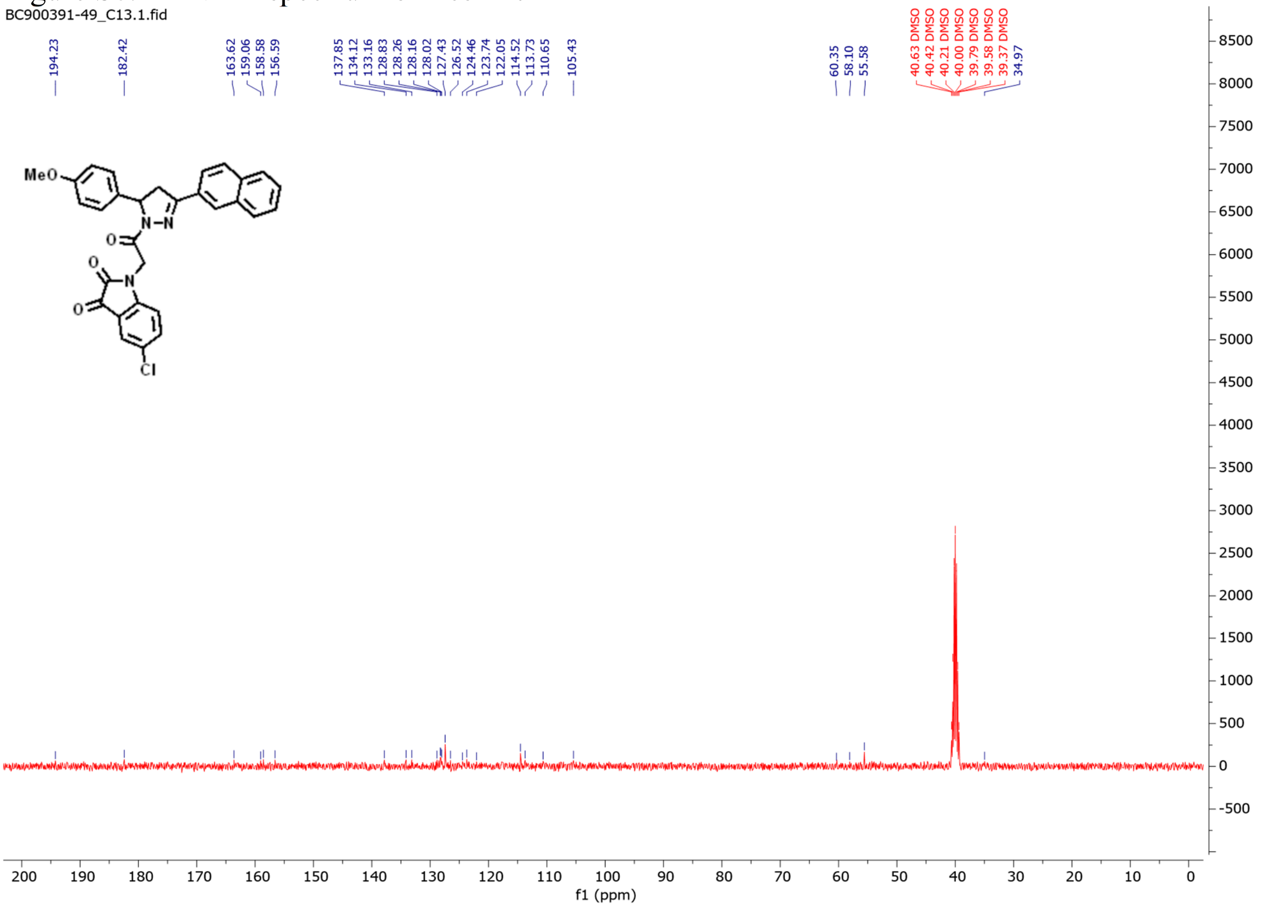

Figure S9. ^{13}C NMR spectrum of Les 4272

#	RT	DAD1A	DAD1B	MSD1	MSD2	ELSD	MSD1 ions	MSD1 rt	MSD2 ions	MSD2 rt	Inf
1	1.306	99.0%	99.2%	99.0%	99.4%	100.0%	524.0(99),546.0(1)	1.311	---	---	

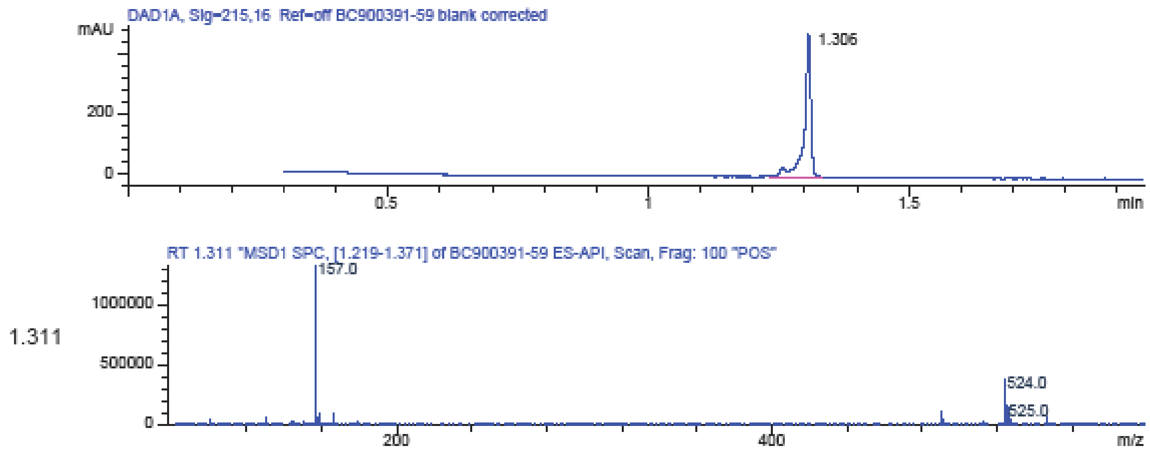

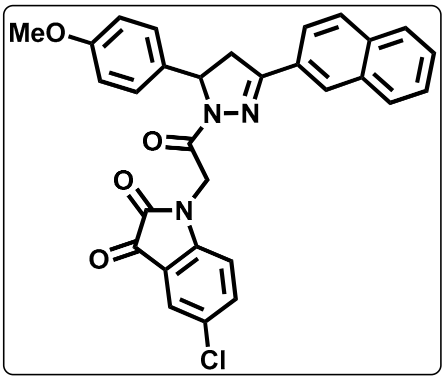

Molecular Weight: 523,97

Figure S10. LC-MS spectrum of Les 4272

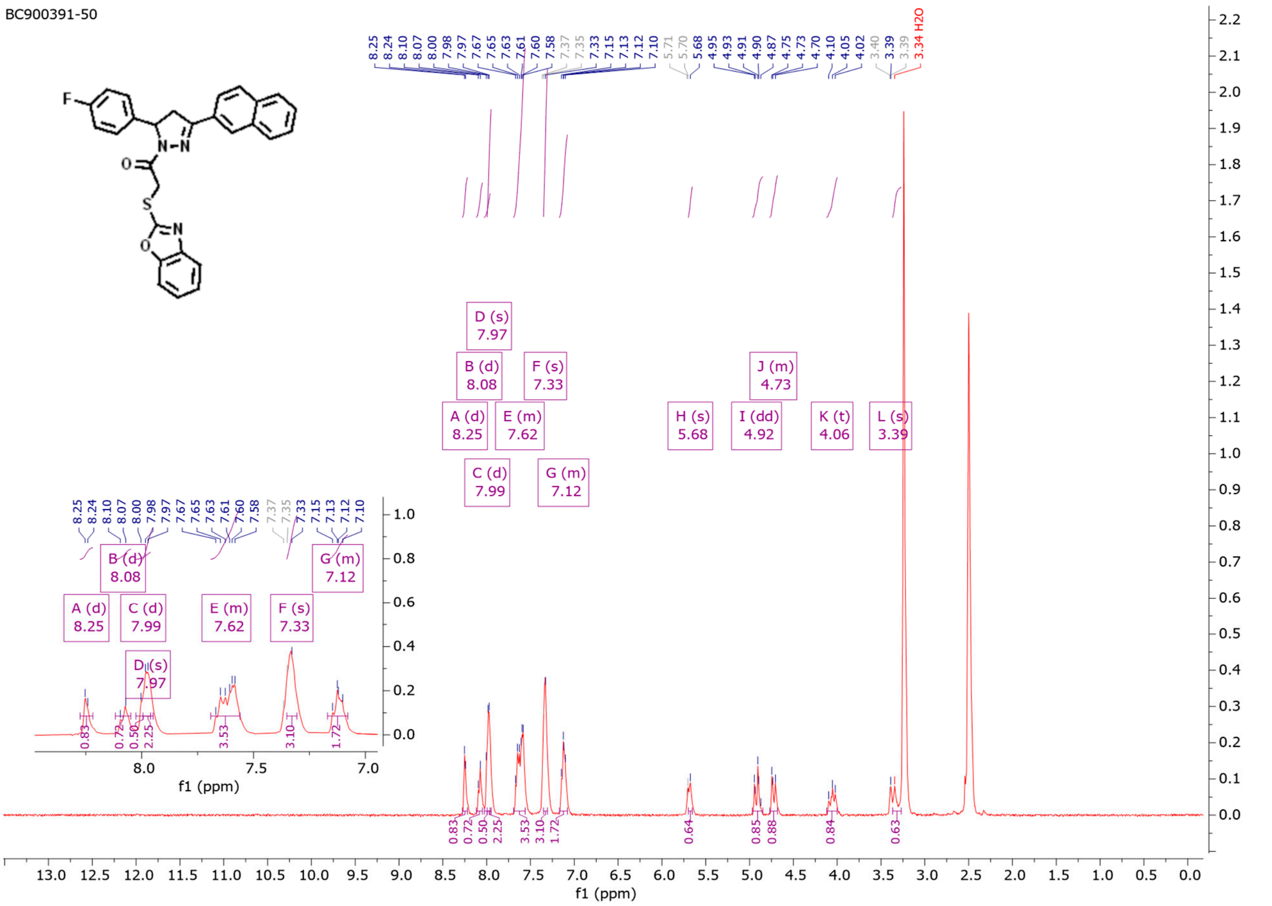

Figure S11. ^{1}H NMR spectrum of Les 4309

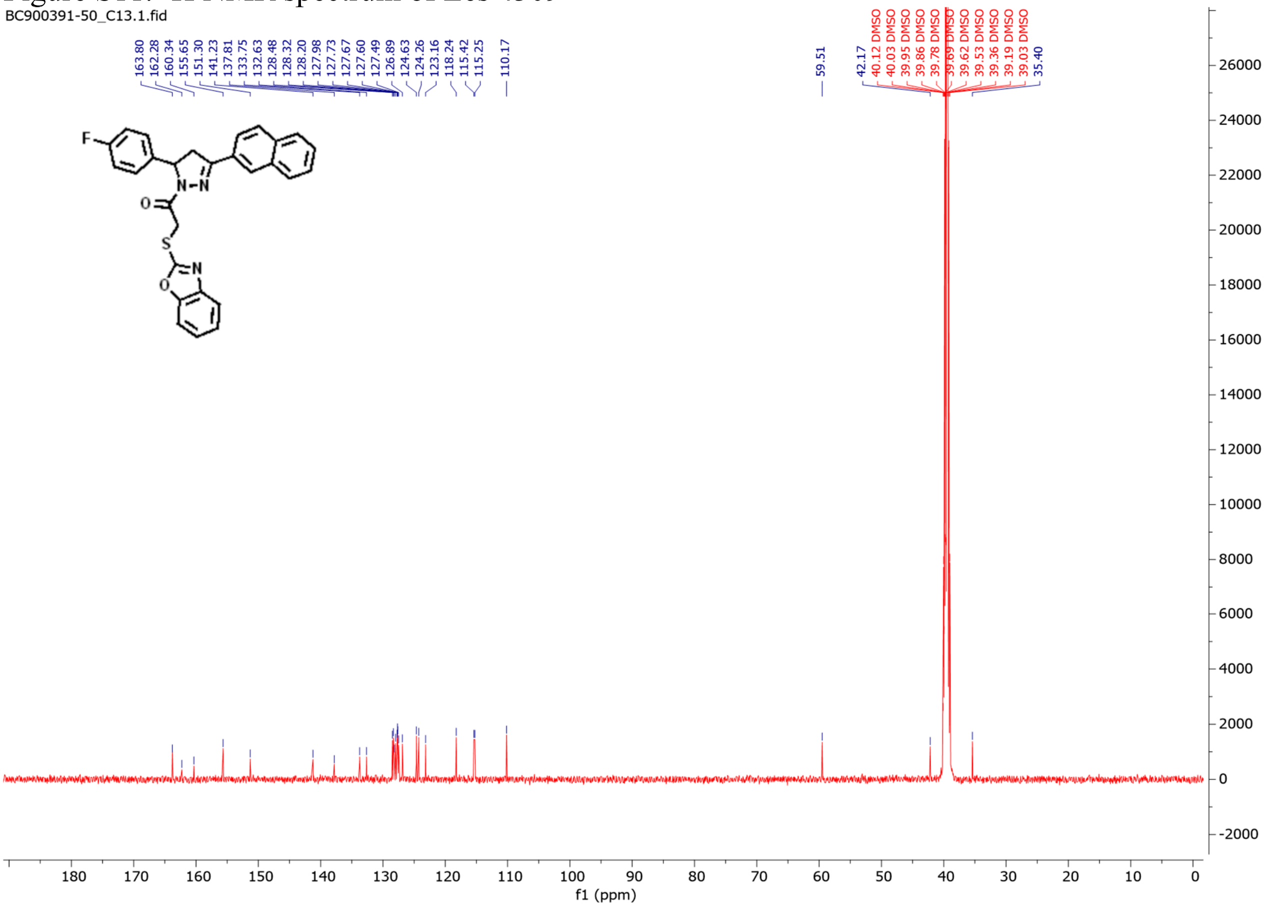

Figure S12. ^{13}C NMR spectrum of Les 4309

#	RT	DAD1A	DAD1B	MSD1	MSD2	ELSD	MSD1 ions	MSD1 rt	MSD2 ions	MSD2 rt	Info
1	1.507	100.0%	100.0%	100.0%	---	100.0%	482.0(98),504.0(2)	1.520	---	---	

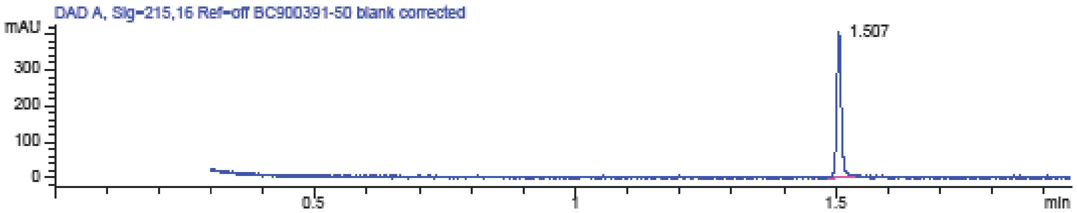

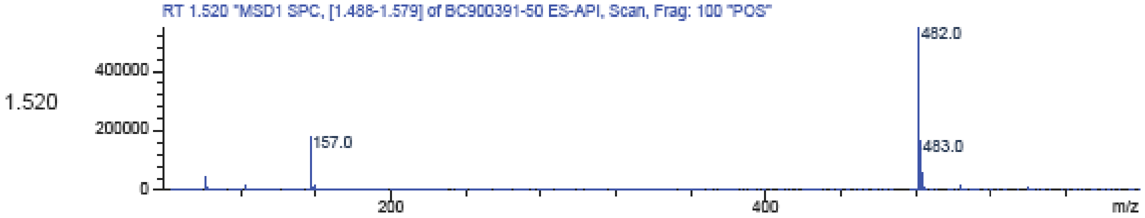

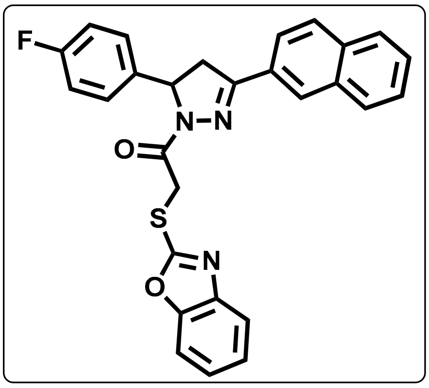

Molecular Weight: 481,55

Figure S13. LC-MS spectrum of Les 4309

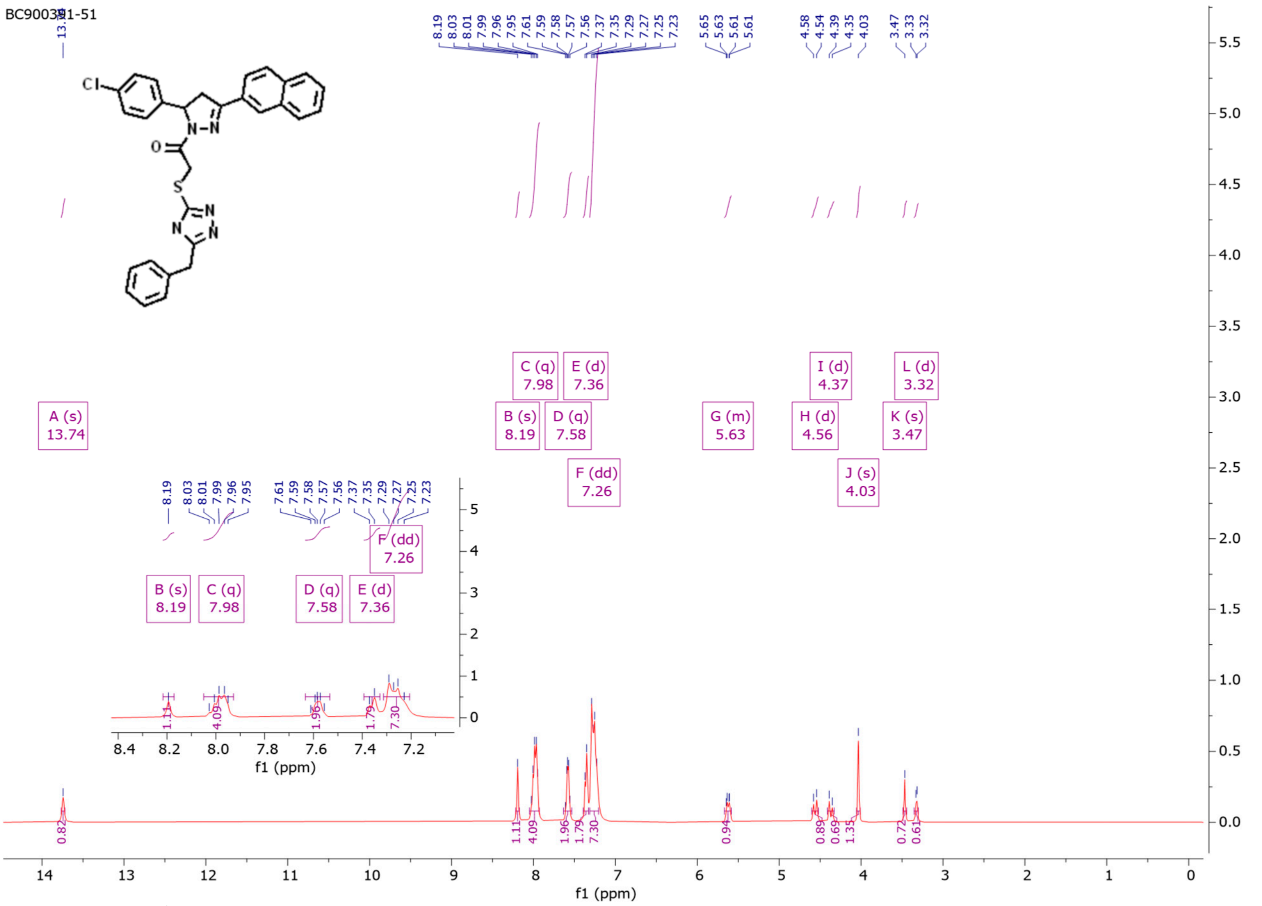

Figure S14. ^{1}H NMR spectrum of Les 4403

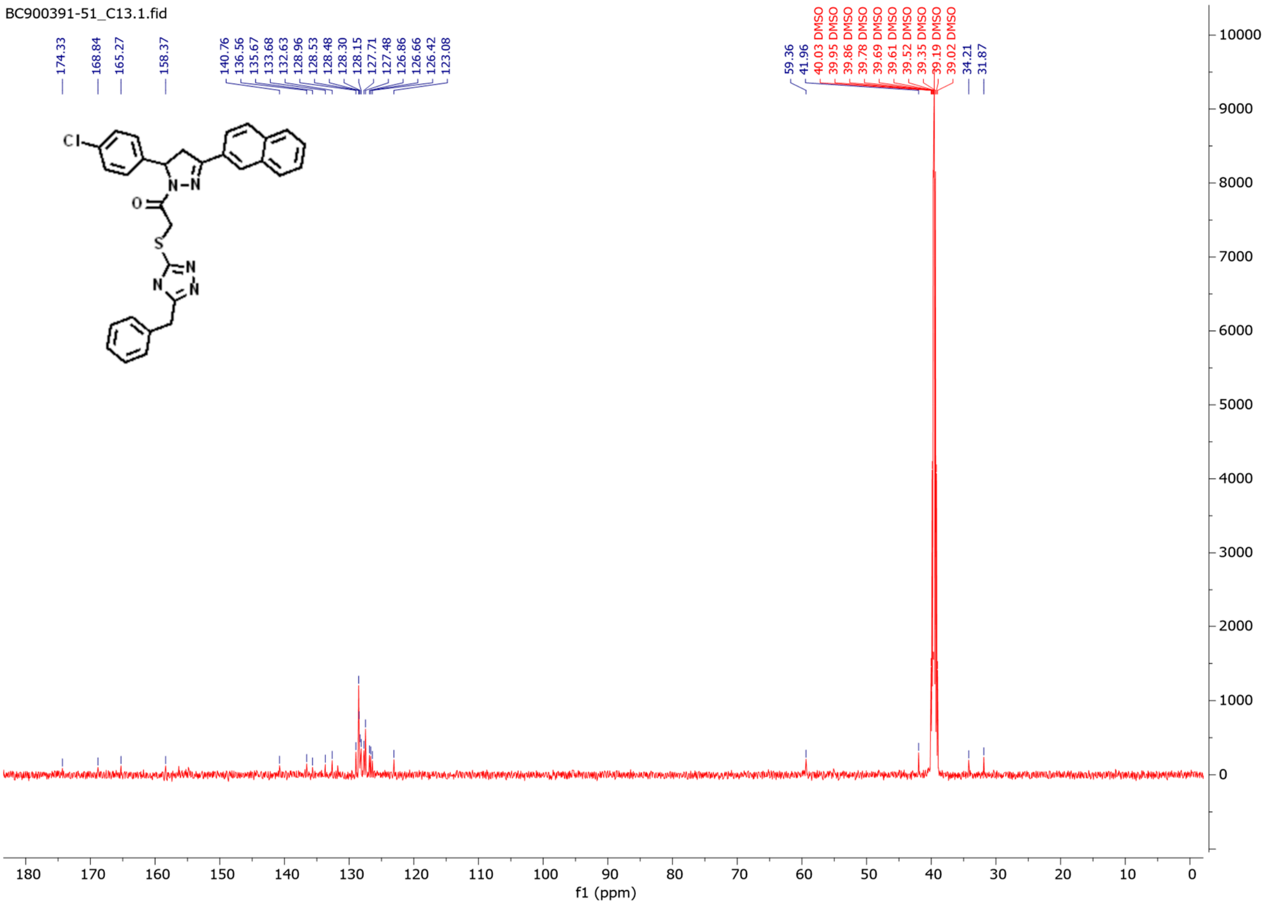

Figure S15. ^{13}C NMR spectrum of Les 4403

#	RT	DAD1A	DAD1B	MSD1	MSD2	ELSD	MSD1 ions	MSD1 rt	MSD2 ions	MSD2 rt	Info
1	1.628	99.1%	97.7%	99.1%	99.1%	99.1%	538.0(100)	1.650	536.0(83),571.8(7),598.8(6)	1.649	

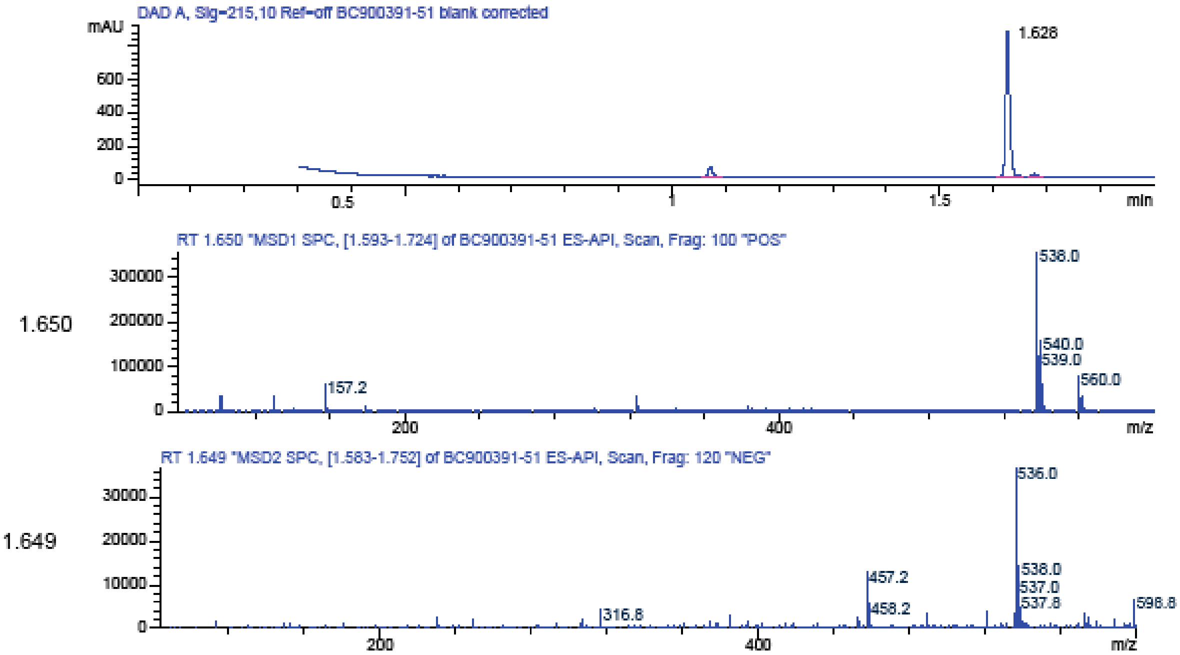

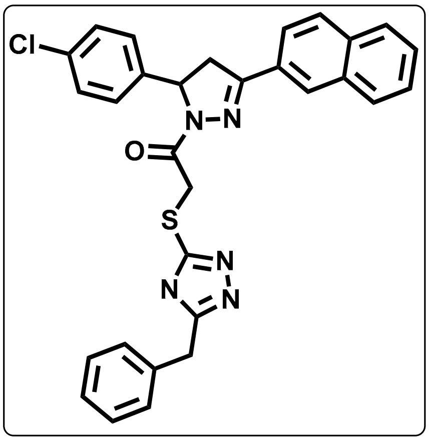

Molecular Weight: 538,07

Figure S16. LC-MS spectrum of Les 4403

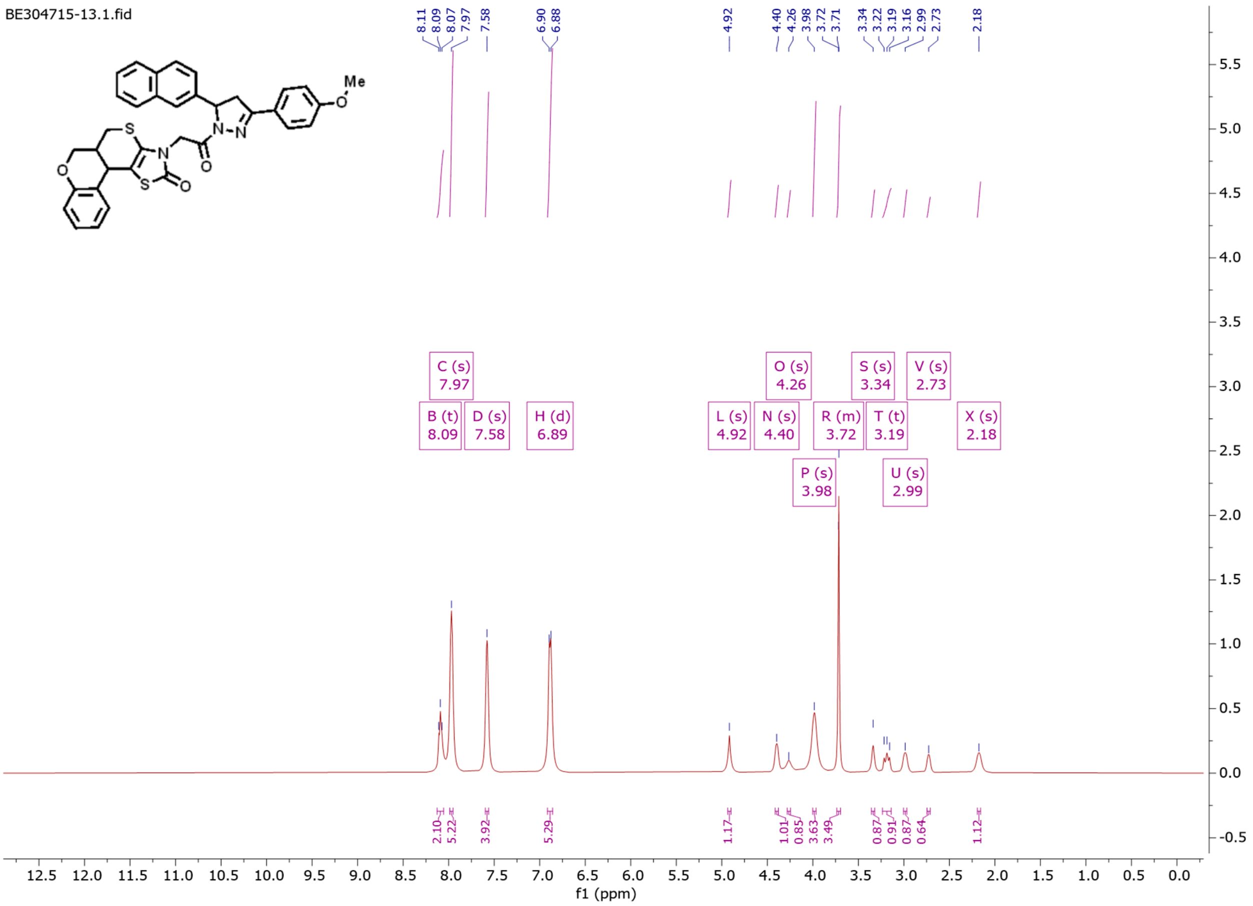

Figure S17. ^{1}H NMR spectrum of Les 6370

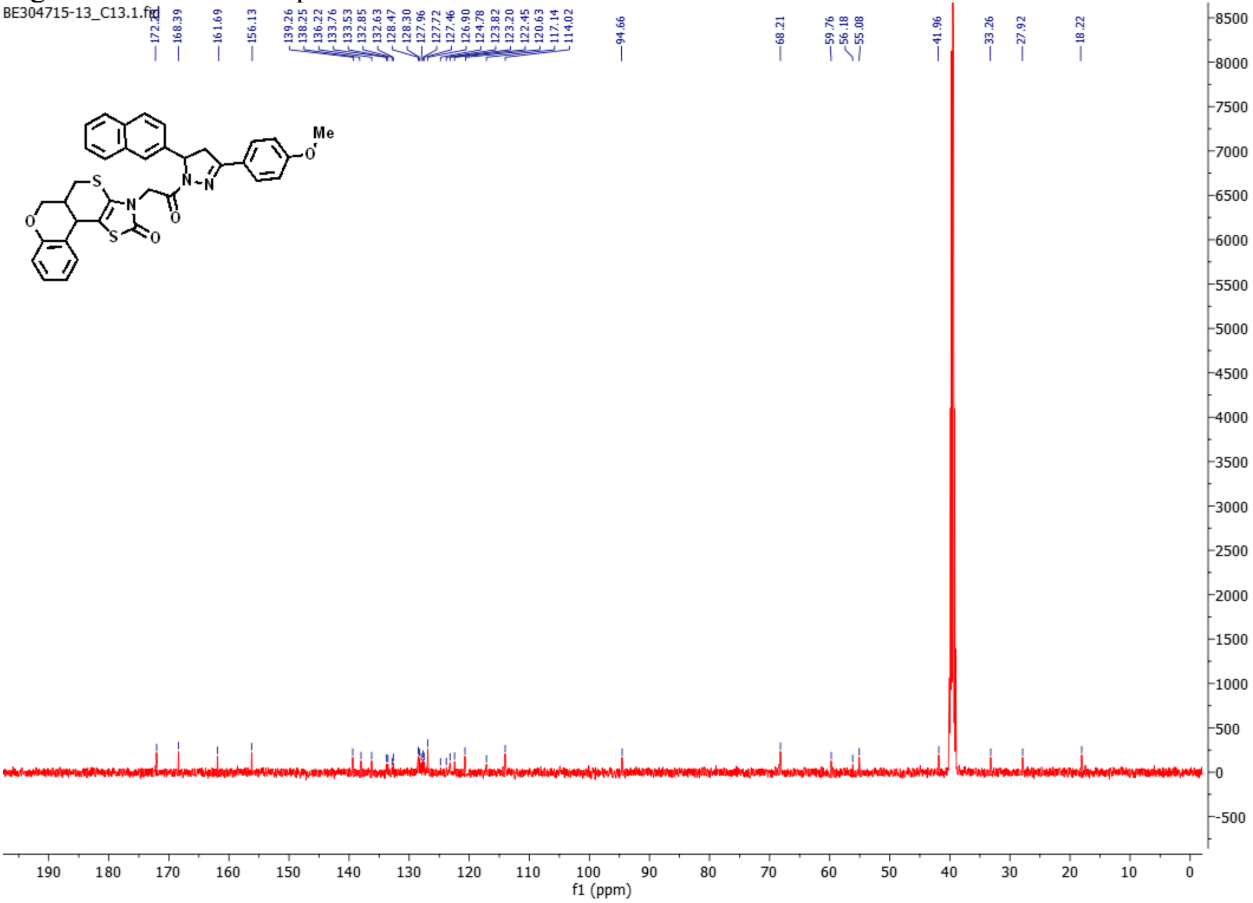

Figure S18. ^{13}C NMR spectrum of Les 6370

BE304715-13

L883224R
LCMS-26
SUPOR_30.M
12:12 20.02.2025
Product not found
No product

#	RT	DAD1A	DAD1B	MSD1	MSD2	ELSD	MSD1 ions	MSD1 rt	MSD2 ions	MSD2 rt	Info
1	1.683	100.0%	100.0%	100.0%	100.0%	---	620.2(96),613.0(4)	1.650	---	---	

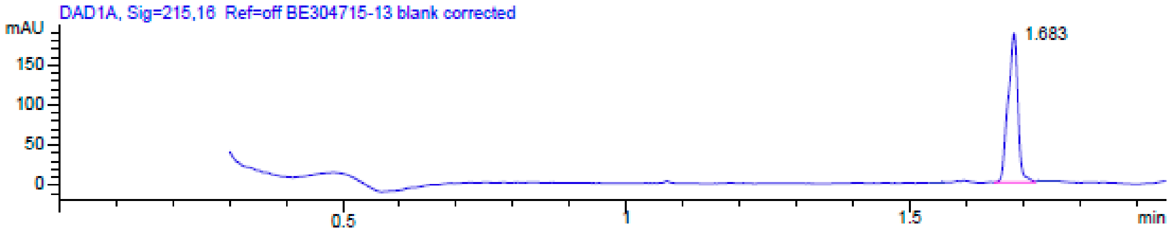

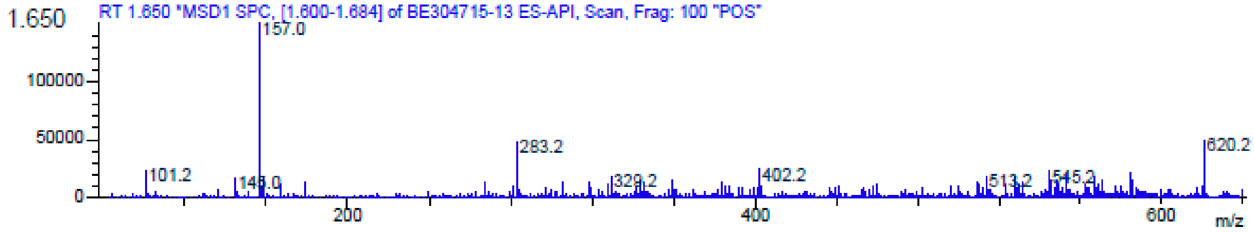

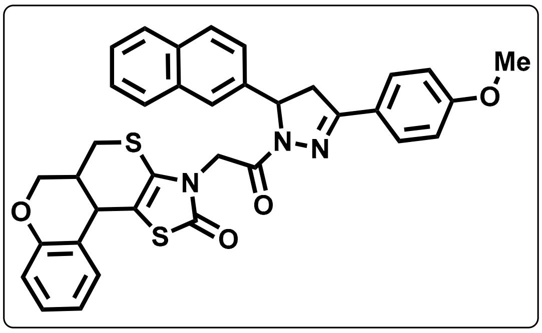

Molecular Weight: 619,75

Figure S19. LC-MS spectrum of Les 6370

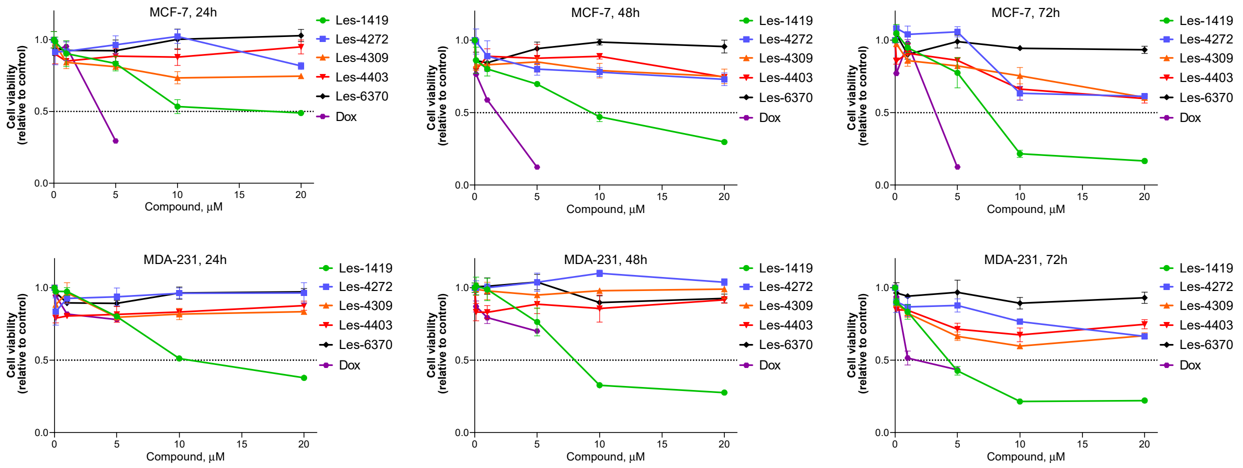

Figure S20. Graphical representation of MTT assay results of Les-1419, Les-4272, Les-4309, Les-4403, Les-6370 and Dox against MCF-7 and MDA-MB-231 after 24, 48 and 72 hours of treatment.

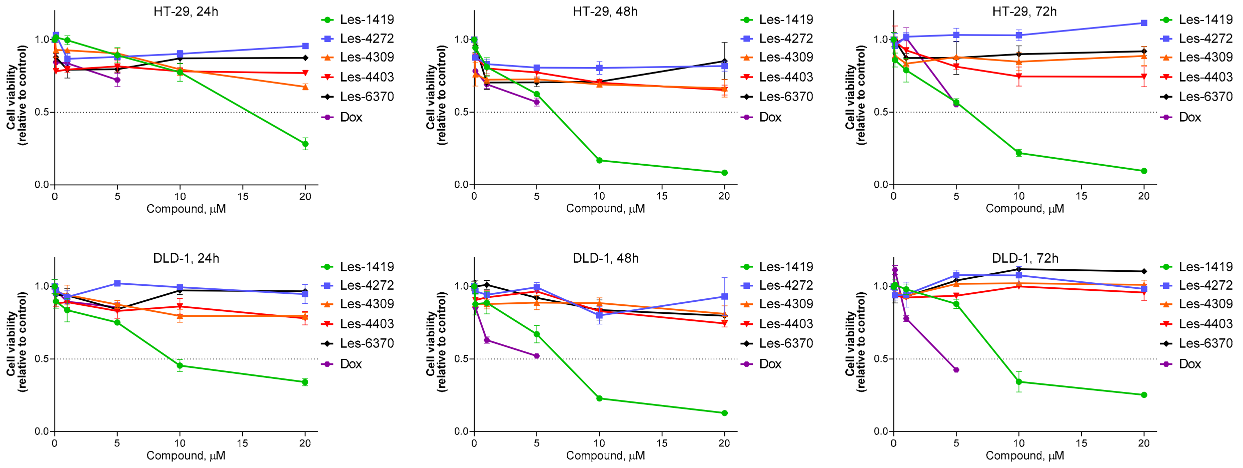

Figure S21. Graphical representation of MTT assay results of Les-1419, Les-4272, Les-4309, Les-4403, Les-6370 and Dox against HT-29 and DLD-1 after 24, 48 and 72 hours of treatment.

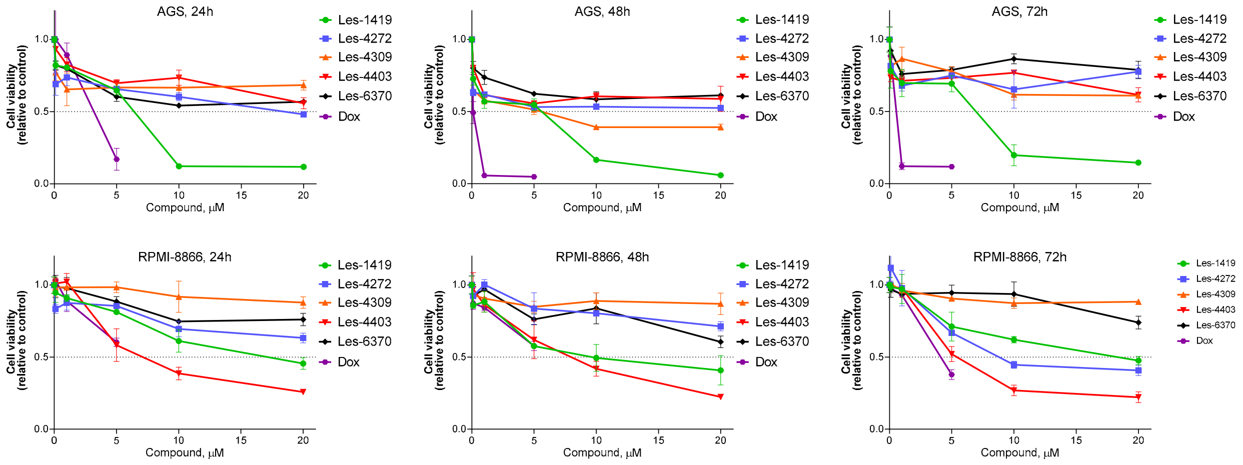

Figure S22. Graphical representation of MTT assay results of Les-1419, Les-4272, Les-4309, Les-4403, Les-6370 and Dox against AGS and RPMI-8866 after 24, 48 and 72 hours of treatment.

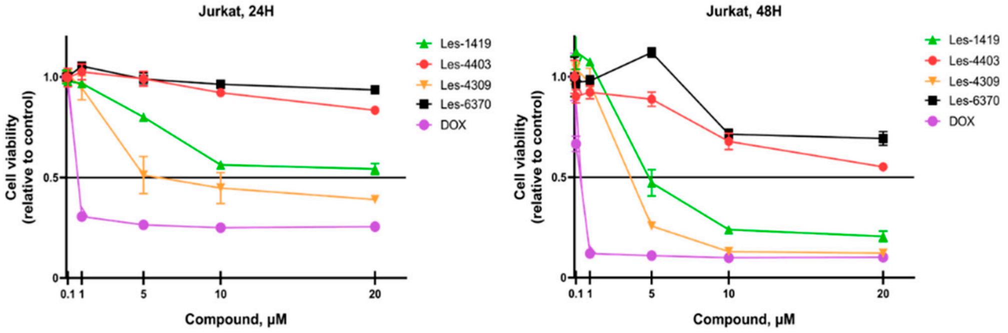

Figure S23. Graphical representation of MTT assay results of Les-1419, Les-4272, Les-4309, Les-4403, Les-6370 and Dox against Jurkat after 24, and 48 hours of treatment.

Developmental Therapeutics Program One Dose Mean Graph	NSC: D-760917 / 1	Conc: 1.00E-5 Molar	Test Date: Aug 08, 2011
	Experiment ID: 1108OS00		Report Date: Feb 05, 2024

Panel/Cell Line	Growth Percent
**Leukemia**	
CCRF-CEM	17.14
HL-60(TB)	2.06
MOLT-4	4.80
RPMI-8226	21.46
**Non-Small Cell Lung Cancer**	
A549/ATCC	109.14
EKVX	89.68
HOP-62	81.13
NCI-H226	84.55
NCI-H23	68.89
NCI-H322M	87.93
NCI-H460	110.16
NCI-H522	50.93
**Colon Cancer**	
COLO 205	111.85
HCC-2998	103.73
HCT-116	8.29
HCT-15	57.56
HT29	53.55
KM12	20.48
SW-620	16.62
**CNS Cancer**	
SF-268	75.19
SF-295	88.46
SF-539	32.56
SNB-19	81.09
SNB-75	86.98
U251	41.32
**Melanoma**	
LOX IMVI	86.68
MALME-3M	92.35
M14	91.01
MDA-MB-435	98.90
SK-MEL-2	124.37
SK-MEL-28	98.60
SK-MEL-5	103.26
UACC-257	100.07
UACC-62	80.28
**Ovarian Cancer**	
IGROV1	63.64
OVCAR-3	28.66
OVCAR-4	29.74
OVCAR-5	96.46
OVCAR-8	49.04
NCI/ADR-RES	81.35
SK-OV-3	98.43
**Renal Cancer**	
786-0	56.47
A498	98.95
ACHN	69.31
CAKI-1	74.52
RXF 393	85.14
SN12C	77.20
TK-10	87.17
UO-31	64.19
**Prostate Cancer**	
PC-3	31.00
DU-145	101.74
**Breast Cancer**	
MCF7	19.94
MDA-MB-231/ATCC	88.99
HS 578T	83.97
BT-549	86.49
T-47D	42.27
MDA-MB-468	99.60
Mean	70.09
Delta	68.03
Range	122.31

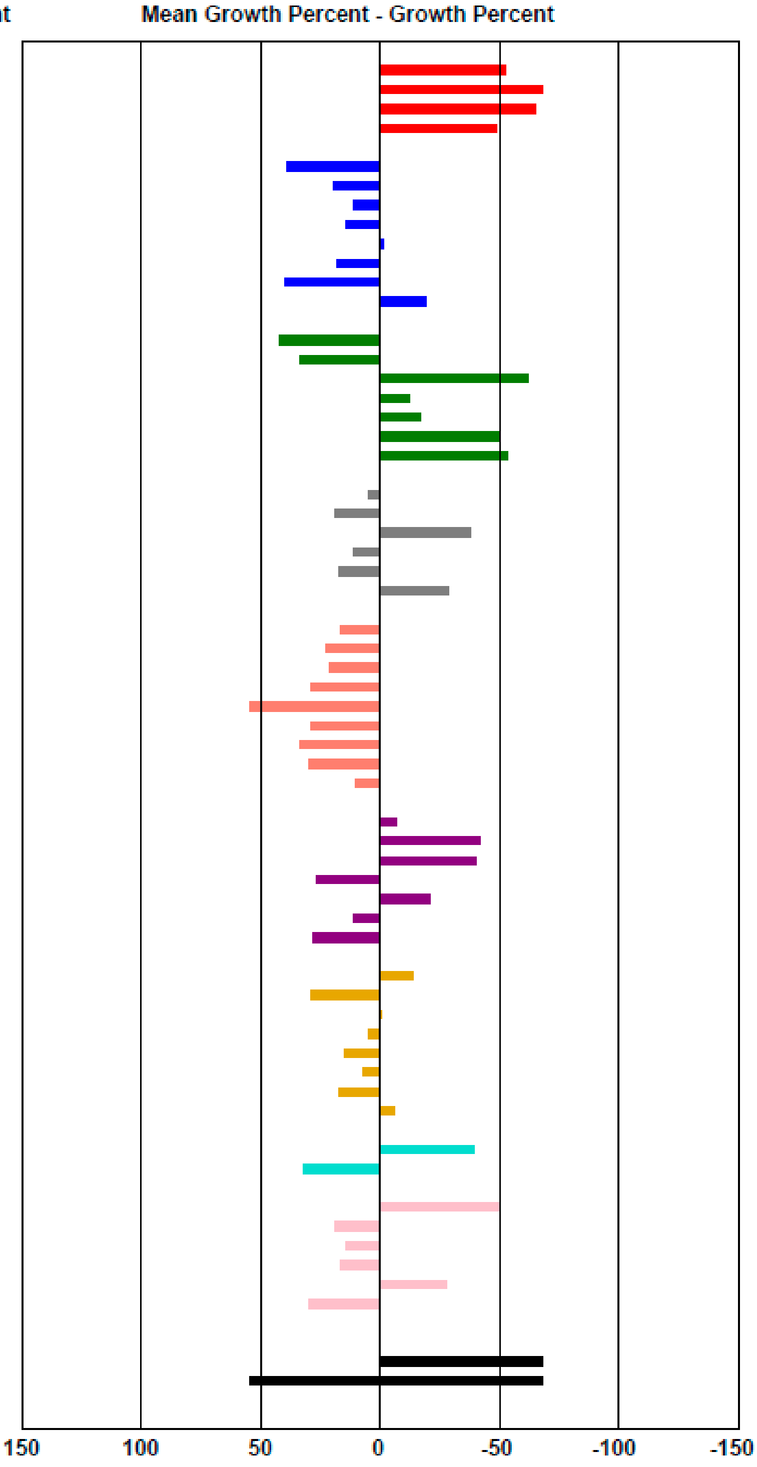

Figure S24. Protocol of anticancer screening data in concentration 10 μM for Les-4272

National Cancer Institute Developmental Therapeutics Program
In-Vitro Testing Results

NSC : D - 760917 / 1	Experiment ID : 1109NS17	Test Type : 08	Units : Molar
Report Date : February 05, 2024	Test Date : September 06, 2011	QNS :	MC :
COMI : Les-4272 (106449)	Stain Reagent : SRB Dual-Pass Related	SSPL : 0PZO	

			Log10 Concentration												
	Time		Mean Optical Densities					Percent Growth							
Panel/Cell Line	Zero	Ctrl	-8.0	-7.0	-6.0	-5.0	-4.0	-8.0	-7.0	-6.0	-5.0	-4.0	GI50	TGI	LC50
**Leukemia**															
CCRF-CEM	0.498	1.657	1.679	1.573	1.294	0.470	0.263	102	93	69	-6	-47	1.78E-6	8.38E-6	> 1.00E-4
HL-60(TB)	0.758	2.153	2.103	2.139	2.116	0.818	0.244	96	99	97	4	-68	3.22E-6	1.15E-5	5.65E-5
MOLT-4	0.679	2.076	2.084	1.997	1.913	0.598	0.229	101	94	88	-12	-66	2.41E-6	7.59E-6	5.01E-5
RPMI-8226	1.007	2.290	2.314	2.363	1.915	1.022	0.959	102	106	71	1	-5	1.99E-6	1.57E-5	> 1.00E-4
SR	0.200	0.686	0.676	0.680	0.661	0.206	0.101	98	99	95	1	-50	3.00E-6	1.05E-5	> 1.00E-4
**Non-Small Cell Lung Cancer**															
A549/ATCC	0.312	1.608	1.562	1.557	1.518	1.503	1.219	96	96	93	92	70	> 1.00E-4	> 1.00E-4	> 1.00E-4
EKVX	0.572	1.593	1.579	1.516	1.482	1.350	1.134	99	93	89	76	55	> 1.00E-4	> 1.00E-4	> 1.00E-4
HOP-62	0.435	1.083	1.053	1.065	1.059	0.942	0.440	95	97	96	78	1	2.31E-5	> 1.00E-4	> 1.00E-4
HOP-92	1.026	1.478	1.497	1.442	1.431	1.325	0.569	104	92	90	66	-45	1.40E-5	3.96E-5	> 1.00E-4
NCI-H226	0.679	1.628	1.559	1.567	1.486	1.175	0.929	93	93	85	52	26	1.22E-5	> 1.00E-4	> 1.00E-4
NCI-H23	0.483	1.302	1.258	1.252	1.157	0.668	0.457	95	94	82	23	-5	3.47E-6	6.41E-5	> 1.00E-4
NCI-H322M	0.868	1.751	1.681	1.659	1.702	1.522	1.374	92	90	95	74	57	> 1.00E-4	> 1.00E-4	> 1.00E-4
NCI-H460	0.205	1.786	1.805	1.785	1.684	1.621	1.416	101	100	94	90	77	> 1.00E-4	> 1.00E-4	> 1.00E-4
NCI-H522	0.735	1.758	1.690	1.670	1.722	1.080	0.812	93	91	97	34	8	5.50E-6	> 1.00E-4	> 1.00E-4
**Colon Cancer**															
COLO 205	0.334	1.226	1.234	1.255	1.230	1.257	0.937	101	103	100	104	68	> 1.00E-4	> 1.00E-4	> 1.00E-4
HCC-2998	0.389	1.296	1.312	1.271	1.218	1.005	1.018	102	97	91	68	69	> 1.00E-4	> 1.00E-4	> 1.00E-4
HCT-116	0.140	1.141	1.162	1.133	1.122	0.264	0.204	102	99	98	12	6	3.64E-6	> 1.00E-4	> 1.00E-4
HCT-15	0.350	1.843	1.786	1.780	1.706	0.867	0.924	96	96	91	35	38	5.32E-6	> 1.00E-4	> 1.00E-4
HT29	0.227	1.351	1.380	1.399	1.369	0.589	0.819	103	104	102	32	53	.	> 1.00E-4	> 1.00E-4
KM12	0.420	1.889	1.943	1.873	1.848	0.593	0.706	104	99	97	12	19	3.57E-6	> 1.00E-4	> 1.00E-4
SW-620	0.247	1.650	1.601	1.541	1.461	0.235	0.360	96	92	87	-5	8	2.51E-6	.	> 1.00E-4
**CNS Cancer**															
SF-268	0.451	1.414	1.411	1.390	1.375	0.962	0.765	100	97	96	53	33	1.41E-5	> 1.00E-4	> 1.00E-4
SF-295	0.583	2.039	1.936	1.986	1.835	1.300	0.650	93	96	86	49	5	9.53E-6	> 1.00E-4	> 1.00E-4
SF-539	0.670	1.803	1.809	1.769	1.877	0.935	0.988	101	97	107	23	28	4.78E-6	> 1.00E-4	> 1.00E-4
SNB-19	0.458	1.677	1.631	1.538	1.563	1.207	0.903	96	89	91	61	36	2.87E-5	> 1.00E-4	> 1.00E-4
SNB-75	0.748	1.605	1.434	1.484	1.531	1.331	0.517	80	86	91	68	-31	1.52E-5	4.87E-5	> 1.00E-4
U251	0.298	1.494	1.498	1.488	1.345	0.546	0.308	100	99	87	21	1	3.64E-6	> 1.00E-4	> 1.00E-4
**Melanoma**															
LOX IMVI	0.194	1.269	1.242	1.273	1.254	0.439	0.458	97	100	99	23	25	4.37E-6	> 1.00E-4	> 1.00E-4
MALME-3M	0.531	1.096	1.097	1.072	1.095	0.937	0.773	100	96	100	72	43	5.65E-5	> 1.00E-4	> 1.00E-4
M14	0.361	1.332	1.335	1.297	1.377	1.141	0.951	100	96	105	80	61	> 1.00E-4	> 1.00E-4	> 1.00E-4
MDA-MB-435	0.516	2.188	2.173	2.142	2.160	1.773	1.915	99	97	98	75	84	> 1.00E-4	> 1.00E-4	> 1.00E-4
SK-MEL-2	0.869	1.705	1.719	1.651	1.773	1.695	0.234	102	93	108	99	-73	1.92E-5	3.75E-5	7.34E-5
SK-MEL-28	0.441	1.228	1.199	1.199	1.121	0.951	0.717	96	96	86	65	35	3.15E-5	> 1.00E-4	> 1.00E-4
SK-MEL-5	0.295	2.253	2.110	2.189	2.009	1.670	1.661	93	97	88	70	70	> 1.00E-4	> 1.00E-4	> 1.00E-4
UACC-257	0.653	1.412	1.380	1.353	1.304	1.224	1.159	96	92	86	75	67	> 1.00E-4	> 1.00E-4	> 1.00E-4
UACC-62	0.689	2.073	2.009	2.059	2.055	1.738	0.864	95	99	99	76	13	2.56E-5	> 1.00E-4	> 1.00E-4
**Ovarian Cancer**															
IGROV1	0.456	1.523	1.521	1.546	1.496	0.977	0.668	100	102	97	49	20	9.43E-6	> 1.00E-4	> 1.00E-4
OVCAR-3	0.552	1.474	1.516	1.501	1.396	0.712	0.715	105	103	92	17	18	3.63E-6	> 1.00E-4	> 1.00E-4
OVCAR-4	0.420	0.971	1.014	0.911	0.868	0.495	0.441	108	89	81	14	4	2.90E-6	> 1.00E-4	> 1.00E-4
OVCAR-5	0.527	1.421	1.361	1.385	1.399	1.299	1.127	93	96	97	86	67	> 1.00E-4	> 1.00E-4	> 1.00E-4
OVCAR-8	0.376	1.568	1.565	1.591	1.408	0.636	0.636	100	102	87	22	22	3.67E-6	> 1.00E-4	> 1.00E-4
NCI/ADR-RES	0.451	1.580	1.572	1.547	1.479	1.005	0.991	99	97	91	49	48	9.49E-6	> 1.00E-4	> 1.00E-4
SK-OV-3	0.356	0.863	0.858	0.851	0.882	0.819	0.401	99	98	104	91	9	3.17E-5	> 1.00E-4	> 1.00E-4
**Renal Cancer**															
786-0	0.457	1.819	1.733	1.797	1.855	0.741	0.573	94	98	103	21	9	4.40E-6	> 1.00E-4	> 1.00E-4
A498	1.040	1.549	1.264	1.259	1.309	1.363	0.751	44	43	53	63	-28	.	4.95E-5	> 1.00E-4
ACHN	0.403	1.637	1.685	1.661	1.694	1.197	0.662	104	102	105	64	21	2.14E-5	> 1.00E-4	> 1.00E-4
CAKI-1	0.595	2.023	1.996	2.004	1.862	1.230	1.356	98	99	89	44	53	.	> 1.00E-4	> 1.00E-4
RXF 393	0.550	1.086	1.073	1.076	0.986	0.426	0.160	98	98	81	-23	-71	2.00E-6	6.06E-6	3.68E-5
SN12C	0.449	1.646	1.547	1.642	1.638	1.091	1.059	92	100	99	54	51	> 1.00E-4	> 1.00E-4	> 1.00E-4
TK-10	0.708	1.509	1.445	1.532	1.549	1.013	0.474	92	103	105	38	-33	6.64E-6	3.43E-5	> 1.00E-4
UO-31	0.540	1.462	1.388	1.360	1.342	0.854	1.040	92	89	87	34	54	.	> 1.00E-4	> 1.00E-4
**Prostate Cancer**															
PC-3	0.483	1.240	1.201	1.193	1.085	0.639	0.658	95	94	79	21	23	3.16E-6	> 1.00E-4	> 1.00E-4
DU-145	0.333	1.242	1.291	1.282	1.285	1.094	0.813	105	104	105	84	53	> 1.00E-4	> 1.00E-4	> 1.00E-4
**Breast Cancer**															
MCF7	0.250	1.192	1.118	1.155	1.060	0.303	0.313	92	96	86	6	7	2.80E-6	> 1.00E-4	> 1.00E-4
MDA-MB-231/ATCC	0.637	1.628	1.681	1.649	1.646	1.480	0.695	105	102	102	85	6	2.77E-5	> 1.00E-4	> 1.00E-4
HS 578T	0.814	1.551	1.574	1.496	1.389	1.166	0.796	103	92	78	48	-2	8.43E-6	9.03E-5	> 1.00E-4
BT-549	0.679	1.565	1.562	1.543	1.575	1.353	0.968	100	98	101	76	33	3.98E-5	> 1.00E-4	> 1.00E-4
T-47D	0.525	1.056	1.038	1.058	1.050	0.643	0.671	97	100	99	22	27	4.33E-6	> 1.00E-4	> 1.00E-4
MDA-MB-468	0.608	1.215	1.158	1.159	1.106	0.835	0.695	91	91	82	37	14	5.21E-6	> 1.00E-4	> 1.00E-4

Figure S25. Protocol of in-depth anticancer screening data of Les-4272 at concentrations ranging from 10^{-4} to 10^{-8} M.

Developmental Therapeutics Program One Dose Mean Graph	NSC: D-760926 / 1	Conc: 1.00E-5 Molar	Test Date: Aug 08, 2011
	Experiment ID: 1108OS00		Report Date: Feb 05, 2024

Panel/Cell Line	Growth Percent
Leukemia	
CCRF-CEM	58.58
HL-60(TB)	47.39
MOLT-4	71.57
RPMI-8226	59.17
Non-Small Cell Lung Cancer	
A549/ATCC	53.80
EKVX	35.63
HOP-62	69.20
NCI-H226	73.43
NCI-H23	39.41
NCI-H322M	91.49
NCI-H460	43.33
NCI-H522	38.62
Colon Cancer	
COLO 205	55.66
HCC-2998	80.36
HCT-116	39.48
HCT-15	52.77
HT29	24.85
KM12	39.52
SW-620	47.65
CNS Cancer	
SF-268	87.29
SF-295	43.32
SF-539	79.46
SNB-19	79.23
U251	64.80
Melanoma	
LOX IMVI	70.26
MALME-3M	48.97
M14	58.84
MDA-MB-435	0.24
SK-MEL-2	76.43
SK-MEL-28	66.31
SK-MEL-5	46.35
UACC-257	98.02
UACC-62	54.48
Ovarian Cancer	
IGROV1	56.58
OVCAR-3	34.32
OVCAR-4	59.16
OVCAR-5	85.91
OVCAR-8	80.01
NCI/ADR-RES	37.98
SK-OV-3	74.36
Renal Cancer	
786-0	91.97
A498	57.04
ACHN	78.39
CAKI-1	49.13
RXF 393	59.78
SN12C	86.84
TK-10	101.41
UO-31	69.68
Prostate Cancer	
PC-3	59.11
DU-145	106.55
Breast Cancer	
MCF7	17.65
MDA-MB-231/ATCC	80.84
HS 578T	83.51
BT-549	85.41
T-47D	46.02
MDA-MB-468	41.09
Mean	61.40
Delta	61.16
Range	106.31

Mean Growth Percent - Growth Percent

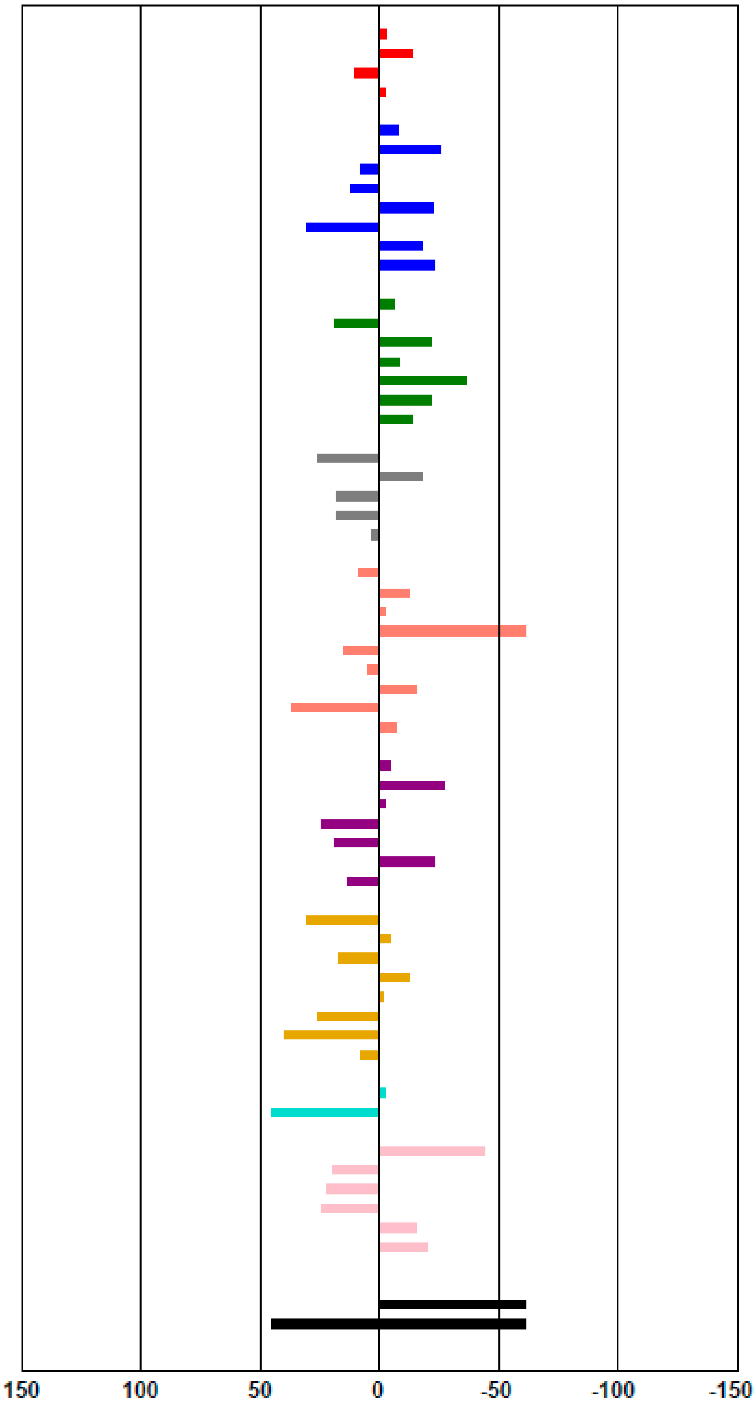

Figure S26. Protocol of anticancer screening data in concentration 10 µM for Les-4309

National Cancer Institute Developmental Therapeutics Program
In-Vitro Testing Results

NSC : D - 760926 / 1	Experiment ID : 1109NS17	Test Type : 08	Units : Molar
Report Date : February 05, 2024	Test Date : September 06, 2011	QNS :	MC :
COMI : Les-4309 (108530)	Stain Reagent : SRB Dual-Pass Related	SSPL : 0PZO	

			Log10 Concentration													
	Time		Mean Optical Densities					Percent Growth								
Panel/Cell Line	Zero	Ctrl	-8.0	-7.0	-6.0	-5.0	-4.0	-8.0	-7.0	-6.0	-5.0	-4.0	GI50	TGI	LC50
**Leukemia**															
CCRF-CEM	0.498	1.694	1.681	1.616	1.597	0.951	1.069	99	93	92	38	48	5.96E-6	> 1.00E-4	> 1.00E-4
HL-60(TB)	0.758	2.282	1.992	2.123	2.056	1.291	1.150	81	90	85	35	26	5.01E-6	> 1.00E-4	> 1.00E-4
MOLT-4	0.679	2.087	2.119	2.070	2.085	1.422	1.458	102	99	100	53	55	> 1.00E-4	> 1.00E-4	> 1.00E-4
RPMI-8226	1.007	2.389	2.389	2.325	2.149	1.539	1.430	100	95	83	39	31	5.49E-6	> 1.00E-4	> 1.00E-4
SR	0.200	0.723	0.717	0.723	0.643	0.271	0.262	99	100	85	13	12	3.08E-6	> 1.00E-4	> 1.00E-4
**Non-Small Cell Lung Cancer**															
A549/ATCC	0.312	1.607	1.590	1.621	1.488	0.861	0.866	99	101	91	42	43	6.96E-6	> 1.00E-4	> 1.00E-4
EKVX	0.572	1.570	1.574	1.497	1.402	1.005	1.006	100	93	83	43	43	6.81E-6	> 1.00E-4	> 1.00E-4
HOP-62	0.435	1.109	1.078	1.055	1.067	0.851	0.772	95	92	94	62	50	> 1.00E-4	> 1.00E-4	> 1.00E-4
HOP-92	1.026	1.498	1.446	1.443	1.395	1.301	1.292	89	88	78	58	56	> 1.00E-4	> 1.00E-4	> 1.00E-4
NCI-H226	0.679	1.611	1.563	1.570	1.522	1.291	1.330	95	96	90	66	70	> 1.00E-4	> 1.00E-4	> 1.00E-4
NCI-H23	0.483	1.314	1.264	1.249	1.117	0.898	0.919	94	92	76	50	52	.	> 1.00E-4	> 1.00E-4
NCI-H322M	0.868	1.760	1.680	1.683	1.665	1.452	1.448	91	91	89	65	65	> 1.00E-4	> 1.00E-4	> 1.00E-4
NCI-H460	0.205	1.838	1.803	1.843	1.739	0.633	0.577	98	100	94	26	23	4.45E-6	> 1.00E-4	> 1.00E-4
NCI-H522	0.735	1.766	1.648	1.674	1.625	0.995	0.987	88	91	86	25	24	3.92E-6	> 1.00E-4	> 1.00E-4
**Colon Cancer**															
COLO 205	0.334	1.293	1.280	1.287	1.285	0.748	0.679	99	99	99	43	36	7.54E-6	> 1.00E-4	> 1.00E-4
HCC-2998	0.389	1.307	1.323	1.291	1.190	0.943	0.932	102	98	87	60	59	> 1.00E-4	> 1.00E-4	> 1.00E-4
HCT-116	0.140	1.191	1.207	1.168	1.188	0.589	0.547	102	98	100	43	39	7.46E-6	> 1.00E-4	> 1.00E-4
HCT-15	0.350	1.767	1.774	1.700	1.684	1.037	0.938	101	95	94	48	42	9.27E-6	> 1.00E-4	> 1.00E-4
HT29	0.227	1.401	1.422	1.471	1.437	0.528	0.504	102	106	103	26	24	4.85E-6	> 1.00E-4	> 1.00E-4
KM12	0.420	1.960	1.919	1.806	1.595	0.752	0.744	97	90	76	22	21	3.02E-6	> 1.00E-4	> 1.00E-4
SW-620	0.247	1.672	1.599	1.575	1.462	0.709	0.702	95	93	85	32	32	4.65E-6	> 1.00E-4	> 1.00E-4
**CNS Cancer**															
SF-268	0.451	1.411	1.309	1.346	1.319	1.025	1.006	89	93	90	60	58	> 1.00E-4	> 1.00E-4	> 1.00E-4
SF-295	0.583	2.046	1.930	1.977	1.880	1.086	1.092	92	95	89	34	35	5.15E-6	> 1.00E-4	> 1.00E-4
SF-539	0.670	1.873	1.901	1.840	1.941	1.553	1.412	102	97	106	73	62	> 1.00E-4	> 1.00E-4	> 1.00E-4
SNB-19	0.458	1.730	1.627	1.662	1.703	1.342	1.303	92	95	98	70	66	> 1.00E-4	> 1.00E-4	> 1.00E-4
SNB-75	0.748	1.751	1.604	1.523	1.452	1.052	1.060	85	77	70	30	31	3.20E-6	> 1.00E-4	> 1.00E-4
U251	0.298	1.508	1.491	1.484	1.400	0.933	0.972	99	98	91	53	56	> 1.00E-4	> 1.00E-4	> 1.00E-4
**Melanoma**															
LOX IMVI	0.194	1.261	1.280	1.181	1.198	0.794	0.813	102	92	94	56	58	> 1.00E-4	> 1.00E-4	> 1.00E-4
MALME-3M	0.531	1.085	1.101	1.070	1.063	0.763	0.746	103	97	96	42	39	7.07E-6	> 1.00E-4	> 1.00E-4
M14	0.361	1.368	1.307	1.316	1.333	0.769	0.792	94	95	97	41	43	6.78E-6	> 1.00E-4	> 1.00E-4
MDA-MB-435	0.516	2.203	2.158	2.108	1.799	0.483	0.496	97	94	76	-6	-4	2.07E-6	8.36E-6	> 1.00E-4
SK-MEL-2	0.869	1.684	1.613	1.716	1.707	1.180	1.198	91	104	103	38	40	6.57E-6	> 1.00E-4	> 1.00E-4
SK-MEL-28	0.441	1.361	1.397	1.339	1.216	0.928	0.931	104	98	84	53	53	> 1.00E-4	> 1.00E-4	> 1.00E-4
SK-MEL-5	0.295	2.344	2.335	2.259	2.271	1.112	0.958	100	96	96	40	32	6.62E-6	> 1.00E-4	> 1.00E-4
UACC-257	0.653	1.429	1.366	1.416	1.316	1.124	1.141	92	98	85	61	63	> 1.00E-4	> 1.00E-4	> 1.00E-4
UACC-62	0.689	2.106	2.042	1.988	2.053	1.434	1.426	95	92	96	53	52	> 1.00E-4	> 1.00E-4	> 1.00E-4
**Ovarian Cancer**															
IGROV1	0.456	1.462	1.497	1.411	1.346	0.952	0.872	103	95	89	49	41	9.61E-6	> 1.00E-4	> 1.00E-4
OVCAR-3	0.552	1.473	1.490	1.420	1.476	0.690	0.670	102	94	100	15	13	3.89E-6	> 1.00E-4	> 1.00E-4
OVCAR-4	0.420	1.074	1.098	1.016	0.897	0.739	0.721	104	91	73	49	46	8.92E-6	> 1.00E-4	> 1.00E-4
OVCAR-5	0.527	1.401	1.381	1.360	1.352	1.196	1.114	98	95	94	77	67	> 1.00E-4	> 1.00E-4	> 1.00E-4
OVCAR-8	0.376	1.626	1.616	1.628	1.598	1.266	1.324	99	100	98	71	76	> 1.00E-4	> 1.00E-4	> 1.00E-4
NCI/ADR-RES	0.451	1.561	1.546	1.542	1.363	0.664	0.712	99	98	82	19	24	3.24E-6	> 1.00E-4	> 1.00E-4
SK-OV-3	0.356	0.877	0.864	0.879	0.862	0.679	0.640	97	100	97	62	54	> 1.00E-4	> 1.00E-4	> 1.00E-4
**Renal Cancer**															
786-0	0.457	1.781	1.741	1.795	1.706	1.377	1.333	97	101	94	69	66	> 1.00E-4	> 1.00E-4	> 1.00E-4
A498	1.040	1.548	1.417	1.386	1.335	1.186	1.112	74	68	58	29	14	1.87E-6	> 1.00E-4	> 1.00E-4
ACHN	0.403	1.579	1.616	1.611	1.687	1.275	1.273	103	103	109	74	74	> 1.00E-4	> 1.00E-4	> 1.00E-4
CAKI-1	0.595	2.015	1.966	1.926	1.750	1.202	1.191	97	94	81	43	42	6.48E-6	> 1.00E-4	> 1.00E-4
RXF 393	0.550	1.153	1.186	1.167	1.114	0.883	0.878	105	102	93	55	54	> 1.00E-4	> 1.00E-4	> 1.00E-4
SN12C	0.449	1.666	1.592	1.646	1.661	1.358	1.363	94	98	100	75	75	> 1.00E-4	> 1.00E-4	> 1.00E-4
TK-10	0.708	1.511	1.549	1.588	1.604	1.395	1.371	105	110	112	86	83	> 1.00E-4	> 1.00E-4	> 1.00E-4
UO-31	0.540	1.416	1.341	1.359	1.315	1.054	1.058	91	93	88	59	59	> 1.00E-4	> 1.00E-4	> 1.00E-4
**Prostate Cancer**															
PC-3	0.483	1.249	1.206	1.168	1.148	0.908	0.888	94	89	87	55	53	> 1.00E-4	> 1.00E-4	> 1.00E-4
DU-145	0.333	1.228	1.247	1.238	1.248	0.964	0.911	102	101	102	71	65	> 1.00E-4	> 1.00E-4	> 1.00E-4
**Breast Cancer**															
MCF7	0.250	1.399	1.348	1.312	0.918	0.442	0.428	96	92	58	17	15	1.57E-6	> 1.00E-4	> 1.00E-4
MDA-MB-231/ATCC	0.637	1.596	1.625	1.538	1.604	1.430	1.387	103	94	101	83	78	> 1.00E-4	> 1.00E-4	> 1.00E-4
HS 578T	0.814	1.595	1.544	1.553	1.443	1.178	1.086	93	95	81	47	35	7.94E-6	> 1.00E-4	> 1.00E-4
BT-549	0.679	1.537	1.526	1.533	1.598	1.276	1.280	99	100	107	70	70	> 1.00E-4	> 1.00E-4	> 1.00E-4
T-47D	0.525	1.083	1.126	1.057	0.976	0.790	0.739	108	95	81	47	38	8.37E-6	> 1.00E-4	> 1.00E-4
MDA-MB-468	0.608	1.246	1.224	1.201	1.181	0.734	0.729	97	93	90	20	19	3.70E-6	> 1.00E-4	> 1.00E-4

Figure S27. Protocol of in-depth anticancer screening data of Les-4309 at concentrations ranging from 10^{-4} to 10^{-8} M.

Developmental Therapeutics Program One Dose Mean Graph	NSC: D-771871 / 1	Conc: 1.00E-5 Molar	Test Date: Jan 14, 2013
	Experiment ID: 1301OS10		Report Date: Feb 05, 2024

Panel/Cell Line	Growth Percent
**Leukemia**	
CCRF-CEM	-57.28
HL-60(TB)	43.71
K-562	28.98
MOLT-4	6.82
RPMI-8226	36.12
SR	-46.36
**Non-Small Cell Lung Cancer**	
A549/ATCC	31.72
HOP-62	95.88
NCI-H226	72.16
NCI-H23	79.37
NCI-H322M	71.64
NCI-H460	49.79
NCI-H522	-84.06
**Colon Cancer**	
COLO 205	66.66
HCC-2998	90.41
HCT-116	44.10
HCT-15	45.72
HT29	37.37
KM12	52.01
SW-620	77.91
**CNS Cancer**	
SF-268	60.38
SF-295	39.29
SF-539	80.82
SNB-19	65.38
SNB-75	58.77
U251	56.76
**Melanoma**	
LOX IMVI	-73.36
MALME-3M	-14.23
M14	61.21
MDA-MB-435	52.87
SK-MEL-2	59.28
SK-MEL-28	73.36
SK-MEL-5	65.91
UACC-257	66.08
UACC-62	70.82
**Ovarian Cancer**	
IGROV1	65.47
OVCAR-3	65.69
OVCAR-4	63.43
OVCAR-5	84.05
OVCAR-8	58.36
NCI/ADR-RES	52.35
SK-OV-3	95.75
**Renal Cancer**	
786-0	73.80
A498	68.56
ACHN	43.37
CAKI-1	56.34
RXF 393	58.58
SN12C	64.79
TK-10	60.32
UO-31	29.10
**Prostate Cancer**	
PC-3	18.93
DU-145	75.84
**Breast Cancer**	
MCF7	26.76
MDA-MB-231/ATCC	84.42
HS 578T	69.97
BT-549	66.11
T-47D	60.59
MDA-MB-468	52.88
Mean	49.34
Delta	133.40
Range	179.94

Mean Growth Percent - Growth Percent

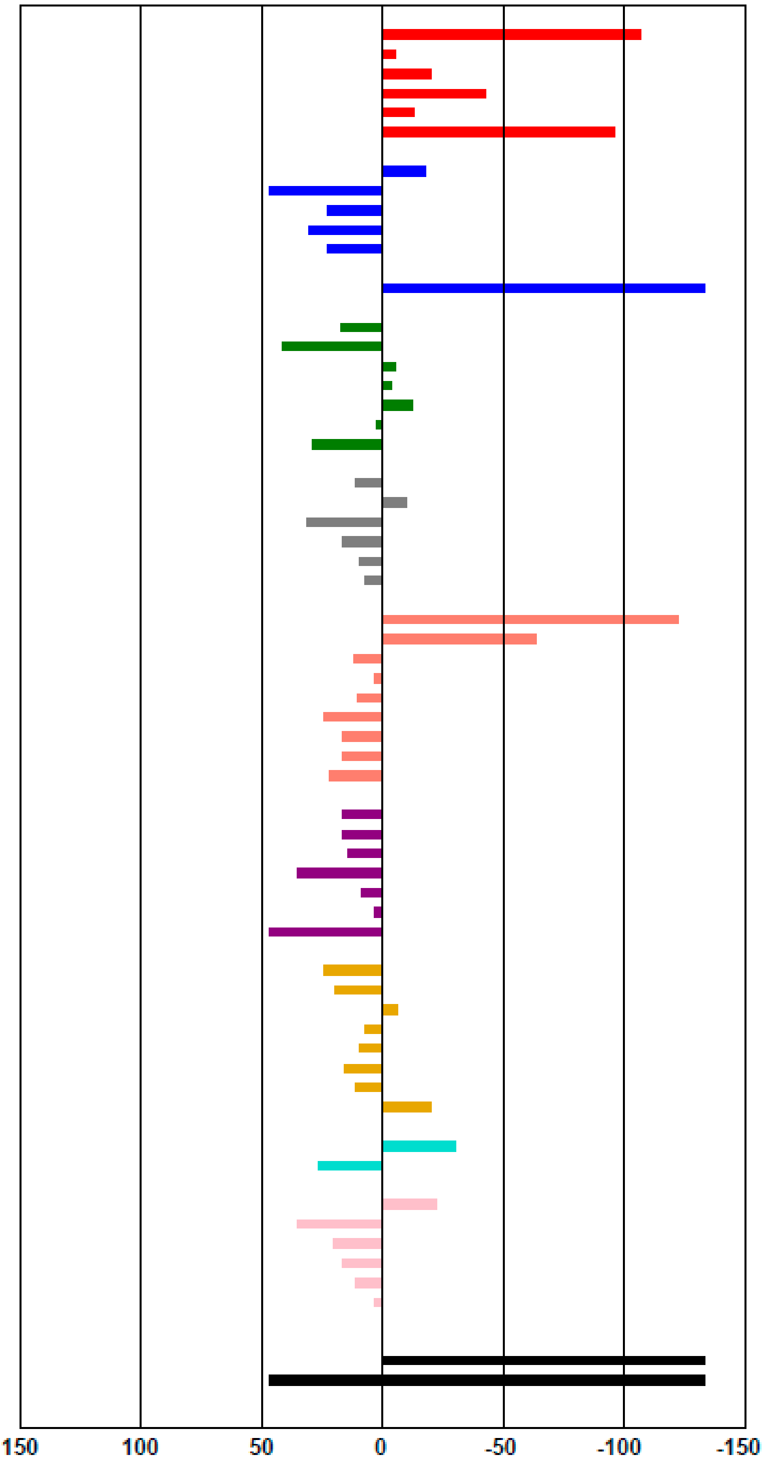

Figure S28. Protocol of anticancer screening data in concentration 10 µM for Les-4403

National Cancer Institute Developmental Therapeutics Program
In-Vitro Testing Results

NSC : D - 771871 / 1	Experiment ID : 1304NS55	Test Type : 08	Units : Molar
Report Date : February 05, 2024	Test Date : April 08, 2013	QNS :	MC :
COMI : Les-4403 (115231)	Stain Reagent : SRB Dual-Pass Related	SSPL : 0PZO	

			Log10 Concentration												
	Time		Mean Optical Densities					Percent Growth							
Panel/Cell Line	Zero	Ctrl	-8.0	-7.0	-6.0	-5.0	-4.0	-8.0	-7.0	-6.0	-5.0	-4.0	GI50	TGI	LC50
Leukemia															
CCRF-CEM	0.520	2.655	2.630	2.646	2.501	0.291	0.302	99	100	93	-44	-42	2.05E-6	4.76E-6	> 1.00E-4
HL-60(TB)	1.055	2.774	2.861	2.939	2.860	1.680	1.455	105	110	105	36	23	6.33E-6	> 1.00E-4	> 1.00E-4
K-562	0.205	1.808	1.792	1.758	1.592	0.533	0.482	99	97	86	20	17	3.57E-6	> 1.00E-4	> 1.00E-4
MOLT-4	0.758	2.710	2.756	2.831	2.482	0.925	0.627	102	106	88	9	-17	3.02E-6	2.14E-5	> 1.00E-4
RPMI-8226	0.779	2.525	2.572	2.579	2.557	1.254	1.046	103	103	102	27	15	4.94E-6	> 1.00E-4	> 1.00E-4
SR	0.524	2.667	2.616	2.604	2.560	0.668	0.573	98	97	95	7	2	3.23E-6	> 1.00E-4	> 1.00E-4
Non-Small Cell Lung Cancer															
A549/ATCC	0.519	2.017	2.014	1.942	1.753	0.882	0.793	100	95	82	24	18	3.60E-6	> 1.00E-4	> 1.00E-4
HOP-62	0.689	2.121	2.151	2.091	1.948	1.745	1.527	102	98	88	74	59	> 1.00E-4	> 1.00E-4	> 1.00E-4
NCI-H226	1.253	2.535	2.467	2.433	2.372	1.923	1.838	95	92	87	52	46	2.18E-5	> 1.00E-4	> 1.00E-4
NCI-H322M	0.599	1.549	1.515	1.541	1.446	1.159	1.046	96	99	89	59	47	5.64E-5	> 1.00E-4	> 1.00E-4
NCI-H460	0.340	2.990	3.001	2.927	2.835	1.437	1.177	100	98	94	41	32	6.87E-6	> 1.00E-4	> 1.00E-4
NCI-H522	1.247	2.328	2.323	2.305	2.077	0.238	0.288	99	98	77	-81	-77	1.48E-6	3.07E-6	6.37E-6
Colon Cancer															
COLO 205	0.470	2.008	2.179	2.039	2.098	1.283	1.126	111	102	106	53	43	1.90E-5	> 1.00E-4	> 1.00E-4
HCC-2998	0.528	1.752	1.697	1.790	1.763	1.339	1.246	95	103	101	66	59	> 1.00E-4	> 1.00E-4	> 1.00E-4
HCT-116	0.264	2.291	2.317	2.370	2.161	0.970	0.846	101	104	94	35	29	5.51E-6	> 1.00E-4	> 1.00E-4
HCT-15	0.331	2.480	2.441	2.352	2.266	1.102	0.964	98	94	90	36	29	5.48E-6	> 1.00E-4	> 1.00E-4
HT29	0.260	1.256	1.272	1.321	1.303	0.635	0.538	102	107	105	38	28	6.55E-6	> 1.00E-4	> 1.00E-4
KM12	0.665	3.082	3.175	3.192	3.072	1.542	1.505	104	105	100	36	35	6.07E-6	> 1.00E-4	> 1.00E-4
SW-620	0.408	2.620	2.532	2.499	2.389	1.602	1.339	96	95	90	54	42	2.15E-5	> 1.00E-4	> 1.00E-4
CNS Cancer															
SF-268	0.612	2.060	2.065	2.068	2.010	1.364	1.282	100	101	96	52	46	2.16E-5	> 1.00E-4	> 1.00E-4
SF-295	0.899	2.504	2.365	2.336	2.289	1.152	1.022	91	90	87	16	8	3.29E-6	> 1.00E-4	> 1.00E-4
SF-539	0.979	2.802	2.786	2.691	2.752	2.271	2.134	99	94	97	71	63	> 1.00E-4	> 1.00E-4	> 1.00E-4
SNB-19	0.755	2.241	2.157	2.093	1.987	1.469	1.450	94	90	83	48	47	8.79E-6	> 1.00E-4	> 1.00E-4
SNB-75	0.959	1.885	1.737	1.708	1.687	1.403	1.084	84	81	79	48	13	8.57E-6	> 1.00E-4	> 1.00E-4
U251	0.585	2.076	1.984	1.911	1.832	1.127	1.118	94	89	84	36	36	5.14E-6	> 1.00E-4	> 1.00E-4
Melanoma															
LOX IMVI	0.658	2.995	3.026	3.016	2.985	0.517	0.537	101	101	100	-22	-18	2.57E-6	6.64E-6	> 1.00E-4
M14	0.546	2.130	2.154	2.205	2.163	1.348	1.015	102	105	102	51	30	1.07E-5	> 1.00E-4	> 1.00E-4
MDA-MB-435	0.544	2.162	2.118	2.045	1.987	1.181	1.054	97	93	89	39	32	6.12E-6	> 1.00E-4	> 1.00E-4
SK-MEL-2	0.753	1.198	1.208	1.242	1.224	1.025	0.870	102	110	106	61	26	2.08E-5	> 1.00E-4	> 1.00E-4
SK-MEL-28	0.592	1.574	1.562	1.525	1.524	1.200	1.137	99	95	95	62	55	> 1.00E-4	> 1.00E-4	> 1.00E-4
SK-MEL-5	0.929	2.832	2.681	2.715	2.562	1.233	0.936	92	94	86	16	0	3.25E-6	> 1.00E-4	> 1.00E-4
UACC-257	0.905	1.651	1.557	1.542	1.551	1.191	1.159	87	85	86	38	34	5.72E-6	> 1.00E-4	> 1.00E-4
UACC-62	0.902	2.753	2.768	2.667	2.569	1.935	1.799	101	95	90	56	48	6.14E-5	> 1.00E-4	> 1.00E-4
Ovarian Cancer															
IGROV1	0.486	1.926	1.982	1.978	1.857	1.191	0.987	104	104	95	49	35	9.48E-6	> 1.00E-4	> 1.00E-4
OVCAR-3	0.705	2.047	2.108	2.116	2.057	1.345	1.241	105	105	101	48	40	9.05E-6	> 1.00E-4	> 1.00E-4
OVCAR-4	0.559	1.125	1.120	1.065	1.082	0.818	0.779	99	89	92	46	39	8.08E-6	> 1.00E-4	> 1.00E-4
OVCAR-5	0.593	1.453	1.448	1.441	1.387	1.268	1.190	99	99	92	79	69	> 1.00E-4	> 1.00E-4	> 1.00E-4
OVCAR-8	0.588	2.092	2.046	1.968	1.984	1.284	1.277	97	92	93	46	46	8.32E-6	> 1.00E-4	> 1.00E-4
NCI/ADR-RES	0.543	2.025	2.155	2.159	2.036	1.234	1.100	109	109	101	47	38	8.65E-6	> 1.00E-4	> 1.00E-4
SK-OV-3	0.584	1.492	1.548	1.535	1.580	1.412	1.203	106	105	110	91	68	> 1.00E-4	> 1.00E-4	> 1.00E-4
Renal Cancer															
786-0	0.584	2.470	2.496	2.555	2.529	1.784	1.553	101	105	103	64	51	> 1.00E-4	> 1.00E-4	> 1.00E-4
A498	1.700	2.647	2.537	2.473	2.349	2.047	1.923	88	82	69	37	24	3.81E-6	> 1.00E-4	> 1.00E-4
ACHN	0.411	2.025	2.054	2.043	1.964	0.986	0.925	102	101	96	36	32	5.78E-6	> 1.00E-4	> 1.00E-4
CAKI-1	0.648	2.332	2.327	2.350	2.236	1.373	1.180	100	101	94	43	32	7.31E-6	> 1.00E-4	> 1.00E-4
RXF 393	0.948	1.782	1.771	1.716	1.520	1.289	1.165	99	92	69	41	26	4.68E-6	> 1.00E-4	> 1.00E-4
SN12C	0.643	2.516	2.484	2.432	2.410	1.577	1.356	98	95	94	50	38	9.92E-6	> 1.00E-4	> 1.00E-4
UO-31	0.655	1.941	1.791	1.814	1.601	0.822	0.734	88	90	74	13	6	2.45E-6	> 1.00E-4	> 1.00E-4
Prostate Cancer															
PC-3	0.698	2.230	2.138	2.121	1.950	1.037	1.035	94	93	82	22	22	3.40E-6	> 1.00E-4	> 1.00E-4
DU-145	0.460	1.769	1.827	1.865	1.823	1.283	1.174	104	107	104	63	55	> 1.00E-4	> 1.00E-4	> 1.00E-4
Breast Cancer															
MCF7	0.419	2.341	2.271	2.165	2.076	0.812	0.753	96	91	86	20	17	3.55E-6	> 1.00E-4	> 1.00E-4
MDA-MB-231/ATCC	0.681	1.526	1.565	1.513	1.443	1.125	0.887	105	98	90	52	24	1.23E-5	> 1.00E-4	> 1.00E-4
HS 578T	1.268	2.213	2.111	2.102	2.015	1.772	1.515	89	88	79	53	26	1.32E-5	> 1.00E-4	> 1.00E-4
BT-549	1.240	2.396	2.434	2.497	2.415	2.003	1.636	103	109	102	66	34	3.19E-5	> 1.00E-4	> 1.00E-4
MDA-MB-468	0.767	1.659	1.618	1.563	1.544	1.181	0.992	95	89	87	46	25	8.16E-6	> 1.00E-4	> 1.00E-4

Figure S29. Protocol of in-depth anticancer screening data of Les-4403 at concentrations ranging from 10^{-4} to 10^{-8} M.

Table S1. SwissTarget similarity prediction scores for the tubulin inhibitors

**Inhibitor**	**Tubulin-Ligand Probability Score**	**Best Target Probability score**	**CHEMBL Target ID**
Les-1419	none	Kinesin-like protein 1	CHEMBL4581
Les-4272	none	Bromodomain-containing protein 2 (0.12)	CHEMBL1293289
Les-4309	none	Prolyl endopeptidase (0.11)	CHEMBL3202
Les-4403	none	p53-Binding protein Mdm-2 (0.12)	CHEMBL5023
Les-6370	none	Protein-glutamine gammaglutamyltransferase	CHEMBL2730

Table S2. SMILES of the virtual hit compounds

Compound	SMILES
Les-4272	O=C(CN1C(C(C2=C1C=CC(Cl)=C2)=O)=O)N3N=C(C4=CC5=CC=CC=C5C=C4)CC3C6=CC=C(OC)C=C6
Les-4274	O=C(CSC1=NC(C=CC=C2)=C2O1)N3C(CC(C4=CC5=CC=CC=C5C=C4)=N3)C6=CC=C(OC)C=C6
Les-4309	O=C(CSC1=NC(C=CC=C2)=C2O1)N3C(CC(C4=CC5=CC=CC=C5C=C4)=N3)C6=CC=C(F)C=C6
Les-5463	O=C1N(C2=CC=CN=C2)C(S/C1=C\N3N=C(C4=CC=C5C=CC=CC5=C4)CC3C6=CC=CC=C6O)=S
Les-4403	ClC(C=C1)=CC=C1C2CC(C3=CC4=CC=CC=C4C=C3)=NN2C(CSC5=NN=C(CC6=CC=CC=C6)N5)=O
Les-3639	CC1=CC2=C(NC(/C2=C3C(N=C(S/3)N4N=C(CC4C(C=C5)=CC=C5Cl)C6=CC=CC=C6)=O)=O)C=C1
Les-3643	O=C(NC1=C/2C=CC=C1)C2=C3C(N=C(S\3)N4N=C(CC4C(C=C5)=CC=C5Cl)C6=CC=CC=C6)=O
Les-3645	O=C(N(C(C)=O)C1=C/2C=CC=C1)C2=C3C(N=C(S\3)N4N=C(CC4C(C=C5)=CC=C5Cl)C6=CC=CC=C6)=O
Les-3755	O=C(NC1=C/2C=CC=C1)C2=C3C(N=C(S\3)N4N=C(CC4C(C=C5)=CC=C5Cl)C6=CC(C=CC=C7)=C7C=C6)=O
Les-3833	BrC1=CC2=C(NC(/C2=C3C(N=C(S/3)N4N=C(CC4C(C=C5)=CC=C5OC)C6=CC(C=CC=C7)=C7C=C6)=O)=O)C=C1
Les-3882	O=C(NC1=C/2C=CC=C1)C2=C3C(N=C(S\3)N4N=C(CC4C(C=C5)=CC=C5OC)C6=CC(C=CC=C7)=C7C=C6)=O
Les-1127	[O-][N+](C1=CC(C(C2C(C(N(C2=O)C3=CC=C(Br)C=C3)=O)S4)C(S5)=C4NC5=O)=C(OCC6=CC=CC=C6Cl)C=C1)=O
Les-1419	ClC1=CC=CC=C1/C=C2SC(N=C\2N3C(C4=CC=CC=C4O)CC(C5=CC=CC=C5)=N3)=O
Les-1458	O=C(S/C1=C\C=C\C2=CC=CC=C2)N(CCC(NC3=CC(C(F)(F)F)=CC=C3)=O)C1=O
Les-5552	S=C(N(NC(CC1=CC=CC=C1NC2=C(Cl)C=CC=C2Cl)=O)C/3=O)SC3=C/N4N=C(C5=CC6=CC=CC=C6C=C5)CC4C7=CC=C(OC)C=C7
Les-6368	O=C(CN1C(SC2=C1SCC3(C)COC4=C(C23)C=CC=C4)=O)N5N=C(CC5C6=CC7=C(C=CC=C7)C=C6)C(C=C8)=CC=C8OC
Les-6370	O=C(CN1C(SC2=C1SCC3COC4=C(C23)C=CC=C4)=O)N5N=C(CC5C6=CC7=C(C=CC=C7)C=C6)C(C=C8)=CC=C8OC
Les-3166	S=C(N(NC1=NC2=C(C=CC=C2)S1)C/3=O)SC3=C/C4=C(OCC(NC5=CC=C(OC)C=C5)=O)C=CC(Cl)=C4

Table S3. Raw data of the obtained values from ELISA assessment of β-tubulin concentration in pg/mL

N of probe / Name	Control	Les-1419 1.0 µM	Les-1419 5 µM	Les-1419 10.0 µM	Les-4309 1.0 µM	Les-4309 5 µM	Les-4309 10.0 µM	Comb. (10 nM)
**1**	1731	982	807	725	1810	1412	834	214
**2**	1575	1109	840	755	1918	1466	717	187
**3**	1519	1030	816	559	1723	1725	653	538
**Means + SD**	1608 ± 109.9	1040 ± 64.13	821 ± 17.06	679.7 ± 105.6	1817 ± 97.69	1534 ± 167.3	734.7 ± 91.78	313 ± 195.3
